# Effective delivery of large genes to the retina by dual AAV vectors

**DOI:** 10.1002/emmm.201302948

**Published:** 2013-12-16

**Authors:** Ivana Trapani, Pasqualina Colella, Andrea Sommella, Carolina Iodice, Giulia Cesi, Sonia de Simone, Elena Marrocco, Settimio Rossi, Massimo Giunti, Arpad Palfi, Gwyneth J Farrar, Roman Polishchuk, Alberto Auricchio

**Affiliations:** 1 Telethon Institute of Genetics and Medicine (TIGEM)Naples, Italy; 2 Department of Ophthalmology, Second University of NaplesNaples, Italy; 3 Department of Veterinary Morphophysiology and Animal Production, University of BolognaBologna, Italy; 4 The School of Genetics & Microbiology, Trinity College DublinDublin, Ireland; 5 Medical Genetics, Department of Translational Medicine, Federico II UniversityNaples, Italy

**Keywords:** AAV, ABCA4, gene therapy, MYO7A, retina

## Abstract

Retinal gene therapy with adeno-associated viral (AAV) vectors is safe and effective in humans. However, AAV's limited cargo capacity prevents its application to therapies of inherited retinal diseases due to mutations of genes over 5 kb, like Stargardt's disease (STGD) and Usher syndrome type IB (USH1B). Previous methods based on ‘forced’ packaging of large genes into AAV capsids may not be easily translated to the clinic due to the generation of genomes of heterogeneous size which raise safety concerns. Taking advantage of AAV's ability to concatemerize, we generated dual AAV vectors which reconstitute a large gene by either splicing (trans-splicing), homologous recombination (overlapping), or a combination of the two (hybrid). We found that dual trans-splicing and hybrid vectors transduce efficiently mouse and pig photoreceptors to levels that, albeit lower than those achieved with a single AAV, resulted in significant improvement of the retinal phenotype of mouse models of STGD and USH1B. Thus, dual AAV trans-splicing or hybrid vectors are an attractive strategy for gene therapy of retinal diseases that require delivery of large genes.

## Introduction

Inherited retinal degenerations (IRDs), with an overall global prevalence of 1/2000 (Sohocki *et al*, 2001), are a major cause of blindness worldwide. Among the most frequent and severe IRDs are retinitis pigmentosa (RP), Leber congenital amaurosis (LCA), and Stargardt's disease (STGD), which are most often inherited as monogenic conditions. The majority of mutations causing IRDs occur in genes expressed in neuronal photoreceptors (PR), rods and/or cones in the retina (Dryja, 2001). No therapy is currently available for these blinding diseases.

Gene therapy holds great promise for the treatment of IRDs. Among the available gene transfer vectors, those based on the small adeno-associated virus (AAV) are most efficient at targeting both PR and retinal pigment epithelium (RPE) for long-term treatment upon a single subretinal administration (Colella *et al*, 2009; Vandenberghe & Auricchio, 2012). Recently, we and others have demonstrated that subretinal administration of AAV is well-tolerated and effective for improving vision in patients affected with type 2 LCA, which is caused by mutations in *RPE65*, a gene expressed in the RPE (Bainbridge *et al*, 2008; Maguire *et al*, 2008, 2009; Cideciyan *et al*, 2009; Simonelli *et al*, 2010). These results bode well for the treatment of other forms of LCA and IRDs in general. The availability of AAV vector serotypes such as AAV2/8, which efficiently targets PR (Allocca *et al*, 2007; Natkunarajah *et al*, 2008; Auricchio, 2011; Mussolino *et al*, 2011; Vandenberghe *et al*, 2011) and RPE, further supports this approach. However, a major limitation of AAV is its cargo capacity, which is thought to be limited to around 5 kb, the size of the parental viral genome (Hermonat *et al*, 1997; Dong *et al*, 2010a; Lai *et al*, 2010; Wu *et al*, 2010b; Wang *et al*, 2012). This limits the application of AAV gene therapy approaches for common IRDs that are caused by mutations in genes whose coding sequence (CDS) is larger than 5 kb (herein referred to as large genes). These include: (i) STGD (MIM#248200), the most common form of inherited macular degeneration caused by mutations in the *ABCA4* gene (CDS: 6822 bp; Allikmets, 1997), which encodes the all-trans retinal transporter located in the PR outer segment (Allikmets, 1997; Molday & Zhang, 2010); (ii) Usher syndrome type IB (USH1B; MIM#276900), the most severe form of RP and deafness caused by mutations in the *MYO7A* gene (CDS: 6648 bp; Millan *et al*, 2011) encoding the unconventional MYO7A, an actin-based motor expressed in both PR and RPE within the retina (Hasson *et al*, 1995; Liu *et al*, 1997; Gibbs *et al*, 2010).

Various strategies have been investigated to overcome the limitation of AAV cargo capacity. Several groups, including our own, have attempted to ‘force’ large genes into one of the many AAV capsids available by developing the so-called oversize vectors (Grieger & Samulski, 2005; Wu *et al*, 2007; Allocca *et al*, 2008). Although administration of oversize AAV vectors achieves therapeutically-relevant levels of transgene expression in rodent and canine models of human inherited diseases (Allocca *et al*, 2008; Monahan *et al*, 2010; Grose *et al*, 2012; Lopes *et al*, 2013), including the retina of the *Abca4*^−/−^ and *shaker 1* ( *sh1*) mouse models of STGD and USH1B (Allocca *et al*, 2008; Lopes *et al*, 2013), the mechanism underlying oversize AAV-mediated transduction remains elusive. In contrast to what we and others originally proposed (Grieger & Samulski, 2005; Wu *et al*, 2007; Allocca *et al*, 2008), oversize AAV vectors do not contain a pure population of intact large size genomes but rather a heterogeneous mixture of mostly truncated genomes ≤5 kb in length (Dong *et al*, 2010a; Lai *et al*, 2010; Wu *et al*, 2010b; Wang *et al*, 2012). Following infection, re-assembly of these truncated genomes in the target cell nucleus has been proposed as a mechanism for oversize AAV vector transduction (Dong *et al*, 2010a; Hirsch *et al*, 2010, 2013; Lai *et al*, 2010; Wu *et al*, 2010b). Independent of transduction mechanism and *in vivo* efficacy, the heterogeneity in oversize AAV genome sizes is a major limitation for their application in human gene therapy.

**Figure 1:**
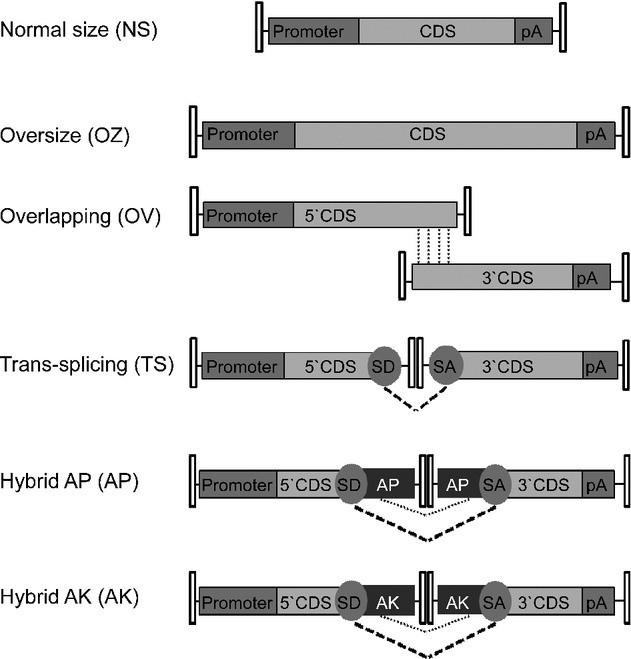
CDS, coding sequence; pA, poly-adenylation signal; SD, splicing donor signal; SA, splicing acceptor signal; AP, alkaline phosphatase recombinogenic region; AK, F1 phage recombinogenic region. Pointed lines show overlapping regions available for homologous recombination, dotted lines show the splicing occurring between SD and SA.

Alternatively, the inherent ability of AAV genomes to undergo intermolecular concatemerization (Duan *et al*, 1998) is exploited to transfer large genes *in vivo* by splitting a large gene expression cassette into halves (<5 kb in size), each contained in one of two separate (dual) AAV vectors (Yan *et al*, 2000; Duan *et al*, 2001; Ghosh *et al*, 2008). In the dual AAV trans-splicing strategy, a splice donor (SD) signal is placed at the 3′ end of the 5′-half vector and a splice acceptor (SA) signal is placed at the 5′ end of the 3′-half vector (Fig 1). Upon co-infection of the same cell by the dual AAV vectors and inverted terminal repeat (ITR)-mediated head-to-tail concatemerization of the two halves, trans-splicing results in the production of a mature mRNA and full-size protein (Yan *et al*, 2000). Trans-splicing has been successfully used to express large genes in muscle and retina (Reich *et al*, 2003; Lai *et al*, 2005). Alternatively, the two halves of a large transgene expression cassette contained in dual AAV vectors may contain homologous overlapping sequences (at the 3′ end of the 5′-half vector and at the 5′ end of the 3′-half vector, dual AAV overlapping), which will mediate reconstitution of a single large genome by homologous recombination (Duan *et al*, 2001). This strategy depends on the recombinogenic properties of the transgene overlapping sequences (Ghosh *et al*, 2006). A third dual AAV strategy (hybrid) is based on adding a highly recombinogenic region from an exogenous gene [i.e. alkaline phosphatase, AP (Ghosh *et al*, 2008, 2011)] to the trans-splicing vectors. The added region is placed downstream of the SD signal in the 5′-half vector and upstream of the SA signal in the 3′-half vector (Fig 1) in order to increase recombination between the dual AAVs.

To determine which AAV-based strategy most efficiently transduces large genes in the retina, we compared them side-by-side in HEK293 cells and in mouse and pig retina using *EGFP*, *ABCA4* or *MYO7A*. We then used the dual AAV trans-splicing and hybrid strategies, which were the most efficient for transducing PR as well as RPE, to rescue the retinal phenotype of the *Abca4*^−/−^ and *sh1* mouse models of STGD and USH1B.

## Results

### Generation of normal size, oversize and dual AAV vectors

We generated AAV oversize (OZ), dual AAV overlapping (OV), trans-splicing (TS) and hybrid vectors that included the therapeutic ABCA4-3xflag and MYO7A-HA coding sequences. The recombinogenic sequences included in the dual AAV hybrid vectors were based on either a previously reported region of the alkaline phosphatase transgene (AP, dual AAV hybrid AP; Ghosh *et al*, 2011) or a 77 bp sequence from the F1 phage genome (AK, dual AAV hybrid AK) that we found to be recombinogenic in previous *in vitro* experiments (Colella and Auricchio, unpublished data). We additionally generated AAV OZ and dual AAV vectors including the reporter EGFP sequence with the exception of the dual AAV OV approach since its efficiency relies on transgene-specific overlaps for reconstitution (Ghosh *et al*, 2006) and therefore can not be extrapolated from one gene to another. For EGFP we additionally generated single AAV vectors of normal size (NS) to compare levels of transgene expression from the various strategies. The constructs generated for production of all AAV vectors used in this study are listed in supplementary Table S1 and a schematic representation of the various approaches is depicted in Fig 1.

We used AAV2/2 vectors for the *in vitro* experiments, with the ubiquitous cytomegalovirus (CMV) or chicken beta-actin (CBA) promoters, which efficiently transduce HEK293 cells (Dong *et al*, 2010b). In the experiments performed *in vivo* in the retina, we used AAV2/8 vectors, which efficiently transduce RPE and PR (Allocca *et al*, 2007; Mussolino *et al*, 2011; Vandenberghe *et al*, 2011) but poorly infect HEK293 cells, and either the ubiquitous CBA and CMV promoters (Mussolino *et al*, 2011), or the RPE-specific vitelliform macular dystrophy 2 (VMD2; Esumi *et al*, 2004) or the PR-specific Rhodopsin (RHO) and Rhodopsin kinase (RHOK) promoters (Allocca *et al*, 2007; supplementary Table S1).

### Dual AAV vectors allow high levels of transduction *in vitro*

**Figure 2:**
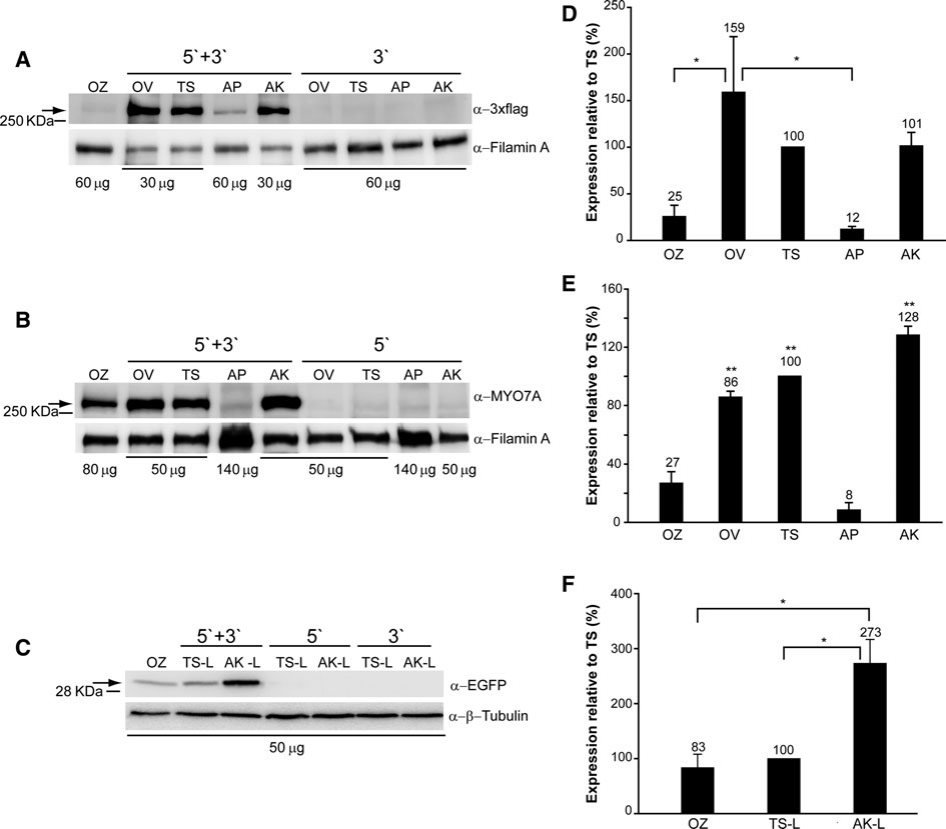
A–F Western blot analysis of HEK293 cells infected with AAV2/2 vectors encoding for ABCA4 (A, D), MYO7A (B, E) and EGFP (C, F). The Western blot images (A-C) are representative of and the quantifications (D-F) are from *n* = 4 (A, C, D, F) or *n* = 3 (B, E) independent experiments. OZ, AAV oversize; OV, dual AAV overlapping; TS, dual AAV trans-splicing; AP, dual AAV hybrid AP; AK, dual AAV hybrid AK; TS-L, dual AAV trans-splicing EGFP with a combined genome size similar to OZ-EGFP; AK-L, dual AAV hybrid AK EGFP with a combined genome size similar to OZ-EGFP; 5′+3′, cells co-infected with 5′-and 3′-half vectors; 5′, control cells infected with the 5′-half vector; 3′, control cells infected with the 3′-half vector; α-EGFP, Western blot with anti-EGFP antibody; α-3xflag, Western blot with anti-3xflag antibody; α-Myo7a, Western blot with anti-Myo7a antibody; α-β-Tubulin, Western blot with anti-β-Tubulin antibody, used as loading control; α-Filamin A, Western blot with anti-Filamin A antibody, used as loading control. * *P* ANOVA < 0.05; ** *P* ANOVA < 0.001. A–C The arrows indicate full-length proteins, the micrograms of proteins loaded are depicted under each lane, the molecular weight ladder is depicted on the left. D–F Quantification of ABCA4 (D), MYO7A (E) and EGFP (F) protein bands. The intensity of the ABCA4, MYO7A and EGFP bands was divided by the intensity of the Filamin A (D, E) or Tubulin (F) bands. The histograms show the expression of proteins as a percentage relative to dual AAV trans-splicing (TS) vectors, the mean value is depicted above the corresponding bar. Values are represented as mean ± standard error of the mean (s.e.m.). Data information: (E) The asterisks represent significant differences with both OZ and AP. (D–F) More details on the TS and TS-L variability as well as on the statistical analysis including specific statistical values can be found in the Western blot and Statistical analysis paragraphs of the Materials and methods section, respectively.

We initially compared the efficiency of the various OZ, dual AAV OV, TS and hybrid AP and AK strategies for AAV-mediated large gene transduction *in vitro* by infecting HEK293 cells with the AAV2/2 vectors [multiplicity of infection, m.o.i.: 10^5^ genome copies (GC)/cell of each vector] with ubiquitous promoters (CMV for ABCA4-3xflag, CBA for MYO7A-HA). Cell lysates were analyzed by Western blot with anti-3xflag (to detect ABCA4-3xflag, Fig 2A) or anti-Myo7a (Fig 2B) antibodies. All strategies resulted in the expression of proteins of the expected size. As predicted, no bands of the expected size were observed when only one of the dual AAV vectors was used for infection (Fig 2A and B). Quantification of ABCA4 and MYO7A expression (Fig 2D and E) showed that the dual AAV hybrid AP approach resulted in the lowest levels of transgene expression, while the dual AAV OV, TS and hybrid AK approaches were more efficient than the AAV OZ approach. We then confirmed this using the *EGFP* transgene. For this purpose we selected the best performing dual AAV strategies (TS and hybrid AK; we did not use the transgene-specific OV strategy with *EGFP*, which is a reporter gene) and further compared them to AAV OZ. We thus produced AAV2/2-CMV-OZ-EGFP vectors and for comparison-TS-and-AK-EGFP-L in which the combined dual AAV vector genome length is similar to that of AAV OZ (supplementary Table S1). We infected HEK293 cells with AAV2/2-CMV-EGFP vectors [multiplicity of infection, m.o.i.: 10^5^ genome copies (GC)/cell of each vector] and performed Western blot analysis of cell lysates with anti-EGFP antibodies (Fig 2C). Similarly to what observed with the ABCA4 and MYO7A transgenes, quantification of EGFP expression (Fig 2F) showed that dual AAV TS and hybrid AK approaches are more efficient than AAV OZ.

Then, we compared the efficiency of single AAV NS-EGFP to that of dual AAV TS and hybrid AK of similar size (supplementary Fig S1) by Western blot analysis of infected HEK293 cells. Quantification of EGFP expression showed that the levels achieved with dual AAV TS and hybrid AK were about 13–25-fold lower (7–4%) than with AAV NS (supplementary Fig S1).

### Dual AAV TS and hybrid AK but not OV vectors transduce mouse and pig photoreceptors

The enclosed and small subretinal space should favour co-infection and transduction of the same cell by two independent AAV vectors. To test this, we injected subretinally WT mice with AAV2/8-CMV-EGFP, AAV2/8-CMV-DsRed or a mixture of both vectors (dose of each vector/eye: 3 × 10^9^ GC) and harvested the eyes transduced by single or both vectors 3 weeks post-injection. Neural retinas, separated from the RPE, were dissociated and analyzed by flow cytometry. We found that 36 ± 6% of the labelled cells were positive for both EGFP and DsRed. However, since the number of DsRed^+^ only cells (8 ± 5%) was lower than the number of EGFP^+^ only cells (56 ± 8%) by counting the number of EGFP^+^/DsRed^+^ cells over the total number of transduced cells we may have under-estimated the co-transduction efficiency of the two vectors. Indeed, if we analyze the DsRed-positive cell populations, 82% of these were also EGFP-positive. This co-transduction efficiency is similar to that which we have reported previously (Palfi *et al*, 2012). To determine the co-transduction efficiency specifically in PR, and to test another red fluorescent reporter possibly more potent than DsRed, we repeated the injection in mice with a mixture of AAV2/8-CMV-EGFP and-RFP vectors (dose of each vector/eye: 1.4 × 10^9^ GC/eye) and analyzed co-transduction on retinal cryosections which allow to unequivocably identify PR from RPE (supplementary Fig S2). We found that 24 ± 2% of transduced PR expressed both EGFP and RFP. However, the number of RFP^+^ cells was still lower than the number of EGFP^+^ cells due to weaker RFP fluorescence compared to EGFP, and the percentage of co-transduction reaches 53 ± 4% when we consider the number of RFP^+^ cells that are also EGFP^+^.

**Figure 3:**
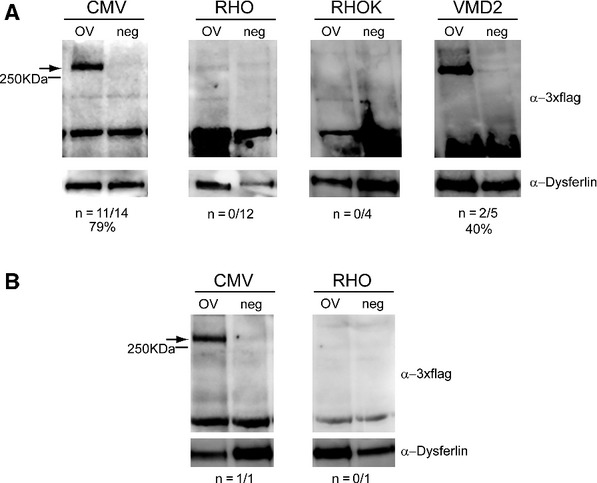
A–B Representative Western blot analysis of C57BL/6 (A) and Large White pig (B) retinal lysates 1 month following injection of dual AAV2/8 overlapping vectors encoding for ABCA4-3xflag (OV) or AAV2/8 vectors expressing EGFP (neg), under the control of the ubiquitous cytomegalovirus (CMV) promoter, the PR-specific Rhodopsin (RHO) and Rhodopsin kinase (RHOK) promoters, or the RPE-specific vitelliform macular dystrophy 2 (VMD2) promoter. The arrows indicate full-length proteins, the molecular weight ladder is depicted on the left, 150 μg of proteins were loaded in each lane. The number ( *n*) and percentage of ABCA4-positive retinas out of total retinas analyzed is depicted; α-3xflag, Western blot with anti-3xflag antibody; α-Dysferlin, Western blot with anti-Dysferlin antibody, used as loading control.

We then evaluated the best *in vitro* performing AAV-based systems for large gene transduction in the mouse retina. To test the dual AAV OV, which is transgene-specific, we used the therapeutic *ABCA4* and *MYO7A* genes (Fig 3 and data not shown). We used *EGFP* to evaluate the AAV OZ and the dual AAV TS and hybrid AK approaches (supplementary Fig S4).

Western blot analysis on retinal lysates, 1 month after subretinal delivery in C57BL/6 mice of the dual AAV OV vectors (dose of each vector/eye: 1.3 × 10^9^ GC), encoding ABCA4-3xflag from the ubiquitous CMV promoter, revealed robust protein expression (Fig 3A). To determine which cell type in the retina expressed ABCA4, we used dual AAV OV vectors (dose of each vector/eye: 1 × 10^9^ GC) that contained either the PR-specific RHO and RHOK, or the RPE-specific VMD2 promoters. We detected ABCA4 protein expression in retinas injected with the VMD2 but not with the RHO and RHOK promoters (Fig 3A). These results were also confirmed in the Large White pig retina (Fig 3B). The pig retina is an excellent model to evaluate vector efficiency because of its size, which is similar to the human retina, and because it is enriched with cones that are concentrated in a streak-like region whose cone density is comparable to that of the primate macula (Mussolino *et al*, 2011). We injected Large White pig subretinally with dual AAV OV vectors encoding ABCA4-3xflag (dose of each vector/eye: 1 × 10^10^ GC), and observed ABCA4 protein expression with the CMV but not the RHO promoter (Fig 3B). Similarly, subretinal administration of dual AAV OV vectors encoding MYO7A-HA resulted in weak MYO7A protein expression in the mouse retina with the ubiquitous CBA (dose of each vector/eye: 2.5 × 10^9^ GC) and no detectable expression with the RHO (dose of each vector/eye: 3.2 × 10^9^ GC) promoter (data not shown). To rule out that this was due to a lower transcriptional activity from either the RHO or RHOK promoters compared to CMV, we injected subretinally C57BL/6 mice with 1.5 × 10^9^ GC/eye of AAV2/8 vectors expressing *EGFP* from each of the promoters and found that CMV and RHO drive similarly robust PR transgene expression (supplementary Fig S3). Overall, these data suggest that the dual AAV OV approach is more efficient for large gene transfer to RPE than to PR.

To find an AAV-based strategy that efficiently transduces large genes in PR, which are a major target of gene therapy for IRDs including STGD and USH1B, we compared the retinal transduction properties of the AAV OZ with those of dual AAV TS and hybrid AK approaches, which in addition to dual AAV OV were the best performing dual AAV approaches *in vitro*. We used EGFP, which allowed us to easily localize transgene expression in the various retinal cell types, including PR, as well as to properly compare the levels of AAV-based large transgene transduction to those of a single AAV NS vector. C57BL/6 mice were initially injected subretinally with AAV OZ and dual AAV TS-L and hybrid AK-L vectors (dose of each vector/eye: 1.7 × 10^9^ GC), all encoding EGFP under the transcriptional control of the CMV promoter. One month later, fundus photographs showed that the highest levels of fluorescence were obtained with the dual AAV TS and hybrid AK approaches (supplementary Fig S4A). Fluorescence microscope analysis of retinal cryosections showed that detectable levels of RPE and PR transduction could be achieved with all approaches when combining the two half-vectors but not with each of them separately (supplementary Figs S4B and S5). However the levels of expression were higher, although variable, in the eyes injected with dual AAV TS-L and hybrid AK-L than in the eyes injected with AAV OZ vectors (supplementary Fig S4).

To test whether the levels and consistency of dual AAV-mediated transduction obtained can be improved by varying the ratio between the 5′-and 3′-half vectors, we injected subretinally C57BL/6 mice with dual AAV TS and hybrid AK vectors with different doses of 5′-and 3′-half vectors (supplementary Fig S6). We set as 1:1 the dose of 2.5 × 10^8^ GC/eye which is submaximal considering that the titers of our AAV preps range around 10^9^ GC/μl (supplementary Table S2) and that 1 μl is the maximum volume tolerated by the mouse subretinal space. Thus, 2.5 × 10^9^ GC/eye which is the high 1:1 dose (10:10) is the maximum dose which we can administer to the murine eye. We show that none of the various ratios tested outperforms the 1:1 ratio of the high dose of vectors (10:10) used so far (supplementary Fig S6). Therefore we used the optimal 10:10 dose and ratio in our following experiments.

**Figure 4:**
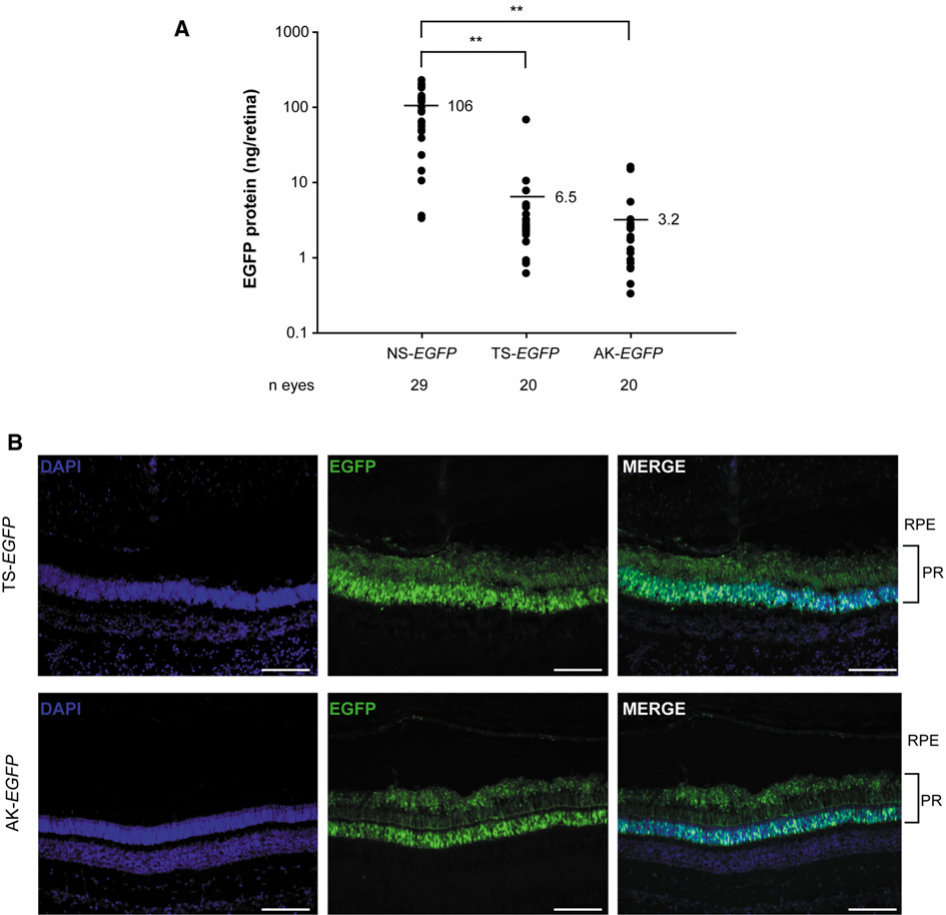
EGFP protein quantification by ELISA of retinas from C57BL/6 mice 1 month following subretinal injection of AAV2/8 vectors encoding for EGFP under the control of the PR-specific Rhodopsin (RHO) promoter. The scatter plot depicts EGFP protein levels from each retina; the mean value for each group is depicted and indicated with a solid line. The number ( *n*) of eyes analyzed is depicted under the *x* axis. ** *P* ANOVA<0.001. More details on the statistical analysis including specific statistical values can be found in the Statistical analysis paragraph of the Materials and methods section.Fluorescence analysis of retinal cryosections from Large White pigs 1 month following subretinal injection of AAV2/8 vectors encoding for EGFP under the control of the PR-specific RHO promoter. The pictures are representative of: *n* = 4 or *n* = 3 eyes injected with dual AAV TS or hybrid AK, respectively. The scale bar (100 μm) is depicted in the figure. NS, AAV normal size; TS, dual AAV trans-splicing; AK, dual AAV hybrid AK; DAPI, 4′,6′-diamidino-2-phenylindole staining; EGFP, native EGFP fluorescence; Merge, overlay of DAPI and EGFP pictures; RPE, retinal pigmented epithelium; PR, photoreceptors.

We then assessed PR-specific transduction levels in C57BL/6 mice following subretinal administration of dual AAV TS and hybrid AK vectors, which appears the most promising for large gene reconstitution in PR, as well as AAV NS vectors for comparison (dose of each vector/eye: 2.4 × 10^9^ GC). All vectors encoded EGFP under the transcriptional control of the PR-specific RHO promoter. One month after vector administration whole retina lysates were analyzed by ELISA to quantify EGFP protein levels (Fig 4A). Dual AAV TS and hybrid AK vectors reconstituted consistent EGFP expression in PR at levels on average about 16–33-fold lower (6–3%) than with AAV NS. However, these levels were variable, similarly to what observed in retinal histological sections (supplementary Fig S4B), and some of the eyes treated with dual AAV vectors had EGFP levels in the range of those achieved with AAV NS (Fig 4A). As expected, no detectable EGFP expression was measured by ELISA in injected retinas when only one of the dual AAV vectors encoding for EGFP was used ( *n* = 9: five eyes injected with 5′-half and four eyes with 3′-half; data not shown).

Thus, we conclude that dual AAV TS and hybrid AK strategies allow efficient mouse PR transduction although at levels which are lower than those obtained with an AAV NS. We then confirmed that subretinal administration of dual AAV TS and hybrid AK vectors transduced PR of Large White pigs (Fig 4B; dose of each vector/eye: 1 × 10^11^ GC).

### Dual AAV vectors improve the retinal phenotype of STGD and USH1B mouse models

To understand whether the levels of PR transduction obtained with the dual AAV TS and hybrid AK approaches may be therapeutically relevant, we investigated them in the retina of two mouse models of IRDs, STGD and USH1B, caused by mutations in the large *ABCA4* and *MYO7A* genes, respectively.

**Figure 5:**
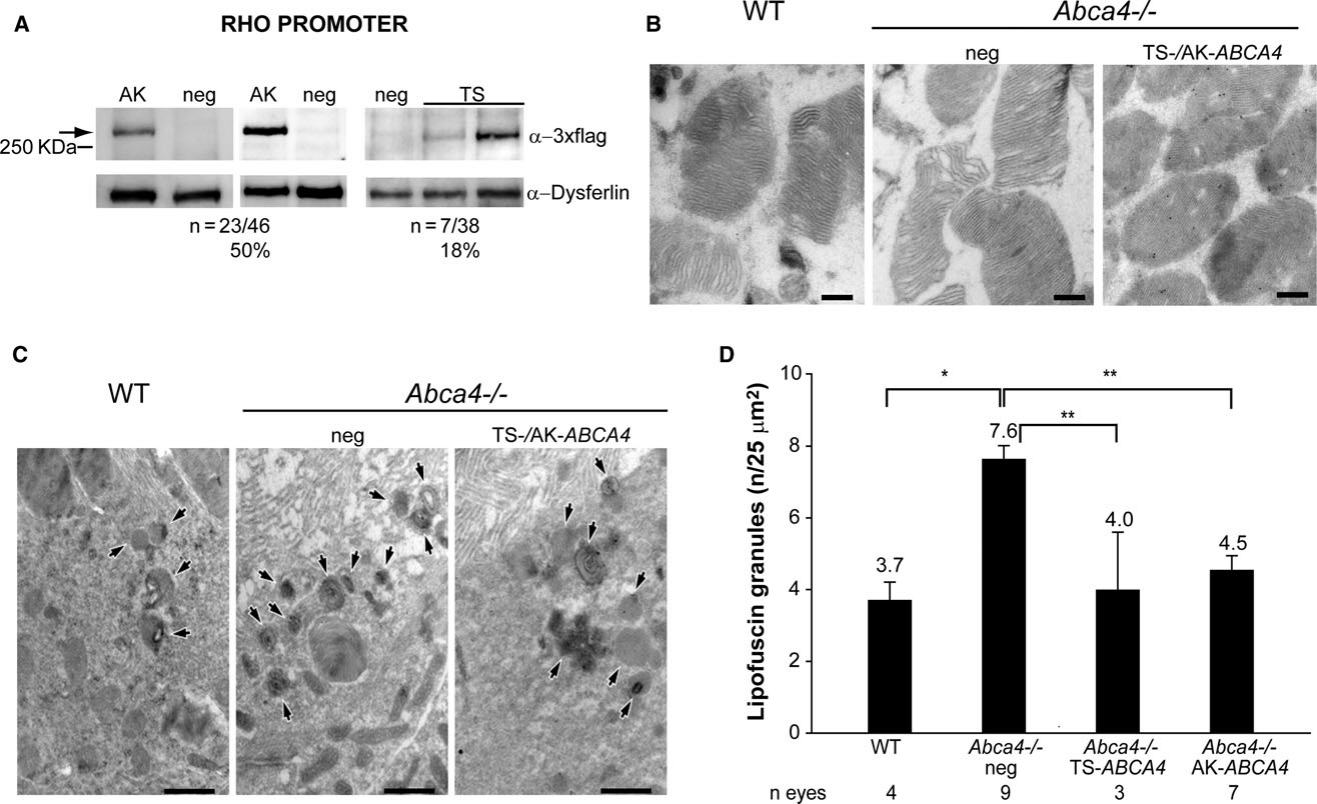
Representative Western blot analysis of C57BL/6 retinal lysates 1 month following the injection of dual AAV trans-splicing (TS) and hybrid AK (AK) vectors encoding for ABCA4 under the control of the PR-specific Rhodopsin promoter (RHO PROMOTER). The arrow points at full-length proteins, the molecular weight ladder is depicted on the left, 150 μg of protein were loaded in each lane. The number ( *n*) and percentage of ABCA4-positive retinas out of total retinas analyzed is depicted. AK, retinas injected with dual AAV hybrid AK vectors; TS, retinas injected with dual AAV TS vectors; neg, retinas injected with AAV vectors expressing *EGFP*, as negative controls; α-3xflag, Western blot with anti-3xflag antibody; α-Dysferlin, Western blot with anti-Dysferlin antibody, used as loading control.Representative pictures of immuno-electron microscopy analysis with anti-HA antibody of retinal sections from wild-type BALB/c (WT) and *Abca4*^−/−^ mice injected with either dual AAV or with negative control vectors. The black dots represent the immuno-gold labelling of the ABCA4-HA protein. The scale bar (200 nm) is depicted in the figure.Representative pictures of transmission electron microscopy analysis showing lipofuscin granules content in the RPE of WT and *Abca4*^−/−^ mice injected with either dual AAV or negative control vectors. The black arrows indicate lipofuscin granules. The scale bar (1.6 μm) is depicted in the figure.Quantification of the mean number of lipofuscin granules counted in at least 30 fields (25 μm^2^) for each sample. The number ( *n*) of eyes analyzed is depicted below each bar. The mean value is depicted above the corresponding bar. Values are represented as mean ± standard error of the mean (s.e.m.). * *P* ANOVA < 0.05; ** *P* ANOVA < 0.001. More details on the statistical analysis including specific statistical values can be found in the Statistical analysis paragraph of the Materials and methods section. Data information: (B-D) WT, BALB/c eyes; Abca4^−/−^ neg, Abca4^−/−^ eyes injected with either AAV vectors expressing EGFP ( *n* = 2) or the 5′-( *n* = 3) or 3′-( *n* = 4) half of the dual AAV hybrid AK vectors, as negative control (neg total n = 9); Abca4^−/−^ AK-ABCA4, mice injected with dual AAV hybrid AK vectors; Abca4^−/−^ TS-ABCA4, mice injected with dual AAV TS vectors.

Although the *Abca4*^−/−^ mouse model does not undergo severe PR degeneration (Wu *et al*, 2010a), the absence of the ABCA4-encoded all-trans retinal transporter in PR outer segments (Illing *et al*, 1997; Sun & Nathans, 1997) causes an accumulation of lipofuscin in PR as well as in RPE, as result of PR phagocytosis by RPE (Weng *et al*, 1999; Mata *et al*, 2001). As a consequence, both the number of lipofuscin granules in the RPE and the thickness of RPE cells are greater in *Abca4*^−/−^ mice than in control mice (Allocca *et al*, 2008; Radu *et al*, 2008; Ma *et al*, 2011; Conley *et al*, 2012; Han *et al*, 2012). In addition, *Abca4*^−/−^ mice also show delayed recovery from light desensitization (Weng *et al*, 1999; Maiti *et al*, 2006; Allocca *et al*, 2008; Radu *et al*, 2008; Han *et al*, 2012). Since *ABCA4* is expressed specifically in PR, we generated dual AAV TS and hybrid AK vectors encoding ABCA4-3xflag under the transcriptional control of the RHO promoter. These vectors were subretinally injected in wild-type C57BL/6 mice (dose of each vector/eye: 3–5 × 10^9^ GC) and 1 month later retinas were lysed and analyzed by Western blot with anti-3xflag antibodies (Fig 5A). Both approaches resulted in robust yet variable levels of ABCA4-3xflag expression. ABCA4-3xflag expression levels were more consistent in retinas treated with the dual AAV hybrid AK vectors (Fig 5A). No truncated and/or aberrant ABCA4 proteins were detected by Western blot analysis of C57BL/6 eyecups treated with dual AAV TS and hybrid AK vectors using anti-3xflag antibodies although two proteins (>100 KDa) smaller than the full length are produced *in vitro* following infection with either the single 5′-or 3′-half of both dual AAV approaches (supplementary Figs S7 and S8). In addition no evident signs of retinal toxicity were observed in *Abca4*^−/−^ mice at 8 months after treatment with dual AAV TS and hybrid AK vectors by conventional histological analysis (supplementary Fig S9A).

**Figure 6:**
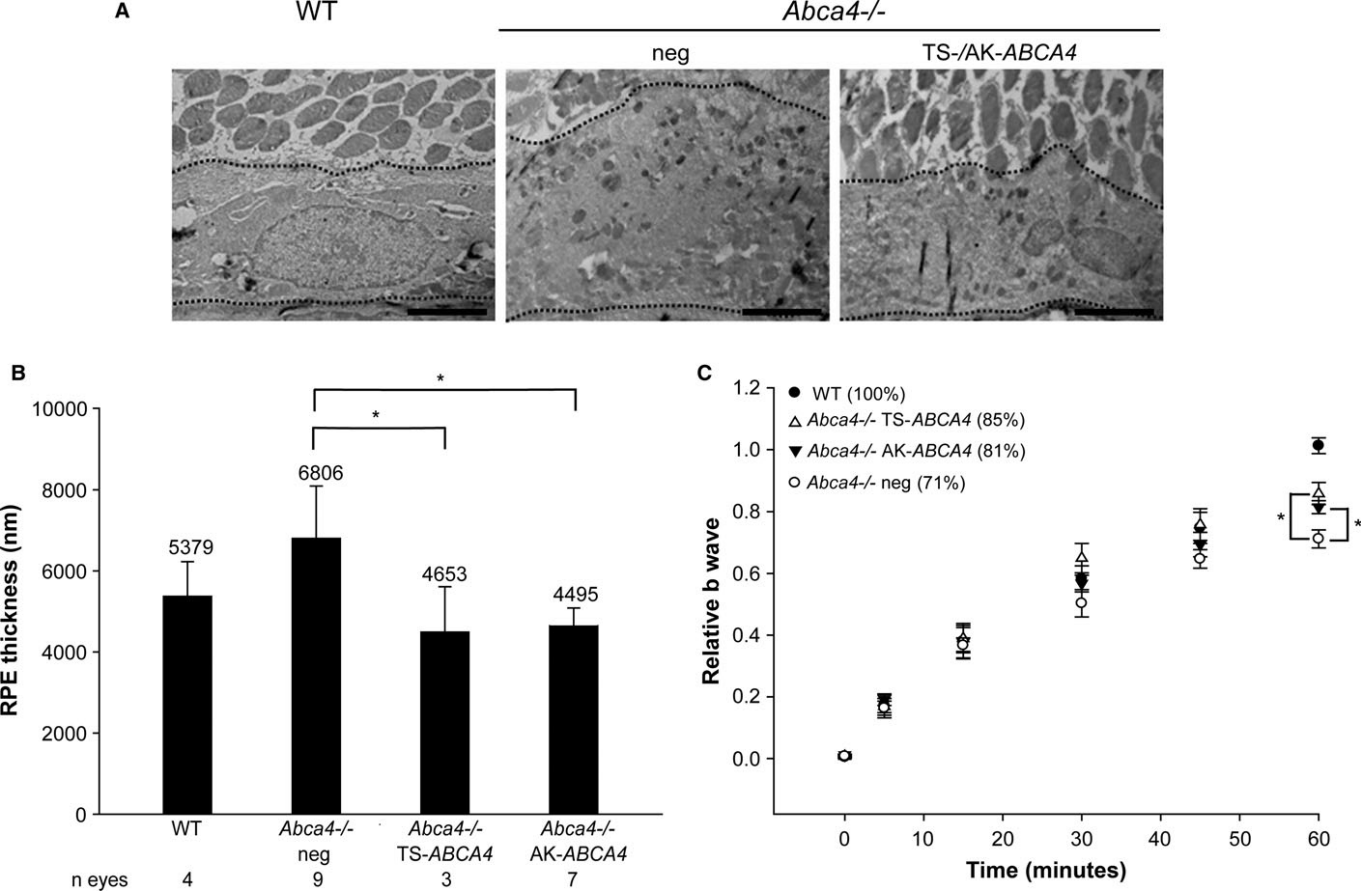
Representative pictures of transmission electron microscopy analysis of retinal sections from wild-type BALB/c (WT) and *Abca4*^−/−^ mice injected with dual AAV trans-splicing (TS-*ABCA4*) and hybrid AK vectors (AK-*ABCA4*) or with either AAV vectors expressing *EGFP* or 5′-or 3′-half of the dual hybrid AK vectors (neg), as negative controls. The dotted lines indicate the edges of RPE cells. The scale bar (3.8 μm) is depicted in the figure.Quantification of the mean RPE thickness counted in at least 30 fields for each sample. The number ( *n*) of eyes analyzed is depicted below each bar. *Abca4*^−/−^ neg includes *Abca4*^−/−^ mice injected with either AAV vectors expressing *EGFP* ( *n* = 2) or 5′-( *n* = 3) or 3′-( *n* = 4) half of the dual hybrid AK vectors (neg total *n* = 9). The mean value is depicted above the corresponding bar. Values are represented as mean ± standard error of the mean (s.e.m.). * *P* ANOVA < 0.05.Recovery from light desensitization in 3 month-old *Abca4*^−/−^ and BALB/c mice at 6 weeks post-injection. The relative b-wave is the ratio between the post-and the pre-desensitization b-wave amplitudes both evoked by 1 cd s/m^2^. The time refers to the minutes post-desensitization. The mean recovery (%) at 60 min is depicted. Data information: (A-C) WT, BALB/c eyes ( *n* = 4); *Abca4*^−/−^ TS-*ABCA4*, eyes injected with dual AAV TS vectors ( *n* = 5); *Abca4*^−/−^ AK-*ABCA4*, AAV hybrid AK vectors ( *n* = 5); *Abca4*^−/−^ neg, *Abca4*^−/−^ mice either not injected ( *n* = 2) or injected with the 5′-half of the dual AAV TS ( *n* = 3) or hybrid AK ( *n* = 2) vectors (neg total *n* = 7). Values are represented as mean ± standard error of the mean (s.e.m.). * *P* ANOVA < 0.05. More details on the statistical analysis including specific statistical values can be found in the Statistical analysis paragraph of the Materials and methods section.

To evaluate the biological and therapeutic activity of the recombinant ABCA4 protein produced by dual AAV vectors, 1 month-old albino *Abca4*^−/−^ mice were injected subretinally with the dual AAV TS and hybrid AK RHO-*ABCA4*-HA vectors (dose of each vector/eye: 1–3 × 10^9^ GC). Three months later, eyes were harvested and immuno-electron microscopy analysis with anti-hemagglutinin (HA) antibodies of retinal sections confirmed that immunogold particles were correctly localized in PR outer segments only in animals that were injected with the combination of 5′ and 3′ dual AAV TS and hybrid AK vectors (Fig 5B). To assess the functionality of the ABCA4 protein expressed by the dual AAV vectors, we measured *Abca4*^−/−^ retinal lipofuscin accumulation and recovery from light desensitization. To assess the former we performed transmission electron microscopy analysis to measure the number of RPE lipofuscin granules (Fig 5C and D) and RPE thickness (Fig 6A and B). Both were greater in the retina of *Abca4*^−/−^ mice injected with control vectors (independently of the size of the control constructs, supplementary Fig S10) than in the retina of wild-type, age-matched BALB/c controls, and were significantly reduced or normalized in the eyes injected either with the therapeutic dual AAV TS or hybrid AK vectors (Figs 5C and D and 6A and B). We additionally attempted at measuring A2E, the major component of lipofuscin granules, by HPLC (Parish *et al*, 1998; Ben-Shabat *et al*, 2002; Allocca *et al*, 2008), however these measurements were inconsistent, even between affected and normal retinas (data not shown), thus we were not able to use these techniques in our rescue experiments. Importantly, the eyes treated with dual AAV TS and hybrid AK vectors showed improved recovery from light desensitization when compared to eyes treated with control vectors (dose of each vector/eye: 1.2 × 10^9^ GC, Fig 6C), independently of the size of the control constructs [Student's *t*-test *P* value of dual AAV-EGFP-L ( *n* = 2 TS-L, *n* = 4 AK-L; total *n* = 6) versus dual AAV-EGFP ( *n* = 5 TS, *n* = 4 AK; total *n* = 9): 0.23].

**Figure 7:**
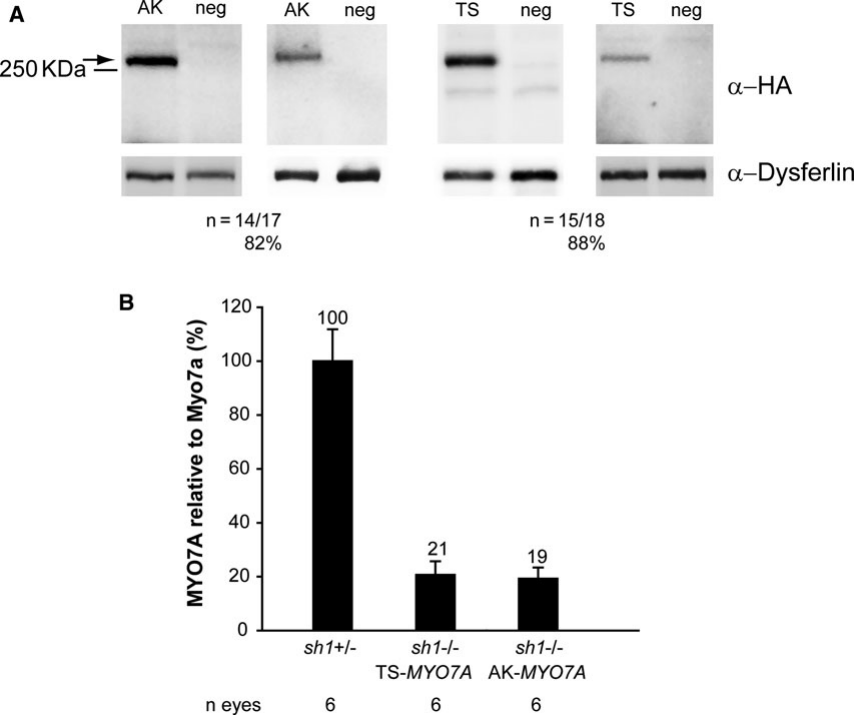
Representative Western blot analysis of C57BL/6 eyecups 1 month following the injection of dual AAV trans-splicing (TS) and hybrid AK (AK) vectors encoding for MYO7A-HA under the control of the ubiquitous chicken beta-actin (CBA) promoter. The arrow indicates full-length proteins, the molecular weight ladder is depicted on the left, 100 μg of proteins were loaded in each lane. The number ( *n*) and percentage of MYO7A-positive eyecups out of total eyecups analyzed is depicted. AK, eyes injected with dual AAV hybrid AK vectors; TS, eyes injected with dual AAV TS vectors; neg, eyes injected with either 5′-or 3′-half of the dual AAV TS and hybrid AK vectors; α-HA, Western blot with anti-hemagglutinin (HA) antibody; α-Dysferlin, Western blot with anti-Dysferlin antibody, used as loading control.Quantification of MYO7A levels expressed from dual AAV vectors in *sh1*^−/−^ eyecups relative to endogenous Myo7a expressed in littermate *sh1*^+/–^ eyecups. *sh1*^−/−^ eyes were injected with dual AAV TS and hybrid AK vectors encoding MYO7A under the control of the CBA promoter and analyzed 1.5 months later. *sh1*^+/–^ eyes were injected with AAV vectors expressing EGFP. The number ( *n*) of eyes analyzed is depicted below each bar. The quantification was performed by Western blot analysis using the anti-Myo7a antibody and measurements of MYO7A and Myo7a band intensities normalized to Dysferlin. The histograms show the expression of MYO7A protein as percentage relative to *sh1*^+/–^ Myo7a; the mean value is depicted above the corresponding bars. Values are represented as mean ± standard error of the mean (s.e.m.).

We then tested the efficacy of dual AAV-mediated *MYO7A* gene transfer in the retina of *sh1* mice, the most commonly used model of USH1B (Liu *et al*, 1997, 1998, 1999; Lillo *et al*, 2003; Gibbs *et al*, 2010). In *sh1* mice, a deficiency in the motor Myo7a causes the mis-localization of RPE melanosomes (Liu *et al*, 1998), which do not enter into the RPE apical microvilli, and the accumulation of rhodopsin at the PR connecting cilium (Liu *et al*, 1999). *MYO7A* is highly expressed in the RPE and to a lesser extent in PR (Hasson *et al*, 1995; Liu *et al*, 1997), therefore we used dual AAV TS and hybrid AK vectors expressing MYO7A-HA under the transcriptional control of the ubiquitous CBA promoter. One month-old wild-type C57BL/6 mice were injected with the dual AAV vectors (dose of each vector/eye: 1.7 × 10^9^ GC) and eyecup lysates were evaluated 1 month later using Western blot analysis with anti-HA antibodies. Results showed similarly robust and consistent levels of MYO7A expression in retinas treated with both approaches (Fig 7A). Taking advantage of our anti-Myo7a antibody able to recognize both murine and human MYO7A (although with potentially different affinity for the two orthologous proteins), we compared the levels of MYO7A achieved following delivery of dual AAV vectors to the *sh1*^−/−^ eye to those expressed endogenously in the *sh1*^+/−^ eye (Fig 7B). The levels of human MYO7A driven by the CBA promoter 1 month after treatment (Fig 7B; dose of each vector/eye: 1.7 × 10^9^ GC) were 19–21% of endogenous Myo7a expressed in both RPE and PR (Fig 7B) and these remained similar at 9 months after vector delivery (MYO7A retinal levels after subretinal delivery of dual AAV-TS, quantified on Western blot of eyecup lysates as in Fig 7B: 19 ± 6% of endogenous Myo7a, *n* = 5). Notably, subretinal delivery of dual AAV TS and hybrid AK resulted in efficient expression of human MYO7A specifically in PR when using the RHO promoter (supplementary Fig S11). No MYO7A proteins of size different from the full-length were detected by Western blot analysis of *sh1*^−/−^ eyecups treated with either dual AAV TS or hybrid AK vectors. However, two proteins smaller than the full length MYO7A (<130 KDa) are detected *in vitro* following infection with either the single 5′-or 3′-half of both dual AAV approaches (supplementary Fig S12 and S13).

**Figure 8:**
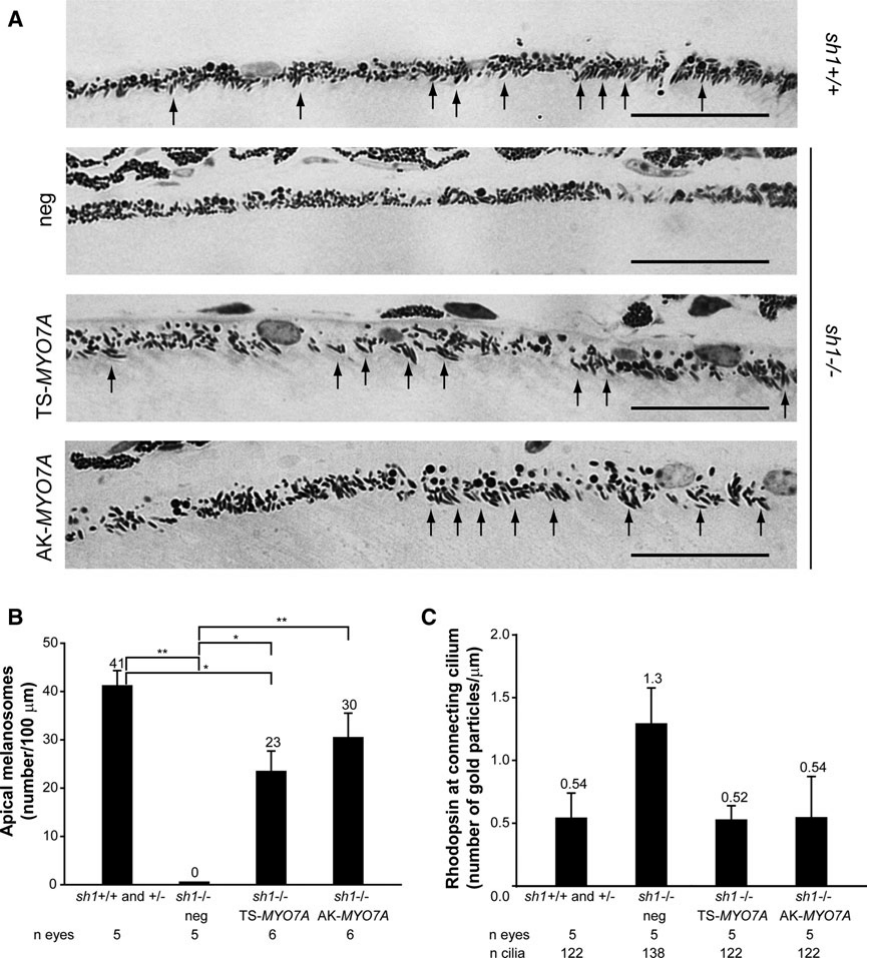
Semi-thin retinal sections representative of both *sh1*^+/+^ and *sh1*^+/–^ eyes ( *sh1*^+/+^) injected with AAV vectors expressing *EGFP* and of *sh1*^−/−^ eyes injected with dual AAV trans-splicing (TS-*MYO7A*), hybrid AK (AK-*MYO7A*) or the 5′-or 3′-half vectors (neg), as negative controls. The arrows point at correctly localized melanosomes, the scale bar (10 μm) is depicted in the figure.Quantification of melanosome localization in the RPE villi of *sh1* mice 2–3 months following subretinal delivery of dual AAV vectors. The *n* of eyes analyzed is depicted below each bar. The quantification is depicted as the mean number of apical melanosomes/100 μm, the mean value is depicted above the corresponding bar. *sh1*^−/−^ neg includes *sh1*^−/−^ eyes injected with AAV vectors expressing either 5′-( *n* = 1) or 3′-( *n* = 2) half of the dual TS vectors, or 5′-half ( *n* = 2) of the dual hybrid AK vectors (neg total *n* = 5). Values are represented as mean ± standard error of the mean (s.e.m.). * *P* ANOVA < 0.05, ** *P* ANOVA < 0.001.Quantification of the number of rhodopsin gold particles at the PR connecting cilium of *sh1* mice 2–3 months following subretinal delivery of dual AAV vectors. The *n* of eyes and connecting cilia analyzed is depicted below each bar. *sh1*^−/−^ neg includes *sh1*^−/−^ eyes injected with AAV vectors expressing either 5′-half of the dual TS vectors ( *n* = 3) or 5′-half ( *n* = 2) of the dual hybrid AK vectors (neg total *n* = 5). The quantification is depicted as the mean number of gold particles per length of connecting cilium, the mean value is depicted above the corresponding bar. Values are represented as mean ± standard error of the mean (s.e.m.). More details on the statistical analysis including specific statistical values can be found in the Statistical analysis paragraph of the Materials and methods section.

To test the ability of MYO7A expressed from dual AAV vectors to rescue the defects of the *sh1*^−/−^ retina, we evaluated RPE melanosome (Fig 8A and B) and rhodopsin localization (Fig 8C) following subretinal injection of dual AAV TS and hybrid AK CBA-*MYO7A* vectors (dose of each vector/eye: 1.7 × 10^9^ GC) in 1 month-old *sh1*^−/−^ mice. Unlike in unaffected mice, the *sh1*^−/−^ melanosomes do not enter the RPE apical microvilli (Fig 8A and B), this was significantly improved after the delivery of either dual AAV TS or hybrid AK vectors encoding *MYO7A* (Fig 8A and B).

We also found that the number of rhodopsin particles at the connecting cilium is greater in *sh1*^−/−^ retinas treated with control vectors (independently of the size of the control constructs, supplementary Fig S14) than in unaffected *sh1* (Fig 8C and supplementary Fig S14). This was reduced in *sh1*^−/−^ retinas treated with dual AAV TS and hybrid AK vectors expressing MYO7A, although the differences were not statistically significant (Fig 8C). Notably, the improvement of the *sh1*^−/−^ retinal defects is associated with a normal retinal architecture (supplementary Fig S9B) which further suggests that subretinal delivery of dual AAV TS and hybrid AK vectors does not induce evident retinal toxicity.

## Discussion

While AAV-mediated gene therapy is effective in animal models and in patients with inherited blinding conditions (Jacobson *et al*, 2006; Bainbridge *et al*, 2008; Maguire *et al*, 2008, 2009; Cideciyan *et al*, 2009; Simonelli *et al*, 2010), its application to diseases affecting the retina and requiring a transfer of genes larger than 5 kb (referred to as large genes) is inhibited by AAV limited cargo capacity. To overcome this, we compared the efficiency of various AAV-based strategies for large gene transduction including: AAV OZ and dual AAV OV, TS, hybrid AP and AK approaches *in vitro* and in mouse and pig retina. Our *in vitro* and *in vivo* results show that the dual AAV strategies we tested (with the exception of the dual AAV hybrid AP) outperform AAV OZ vectors in terms of transduction levels. This may be explained by: (i) the homogeneous size of the dual AAV genome population when compared to AAV OZ genomes, which may favour the generation of transcriptionally active large transgene expression cassettes; (ii) the small volume of the subretinal space, which we show favours infection and transduction of the same cell by two independent AAV vectors. Although this has been suggested by previous work in the retina with either dual AAV TS vectors carrying the lacZ reporter gene (Reich *et al*, 2003) or with two single vectors encoding different fluorescent reporter proteins (Palfi *et al*, 2012), our study represents the first comprehensive comparison in the retina of the dual AAV strategies reported so far, as well as the first demonstration of their efficacy in animal models of common inherited blinding conditions.

The dual AAV OV approach is particularly interesting when compared to the TS or hybrid AK approaches, which appear similarly efficient *in vitro*, as dual AAV OV vectors only contain sequences belonging to the therapeutic transgene expression cassette. However, when we administered dual AAV OV vectors to the subretinal space of adult mice and pigs, we were able to detect expression of the large ABCA4 and MYO7A proteins only when the ubiquitous or the RPE-specific promoters, but not the PR-specific promoters, were used. This may suggest that the homologous recombination required for dual AAV OV reconstitution is more efficient in RPE than PR. This is consistent with the low levels of homologous recombination reported in post-mitotic neurons (Fishel *et al*, 2007) and may partially explain the low levels of dual AAV OV-mediated MYO7A transduction recently reported by other groups (Lopes *et al*, 2013). We conclude that subretinal administration of dual AAV OV vectors should not be used for large gene transfer to PR, although we can not exclude that sequences that are more recombinogenic than those included in our dual AAV OV *ABCA4* and *MYO7A* vectors may allow efficient homologous recombination in PR.

Dual AAV TS and hybrid AK approaches efficiently transduce mouse and pig PR, differently from what we observed with dual AAV OV. This is consistent with the knowledge that the mechanism of large gene reconstitution mediated by dual AAV TS and hybrid AK approaches may be via ITR-mediated head-to-tail rejoining (Duan *et al*, 1998; Yan *et al*, 2005; Ghosh *et al*, 2008) rather than homologous recombination.

The transduction levels provided by the dual hybrid AK are superior to those of the TS approach in mouse PR but not RPE. This was evident when using the large *ABCA4* and *MYO7A* but not the small *EGFP* transgene, suggesting an advantage of including the AK sequence for large transgene reconstitution in cells like PR which are more difficult to target by AAV2/8 than RPE.

Differently from what we have observed, Hirsch *et al* (2013) have recently shown that AAV OZ vectors can reconstitute a large reporter gene (6.2 kb) in the retina of mice more efficiently than dual AAV TS vectors. The following factors may account for this: (i) the design of the dual AAV vectors which may result in lower transduction levels in Hirsch *et al* than in our case; (ii) the purification of the AAV OZ vectors by Hirsch *et al* that may promote the selection in the viral preparation of genomes with higher transduction properties than in our preparations; (iii) the use of a shorter transgene by Hirsch *et al* than by us that gives rise to genomes with longer overlaps which can positively influence AAV OZ transduction. Our results also differ from those of another group that has reported that dual AAV hybrid AP outperforms TS (Ghosh *et al*, 2011). While we have generated our dual hybrid AP vectors based on the description provided in that publication, it is possible that minor differences in the AP sequences used may account for the different transduction levels we have observed.

The levels of mouse PR transduction we achieved with dual AAV TS and hybrid AK are lower than with single NS vectors. However, we show that in the case of MYO7A, retinal transgene expression levels are about 20% of endogenous. These may be effective for treating inherited blinding conditions that require relatively low levels of transgene expression, i.e. diseases inherited as autosomal recessive. Indeed, we show that subretinal delivery of dual AAV TS and hybrid AK improves and even normalizes the retinal defects of two animal models of IRDs, STGD and USH1B, which are due to mutations in large genes and are attractive targets of gene therapy. Importantly, both levels and consistency of dual AAV-mediated large transgene expression could be improved by directing the productive head-to-tail genome concatemerization by either using heterologous ITRs (Yan *et al*, 2007) or by adding oligos to the injection solution (Hirsch *et al*, 2009).

Single normal size AAV vectors ensure multi-year retinal gene expression after a single vector administration (Testa *et al*, 2013). The data we have obtained so far from dual AAV vectors up to 9 months after retinal gene delivery, the last time point of our analysis, suggest that longevity of transgene expression may be similar between single normal size and dual AAV vectors.

The genome size of dual AAV vectors is homogeneous, which means identity and safety issues related to their use should be less considerable than those related to AAV OZ vectors, which have heterogeneous genome sizes. However, the possibility that delivery of dual AAV vectors results in the production of aberrant proteins in the retina, i.e. truncated proteins from the 5′-half vector that contains the promoter sequence and/or from the 3′-half vector due to the low promoter activity of the ITRs (Flotte *et al*, 1993) must be considered. Our results show that proteins smaller than the full-length are produced from the 5′-and 3′-halves of dual AAV vectors *in vitro* but not *in vivo* in the retina where only full-length ABCA4 and MYO7A proteins are detected. The production of properly spliced full length proteins by dual AAV vectors is corroborated by the successful amplification of the full size ABCA4 and MYO7A mRNA retro transcribed from cells infected with dual AAV TS and hybrid AK vectors (data not shown). Indeed the transcript sequences confirmed that intermolecular AAV joining and splicing occurred correctly (data not shown), as expected by the size of the full length proteins detected by Western blot. In addition, we detected neither ERG (data not shown) nor retinal histological abnormalities in both *Abca4*^−/−^ and *sh1*^−/−^ mice that we followed up to 3–8 months after dual AAV vector delivery. Future long-term safety studies as well as sensitive proteomic profiling will help to better define if any toxicity derives from intraocular administration of dual AAV vectors.

In conclusion, we found that dual AAV vectors are efficient both *in vitro* and in the retina *in vivo*. While dual AAV OV vectors efficiently transduce RPE, they do not transduce PR, whereas dual AAV TS and hybrid AK approaches drive efficient large gene reconstitution in both cell types. Administration of dual AAV TS and hybrid AK approaches improved the retinal phenotype of mouse models of STGD and USH1B, providing evidence of the efficacy of these strategies for gene therapy of these and other blinding conditions, which require large gene transfer to PR as well as RPE.

## Materials and Methods

### Generation of AAV vector plasmids

The plasmids used for AAV vector production were derived from either the pZac2.1 (Gao *et al*, 2000) or pAAV2.1 (Auricchio *et al*, 2001) plasmids that contain the inverted terminal repeats (ITRs) of AAV serotype 2 (supplementary Table S1). Normal size and oversize AAV vector plasmids contained full length expression cassettes including the promoter, the full-length transgene CDS and the poly-adenylation signal (pA; supplementary Table S1). The two separate AAV vector plasmids (5′ and 3′) required to generate dual AAV vectors contained either the promoter followed by the N-terminal portion of the transgene CDS (5′ plasmid) or the C-terminal portion of the transgene CDS followed by the pA signal (3′ plasmid, supplementary Table S1). Normal size EGFP plasmids were generated by cloning the EGFP CDS of pAAV2.1-CMV-EGFP plasmid (720 bp; Auricchio *et al*, 2001) in pZac2.1 (Gao *et al*, 2000); oversize EGFP was generated from pAAV2.1-CMV-EGFP (Auricchio *et al*, 2001) by inserting a DNA stuffer sequence of 3632 bp from human *ABCA4* (NM_000350.2, bp 1960-5591) upstream of the CMV promoter and a second DNA stuffer sequence of 3621 bp, composed of: murine *ABCA4* (NM_007378.1, 1066-1 and 7124-6046 bp; 2145 total bp) and human *Harmonin* (NM_153676.3 131-1606 bp; 1476 total bp), downstream of the pA signal. To generate dual AAV vector plasmids, the EGFP CDS (720 bp) was split into two constructs: one containing the N-terminal CDS (PMID: 9759496, bp 1-393) and the other containing the C-terminal CDS (PMID: 9759496, bp 394-720). The stuffer sequences flanking the EGFP expression cassette in the EGFP OZ plasmid were used to generate dual AAV-TS-L and dual AAV-AK-L plasmids (supplementary Table S1) with a combined (5′+3′) genome length similar to the EGFP OZ construct size.

The oversize *ABCA4* plasmids contained the full-length human *ABCA4* CDS (NM_000350.2, bp 105-6926), while the oversize *MYO7A* plasmids contained the full-length human *MYO7A* CDS from isoform 1 (NM_000260.3, bp 273-6920). To generate plasmids for dual AAV OV vectors the *ABCA4* and *MYO7A* CDS were split into two constructs, one containing N-terminal CDS ( *ABCA4*: NM_000350.2, bp 105-3588; *MYO7A*: NM_000260.3, bp 273-3782) and the other containing C-terminal CDS ( *ABCA4*: NM_000350.2, bp 2819-6926; *MYO7A*: NM_000260.3, bp 2913-6920). Therefore, the region of homology shared by overlapping vector plasmids was 770 bp for *ABCA4* and 870 bp for *MYO7A*. To generate trans-splicing and hybrid vector plasmids the *ABCA4* and *MYO7A* CDS were split at a natural exon-exon junction. *ABCA4* was split between exons 19-20 (5′ half: NM_000350.2, 105-3022 bp; 3′ half: NM_000350.2, bp 3023-6926) and *MYO7A* was split between exons 24-25 (5′ half: NM_000260.3, bp 273-3380; 3′ half: NM_000260.3, bp 3381-6926). The ABCA4 and MYO7A proteins were both tagged at their C-terminus: ABCA4 with either the 3xflag or hemagglutinin (HA) tag; MYO7A with the HA tag only. In addition, the ABCA4 protein was tagged at both N-(amino acidic position 590) and C-termini with the 3xflag tag for the experiments shown in supplementary Figs S7 and S8. The splice donor (SD) and splice acceptor (SA) signals contained in dual trans-splicing and hybrid AAV vector plasmids are as follows: 5′-GTAAGTATCAAGGTTACAAGACAGGTTTAAGGAGACCAATAGAAACTGGGCTTGTCGAGACAGAGAAGACTCTTGCGTTTCT-3′ (SD); 5′-GATAGGCACCTATTGGTCTTACTGACATCCACTTTGCCTTTCTCTCCACAG-3′ (SA). The recombinogenic sequence contained in hybrid AP vector plasmids was derived from alkaline phosphatase (AP) gene (NM_001632, bp 823-1100), as previously described (Ghosh *et al*, 2011). The recombinogenic sequence contained in hybrid AK vector plasmids was derived from the phage F1 genome (Gene Bank accession number: J02448.1; bp 5850-5926). The AK sequence is: 5′-GGGATTTTGCCGATTTCGGCCTATTGGTTAAAAAATGAGCTGATTTAACAAAAATT TAACGCGAATTTTAACAAAAT-3′. To generate the pZac2.1-CMV-RFP plasmid, the monomeric RFP CDS (Campbell *et al*, 2002; 675 bp) was amplified and cloned in pZac2.1-CMV-EGFP plasmid using EcoRI and BamHI restriction sites. The pAAV2.1-CMV-DsRed plasmid (Palfi *et al*, 2012) was obtained by cloning the DsRed gene from the pDsRed-Express2 plasmid (Clontech, Saint-Germain-en-Laye, France) in the pAAV-MCS plasmid (Stratagene, La Jolla, CA, USA).

The ubiquitous CMV promoter is that contained in pZac2.1 (Gao *et al*, 2000) or pAAV2.1-CMV-EGFP (Auricchio *et al*, 2001); the ubiquitous CBA promoter was derived from pAAV2.1-CBA-EGFP (Mussolino *et al*, 2011), the PR-specific human RHO and RHOK promoters were derived from pAAV2.1-RHO-EGFP and pAAV2.1-RHOK-EGFP, respectively (Allocca *et al*, 2007); the RPE-specific VMD2 promoter (NG_009033.1, bp 4870-5470) corresponds to the previously described EcoRI-XcmI promoter fragment (Esumi *et al*, 2004) and was amplified by human genomic DNA. The details of cloning strategies as well as plasmid sequences are available upon request.

### AAV vector production and characterization

AAV vectors were produced by the TIGEM AAV Vector Core by triple transfection of HEK293 cells followed by two rounds of CsCl_2_ purification (Doria *et al*, 2013). For each viral preparation, physical titers [genome copies (GC)/ml] were determined by averaging the titer achieved by dot-blot analysis (Drittanti *et al*, 2000) and by PCR quantification using TaqMan (Applied Biosystems, Carlsbad, CA, USA; Doria *et al*, 2013). The probes used for dot-blot and PCR analyzes were designed to anneal with either the ITRs or regions within 1 kb from the ITRs. No statistically significant differences were found in the titers (GC/ml) of NS AAV2/2 or AAV2/8 compared to those of dual AAV OV, TS and hybrid vectors (supplementary Table S2). In addition alkaline Southern blot analysis of viral DNA extracted from several dual AAV OV, TS and hybrid AK preps showed that the AAV genome is homogeneous and its size corresponds to that expected independently of the presence of recombinogenic elements in the dual AAV vectors (supplementary Fig S15). However, since recombination is sequence-specific, the conclusions from supplementary Fig S15 can not be directly extrapolated to other vectors. The alkaline Southern blot analysis shown in supplementary Fig S15 was carried out as follows: 3 × 10^10^ GC of viral DNA were extracted from AAV particles. To digest unpackaged genomes, the vector solution was resuspended in 240 μl of PBS pH 7.4 1× (GIBCO; Invitrogen S.R.L., Milan, Italy) and then incubated with 1 U/μl of DNase I (Roche, Milan, Italy) in a total volume of 300 μl containing 40 mM TRIS–HCl, 10 mM NaCl, 6 mM MgCl2, 1 mM CaCl_2_ pH 7.9 for 2 h at 37°C. The DNase I was then inactivated with 50 mM EDTA, followed by incubation with proteinase K and 2.5% *N*-lauroyl-sarcosil solution at 50°C for 45 min to lyse the capsids. The DNA was extracted twice with phenol-chloroform and precipitated with two volumes of ethanol 100 and 10% sodium acetate (3 M, pH 7). Alkaline agarose gel electrophoresis and blotting were performed as previously described (Sambrook & Russell, 2001). Ten microlitres of the 1 kb DNA ladder (N3232L; New England Biolabs, Ipswich, MA, USA) were loaded as molecular weight marker. Three different double strand DNA fragments were radio-labelled with [α-32]-CTP using the Amersham Rediprime II DNA labelling System (GE Healthcare Europe, GmbH, Milan, Italy) and used as probes. The 5′ probe (875 bp) was generated by digestion of the pZac2.1-CMV-*ABCA4_5′* plasmid with EagI; the 3′ probe (880 bp) was generated by double digestion of the pZac2.1-*ABCA4_*3′ *_3xflag_*SV40 plasmid with StuI and NcoI; the EGFP probe (735 bp) was generated by digestion of the pAAV2.1-CMV-EGFP plasmid with NotI and BamHI. Prehybridization and hybridization were performed at 65°C in Church buffer (Sambrook & Russell, 2001) for 1 h and overnight, respectively. Then, the membrane (Whatman Nytran N, charged nylon membrane; Sigma-Aldrich, Milan, Italy) was washed for 30 min in SSC 2×-0.1% SDS and for 30 min in SSC 0.5×-0.1% SDS at 65°C, and then for 30 min in SSC 0.1×-0.1% SDS at 37°C. The membrane was then analyzed by X-ray autoradiography using Amersham Hyperfilm™ MP (GE Healthcare Europe, GmbH).

### AAV infection of HEK293 cells

HEK293 cells were maintained in Dulbecco's modified Eagle's medium (DMEM) containing 10% fetal bovine serum and 2 mM l-glutamine (GIBCO; Invitrogen S.R.L.). Cells were plated in six-well plates at a density of 2 × 10^6^ cells/well and transfected 16 h later with 1.3 μg of pDeltaF6 helper plasmid which contains the Ad helper genes (Zhang *et al*, 2000) using the calcium phosphate method. After 5 h, cells were washed once with serum-free DMEM and incubated with AAV2/2 vectors (m.o.i: 5 × 10^4^ GC/cell of each vector; 1:1 co-infection with dual AAV vectors resulted in 1 × 10^5^ total GC/cell) in a final volume of 700 μl serum-free DMEM. Two hours later 2 ml of complete DMEM were added to the cells. Cells were harvested 72 h following infection for Western blot analysis.

### Animal models

Mice were housed at the Institute of Genetics and Biophysics animal house (Naples, Italy) and maintained under a 12-h light/dark cycle (10–50 lux exposure during the light phase). C57BL/6 and BALB/c mice were purchased from Harlan Italy SRL (Udine, Italy). Albino *Abca4*^−/−^ mice were generated through successive crosses and backcrosses with BALB/c mice (homozygous for Rpe65 Leu450; Radu *et al*, 2004) and maintained inbred. Breeding was performed crossing homozygous mice. Pigmented *shaker1 4626SB/4626SB* (referred to as *sh1*^−/−^) mice were imported from the Wellcome Trust Sanger Institute (Cambridge, UK, a kind gift of Dr Karen Steel) and back-crossed twice with CBA/Ca mice purchased from Harlan Italy SRL to obtain heterozygous *shaker1*^+^/ *4626SB* (referred to as *sh1*^+/–^) mice to expand the colony. The mice were maintained intercrossed; breeding was performed crossing heterozygous females with heterozygous males. The pigmented *sh1* mice used in this study were either affected ( *sh1*^−/−^) or unaffected ( *sh1*^+/–^ and *sh1*^+/+^). Albino *shaker1 4626SB/4626SB* mice (referred as *sh1*^−/−^) were imported from the Medical Research Council Institute of Hearing Research (Nottingham, UK) and maintained inbred; breedings were performed crossing heterozygous female with homozygous males. The albino *sh1* mice used in this study were either affected ( *sh1*^−/−^) or unaffected ( *sh1*^+/–^). Figure 7B and supplementary Figs S13 and S14 show data from albino *sh1*^−/−^ mice. The genotype for the *MYO7A*^*4626SB*^ allele was performed by PCR analysis of genomic DNA (extracted from the mouse tail tip) followed by DNA sequencing. The primers used for the PCR amplification are as follows: Fw1 (GTGGAGCTTGACATCTACTTGACC) and Rev3 (AGCTGACCCTCATGACTCTGC), which generate a product of 712 bp that was sequenced with the Fw1 primer. The Large White Female pigs used in this study were registered as purebred in the LWHerd Book of the Italian National Pig Breeders' Association (Azienda Agricola Pasotti, Imola, Italy).

### Subretinal injection of AAV vectors in mice and pigs

This study was carried out in accordance with the Association for Research in Vision and Ophthalmology Statement for the Use of Animals in Ophthalmic and Vision Research and with the Italian Ministry of Health regulation for animal procedures. All procedures on mice were submitted to the Italian Ministry of Health; Department of Public Health, Animal Health, Nutrition and Food Safety on October 17th, 2011. The Ministry of Health approved the procedures by silence/consent, as per article 7 of the 116/92 Ministerial Decree. Surgery was performed under anesthesia and all efforts were made to minimize suffering.

Mice (4–5 week-old) were anesthetized with an intraperitoneal injection of 2 ml/100 g body weight of avertin [1.25% w/v of 2,2,2-tribromoethanol and 2.5% v/v of 2-methyl-2-butanol (Sigma-Aldrich)] (Papaioannou & Fox, 1993), then AAV2/8 vectors were delivered subretinally via a trans-scleral trans-choroidal approach as described by Liang *et al* (2000). All eyes were treated with 1 μl of vector solution. The AAV2/8 doses (GC/eye) delivered vary across the different mouse experiments as it is described in the Results section. To compare dual AAV to single AAV NS vectors (supplementary Fig S1 and Fig 4A) we used the same dose of each vector because we considered that one GC of the 5′-vector plus one GC of the 3′-vector of dual AAVs are required to re-constitute one full-size functional genome as that contained in one particle of AAV NS. To exclude competition between dual AAV capsids at the entry step which may lead us to over-estimate the efficiency of AAV NS, we evaluated EGFP expression after subretinal delivery of either 1.7 × 10^9^ GC of AAV2/8 NS-EGFP or 1.7 × 10^9^ GC of AAV2/8-NS-EGFP + 1.7 × 10^9^ GC of an AAV2/8 vector carrying an unrelated genome (AAV-unrelated, supplementary Fig S16). Notably, we found no significant differences in the levels of EGFP expression whether the unrelated AAV was added or not (supplementary Fig S16), proving that the double dose of dual AAV capsids administered when compared to AAV-NS does not affect dual AAV-mediated transduction.

AAV2/1-VMD2-*human Tyrosinase* (Gargiulo *et al*, 2009; dose: 2–5 × 10^8^ GC/eye) or AAV2/5-CMV-*EGFP* (encoding normal size EGFP, dose: 4 × 10^8^ GC/eye) were added to the AAV2/8 vector solution that was subretinally delivered to albino mice ( *Abca4*^−/−^, BALB/c, and *sh1*; Figs 5B, C and D and 6A and B; supplementary Figs S10 and S14) or pigmented *sh1* mice (Fig 8), respectively. This allowed us to mark the RPE within the transduced part of the eyecup, which was subsequently dissected and analyzed. Subretinal delivery of AAV vectors to the pig retina was performed as previously described (Mussolino *et al*, 2011). All eyes were treated with 100 μl of AAV2/8 vector solution. The AAV2/8 dose was 1 × 10^10^ (Fig 3B) or 1 × 10^11^ GC of each vector/eye (Fig 4B) and co-injection of dual AAV vectors resulted in a total dose of 2 × 10^10^ GC/eye or 2 × 10^11^ GC/eye, respectively.

### Western blot analysis and ELISA

Samples [HEK293 cells, retinas or eyecups (cups + retinas)] for Western blot analysis were lysed in RIPA buffer (50 mM Tris–HCl pH 8.0, 150 mM NaCl, 1% NP40, 0.5% Na-Deoxycholate, 1 mM EDTA pH 8.0, 0.1% SDS) to extract EGFP and MYO7A proteins from HEK293 cells and eyecups, or in SIE buffer (250 mM sucrose, 3 mM imidazole pH 7.4, 1% ethanol, and 1% NP-40) to extract MYO7A from retinas and ABCA4 protein. Lysis buffers were supplemented with protease inhibitors (Complete Protease inhibitor cocktail tablets; Roche) and 1 mM phenylmethylsulfonyl. After lysis EGFP and MYO7A samples were denatured at 99°C for 5 min in 1X Laemli sample buffer; ABCA4 samples were denatured at 37°C for 15 min in 1X Laemli sample buffer supplemented with 4 M urea. Lysates were separated by 7% (ABCA4 and MYO7A samples) or 12% (EGFP samples) SDS–polyacrylamide gel electrophoresis. The antibodies used for immuno-blotting are as follows: anti-EGFP (1:500, sc-8334; Santa Cruz, Dallas, TX, USA); anti-3xflag (1:1000, A8592; Sigma-Aldrich); anti-Myo7a (1:500, polyclonal; Primm Srl, Milan, Italy) generated using a peptide corresponding to aminoacids 941–1070 of the human MYO7A protein; anti-HA antibody (1:2000, PRB-101P-200, HA.11; Covance, Princeton, NJ, USA); anti-β Tubulin (1:10 000, T5201; Sigma Aldrich); anti-Filamin A (1:1000, catalog#4762; Cell Signaling Technology, Danvers, MA, USA); anti-Dysferlin (1:500, Dysferlin, clone Ham1/7B6, MONX10795; Tebu-bio, Le Perray-en-Yveline, France). The quantification of EGFP, ABCA4 and MYO7A bands detected by Western blot was performed using ImageJ software (free download is available at http://rsbweb.nih.gov/ij/). ABCA4 and MYO7A expression was normalized to Filamin A or Dysferlin for the *in vitro* and *in vivo* experiments, respectively. EGFP expression was normalized to β-Tubulin. Different proteins were used for normalization based on the similarity of their molecular weight to those of the different transgene products. The TS (Fig 2D and E), TS-L (Fig 2F) and NS (supplementary Fig S1) histogram do not have standard error bars as only one TS, TS-L or NS sample has been loaded on each SDS–PAGE and used as internal reference sample in each independent experiment. To show the internal variability of TS, TS-L and NS samples we calculated the expression of proteins as percentage relative to the AK sample (set to 100%) which are the following: Fig 2D: TS = 106 ± 16%; Fig 2E: TS = 78 ± 4%; Fig 2F: TS-L = 39 ± 6%; supplementary Fig S1: NS = 906 ± 281%. The ELISA was performed on retina or eyecup lysates using the Max Discovery Green Fluorescent Protein Kit ELISA (Bioo Scientific Corporation, Austin, TX, USA).

### Fundus photography

The fundus live-imaging was performed by dilating the eye of C57BL/6 with a drop of tropicamide 1% (Visufarma, Rome, Italy) and subsequent eye stimulation with a 300W flash. Fundus photographs were taken using a Topcon TRC-50IX retinal camera, with a FITC filter, connected to a charge-coupled-device Nikon D1H digital camera (Topcon Medical System, Oakland, NJ, USA).

### Histology, light and fluorescence microscopy

To evaluate EGFP expression in histological sections, eyes from C57BL/6 mice or Large White pigs (Mussolino *et al*, 2011) were enucleated 1 month after AAV2/8 injection. Mouse eyes were fixed in 4% paraformaldehyde over-night and infiltrated with 30% sucrose over-night; the cornea and the lens were then dissected and the eyecups were embedded in optimal cutting temperature compound (O.C.T. matrix; Kaltek, Padua, Italy). PR co-transduction following subretinal combined delivery of AAV2/8-CMV-*EGFP* and-*RFP* vectors has been evaluated as follows: retinal cryosections from *n* = 6 eyes were analyzed under a fluorescent microscope using either the FITC (to visualize EGFP^+^ cells) or Rhodamine (to visualize RFP^+^ cells) filters. For each eye RFP^+^ PR contained in one field at 20× magnification (at least 100) and the corresponding EGFP^+^ PR (at least 200) were photographed and then counted. PR expressing both EGFP and RFP were unequivocally identified based on their identical shape on picture micrographs of the same field taken under either the FITC or Rhodamine filter. To calculate the percentage of co-transduced PR, the number of PR expressing both EGFP and RFP was divided by the total number of transduced PR, i.e. PR expressing at least one of the two reporter genes.

Pig eyes were fixed in 4% paraformaldehyde for 48 h, infiltrated with 10% sucrose for 4 h, 20% sucrose for 4 h and finally 30% sucrose overnight. Then, the cornea, the lens, and the vitreous body were dissected and the EGFP-positive portions of the eyecups were embedded in optimal cutting temperature compound (O.C.T. matrix; Kaltek). Serial cryosections (12 μm thick) were cut along the horizontal meridian and progressively distributed on slides. Retinal histology pictures were captured using a Zeiss Axiocam (Carl Zeiss, Oberkochen, Germany). Subretinal delivery in pigs of AAV vectors encoding for EGFP under the control of the Rhodopsin promoter resulted in PR transduction in: 100% of the retinas injected with: dual AAV TS (4/4), and dual AAV hybrid AK (3/3, Fig 4B).

To analyze melanosome localization in the RPE of pigmented *sh1* mice, eyes were enucleated 2–3 months following the AAV injection, fixed in 2% glutaraldehyde-2% paraformaldehyde in 0.1 M phosphate buffer over-night, rinsed in 0.1 M phosphate buffer and dissected under a florescence microscope. The EGFP-positive portions of the eyecups were embedded in Araldite 502/EMbed 812 (catalog #13940, Araldite 502/EMbed 812 KIT; Electron Microscopy Sciences, Hatfield, PA, USA). Semi-thin (0.5 μm) sections were transversally cut on a Leica Ultratome RM2235 (Leica Microsystems, Bannockburn, IL, USA), mounted on slides and stained with Epoxy tissue stain (catalog #14950; Electron Microscopy Sciences). Melanosomes were counted by a masked operator in about 10 different fields/eye under a light microscope at 100× magnification. Retinal pictures were captured using a Zeiss Axiocam (Carl Zeiss).

### Flow cytometry

Flow cytometry analysis was carried out as described (Palfi *et al*, 2012). Briefly, eyes in adult 129 mice were subretinally injected with 3 μl of a 1:1 mixture of 3 × 10^9^ GC of AAV2/8-EGFP and 3 × 10^9^ GC of AAV2/8-DsRed. Control retinas were injected with 3 μl of either 3 × 10^9^ GC of AAV2/8-EGFP or 3 μl of 3 × 10^9^ GC of AAV2/8-DsRed or were left un-injected. Three weeks post-injection, retinas were dissociated in trypsin/HBSS, stained with DRAQ5 (Biostatus, Shepshed, UK) and EGFP (488:530/40), DsRed (564:615/20) and DRAQ5 (633: 665/20) fluorescence signals analyzed in the live cells using a Beckman Coulter Cyan ADP flow cytometer (Beckman Coulter Diagnostics Limited, Lismeehan, Ireland) modified by Propel Labs (Fort Collins, CO, USA). The analysis was carried out using Summit v4.3 (Beckman Coulter) software. Nucleated events were selected by gating the brightest DRAQ5 events in a bivariate density plot of the forward light scatter (FSC) versus DRAQ5. A second selection was done in the bivariate histogram of forward versus side scatter (FSC versus SSC), selecting the homogeneous and perfectly well defined population of events. Between 10 000 and 25 000 events were analyzed per sample.

### Electron microscopy and immuno-gold labelling

For electron microscopy analyzes eyes were harvested from *Abca4*^−/−^ or *sh1* mice at 3 and 2–3 months after AAV injection, respectively. Eyes were fixed in 0.2% glutaraldehyde-2% paraformaldehyde in 0.1 M PHEM buffer pH 6.9 (240 mM PIPES, 100 mM HEPES, 8 mM MgCl_2_, 40 mM EGTA) for 2 h and then rinsed in 0.1 M PHEM buffer. Eyes were then dissected under light or fluorescence microscope to select the Tyrosinase-or EGFP-positive portions of the eyecups of albino ( *Abca4*^−/−^, BALB/c and *sh1*^−/−^) and pigmented *sh1* mice *,* respectively. The transduced portion of the eyecups were subsequently embedded in 12% gelatin, infused with 2.3 M sucrose and frozen in liquid nitrogen. Cryosections (50 nm) were cut using a Leica Ultramicrotome EM FC7 (Leica Microsystems) and extreme care was taken to align PR connecting cilia longitudinally. To avoid bias in the attribution of morphological data to the various experimental groups, measurements of RPE thickness and counts of lipofuscin granules in *Abca4*^−/−^ eyes were performed by a masked operator (Roman Polishchuk) using the iTEM software (Olympus SYS, Hamburg, Germany). Briefly, RPE thickness was measured in at least 30 different areas along the specimen length using the ‘Arbitrary Line’ tool of iTEM software. The ‘Touch count’ module of the iTEM software was utilized to count the number of lipofuscin granules in the 25 μm^2^ areas distributed randomly across the RPE layer. The granule density was expressed as number of granules per 25 μm^2^. The immuno-gold analysis aimed at testing the expression of ABCA4-HA in *Abca4*^−/−^ samples after AAV vector delivery was performed by incubating cryosections successively with monoclonal anti-HA antibody (MMS-101P-50; Covance, 1:50), rabbit anti-mouse IgG, and 10-nm gold particle-conjugated protein A. To quantify rhodopsin localization to the connecting cilium of *sh1* PR, cryosections of *sh1* mice were successively incubated with anti-rhodopsin antibody (1D4, ab5417; Abcam, Cambridge, UK, 1:100), rabbit anti-mouse IgG, and 10-nm gold particle-conjugated protein A. The quantification of gold density of rhodospin in the connecting cilia was performed by a masked operator using iTEM software (Olympus SYS). Briefly, the ‘Touch count’ module of the iTEM software was used to count the number of gold particles per cilium that were then normalized to the cilium perimeter (nm) measured using the ‘Closed polygon tool’. Gold density was expressed as gold particles/μm. Immunogold labelled cryosections were analyzed under FEI Tecnai-12 (FEI, Eindhoven, The Netherlands) electron microscope equipped with a Veletta CCD camera (Soft Imaging Systems, Munster, Germany) for digital image acquisition.

### A2E measurement in *Abca4^−/−^* mice

To further evaluate lipofuscin accumulation in *Abca4*^−/−^ mice, we attempted at measuring by either HPLC in combination with mass spectrometry (Gutierrez *et al*, 2010) or by HPLC alone (Parish *et al*, 1998; Ben-Shabat *et al*, 2002; Allocca *et al*, 2008) the A2E content of *Abca4*^−/−^ eyecups which is reported to be increased compared to wild-type controls (Weng *et al*, 1999; Radu *et al*, 2004; Allocca *et al*, 2008).

### Electrophysiological analyzes

To assess the recovery from light desensitization eyes were stimulated with three light flashes of 1 cd s/m^2^ and then desensitized by exposure to constant light (300 cd/m^2^) for 3 min. Then, eyes were stimulated over time using the pre-desensitization flash (1 cd s/m^2^) at 0, 5, 15, 30, 45 and 60 min post-desensitization. The recovery of rod activity was evaluated by performing the ratio between the b-wave generated post-desensitization (at the different time points) and that generated pre-desensitization.

### RNA extraction, cDNA production and reverse transcription analyzes

RNA was extracted at 72 h after HEK293 cells infection with dual AAV2/2 TS and hybrid AK vectors encoding for either *ABCA4* or *MYO7A* (5′+3′halves) and as negative controls with either the 5′or 3′half of dual AAV2/2 vectors or with a single NS AAV2/2 *EGFP* vector.

Total RNA was extracted using the RT–PCR RNeasy MiniKit (Qiagen, Milan, Italy). One microgram of RNA was submitted to DNase I digestion (RNase Free DNase set; Qiagen) and cDNA was generated using the QuantiTect reverse transcription kit (Qiagen). To amplify the *ABCA4* mRNA region corresponding to the exon-intron junction used in dual AAV TS and hybrid AK vectors, 1 μl of cDNA and the following primers were used: Abca4_RT_Fw 5′-GCTGGGAAAACCACCACC-3′ and Abca4_RT_Rev 5′-GTGGACACATGCCAAGGC-3′. A PCR product of the expected size (130 bp) was then sequenced. Five microlitres of cDNA were insted used to amplify the full-length *ABCA4* mRNA (6.9 kb) with a long-range PCR using TaKaRa LA Taq DNA polymerase kit (TaKaRa, Kioto, Japan), and the following primers: ATGFw 5′-GGTACCTCTAGAGTCGACCCGG-3′, which anneals upstream of the ATG start codon and SV40polyA-Rev 5′-ACTCATCAATGTATCTTATCATGTCTG-3′. To amplify the MYO7A mRNA region corresponding to the exon-intron junction used in dual AAV TS and hybrid AK vectors, 1.5 μl of cDNA and the following primers were used: Fw 5′-AGGGGACAACTACGCACTC-3′ Rev 5′-GTCTTCTTGCCCAGGGTCTC-3′. A PCR product of the expected size (218 bp) was then sequenced. Two micrograms of total RNA were instead retro-transcribed using SuperScript® III First-Strand Synthesis System (Invitrogen) and 1 μl of cDNA was used to amplify the full-length *MYO7A* mRNA (6.7 kb) with a long-range PCR using TaKaRa LA Taq DNA polymerase kit (TaKaRa) and the following primers: ATGFw 5′-GCGGCCGCCATGGTGATTCTTCAGCAG-3′ and BgHpolyA-Rev 5′-TGGGAGTGGCACCTTCCA-3′.

### Statistical analysis

Statistical *P* values ≤ 0.05 were considered significant. One-way ANOVA with *post-hoc* Multiple Comparison Procedure was used to compare data depicted in: Fig 2 ( *P* ANOVA = A. 0.012; B. 1 × 10^–7^; C. 0.002); Fig 4A ( *P* ANOVA = 1.9 × 10^–12^); Fig 6B ( *P* ANOVA = 0.00126); Fig 6C ( *P* ANOVA = 2.2 × 10^–5^); Fig 8B ( *P* ANOVA = 1.2 × 10^–5^); Fig 8C ( *P* ANOVA = 0.11); supplementary Fig S1 ( *P* ANOVA = 2.3 × 10^–8^); supplementary Fig S6 ( *P* ANOVA = A: 0.13; B: 0.16); supplementary Table S2 (2/2 preps: *P* ANOVA = 0.0698; 2/8 preps: *P* ANOVA = 0.0767). As the counts of lipofuscin granules (Fig 5D) are expressed as discrete numbers, these were analyzed by deviance from a Negative Binomial generalized linear models (Venables & Ripley, 2002) ( *P* value analysis of deviance: Fig 5D: 1.7 × 10^–7^). The statistically significant differences between groups determined with the *post-hoc* Multiple Comparison Procedure are the following: Fig 2D: OV versus OZ *P* = 0.03; OV versus AP *P* = 0.016. Fig 2E: OV versus OZ *P* = 0.0001; OV versus AP *P* = 1.1 × 10^–5^; OV versus AK *P* = 0.0017; TS versus OZ *P* = 1.8 × 10^–5^; TS versus AP *P* = 2.3 × 10^–6^; TS versus AK *P* = 0.026; AK versus OZ *P* = 9 × 10^–7^; AK versus AP *P* = 2 × 10^–7^. Fig 2F: AK-L versus OZ *P* = 0.003; AK-L versus TS-L *P* = 0.005. Fig 4A: NS-*EGFP* versus TS-*EGFP P* = 0; NS-*EGFP* versus AK-*EGFP P* = 0. Fig 5D: WT versus *Abca4*^−/−^ neg *P* = 0.016; WT versus *Abca4*^−/−^ AK-*ABCA4 P* = 0.03; *Abca4*^−/−^ neg versus *Abca4*^−/−^ TS-*ABCA4 P* = 0.0005; *Abca4*^−/−^ neg versus *Abca4*^−/−^ AK-*ABCA4 P* = 9 × 10^–8^. Fig 6B: *Abca4*^−/−^ neg versus *Abca4*^−/−^ TS-*ABCA4 P* = 0.012; *Abca4*^−/−^ neg versus *Abca4*^−/−^ AK-*ABCA4 P* = 0.002. Fig 6C (60 min): *Abca4*^−/−^ neg versus *Abca4*^−/−^ AK-*ABCA4*: 0.05; *Abca4*^−/−^ neg versus *Abca4*^−/−^ TS-*ABCA4*: 0.009; *Abca4*^−/−^ AK-*ABCA4* versus WT: 0.002; *Abca4*^−/−^ TS-*ABCA4* versus WT: 0.02 *Abca4*^−/−^ neg versus WT 1 × 10^–5^. Fig 8B: *sh1*^+/+^ and *sh1*^+/–^ versus *sh1*^−/−^ neg *P* = 7.7 × 10^–6^; *sh1*^+/+^ and *sh1*^+/–^ versus *sh1*^−/−^ TS-*MYO7A P* = 0.025; *sh1*^−/−^ neg versus *sh1*^−/−^ TS-*MYO7A P* = 0.0028; *sh1*^−/−^ neg versus *sh1*^−/−^ AK-*MYO7A P* = 0.0002. Supplementary Fig S1: NS-*EGFP* versus TS-*EGFP P* = 0; NS-*EGFP* versus AK-*EGFP P* = 1 × 10^–7^.

The Student's *t*-test was used to compare data depicted in supplementary Fig S10, S14 and S16. The data in the manuscript are depicted as mean ± standard error of the mean (s.e.m.). The s.e.m has been calculated using the number of independent *in vitro* experiments or eyes (not replicate measurements of the same sample).

## For more information

Stargardt's disease: http://www.macular.org/stargardts.htm Online Mendelian Inheritance in Man (OMIM): http://www.ncbi.nlm.nih.gov/omim Retinal Information Network (RetNet): https://sph.uth.tmc.edu/retnet/ Author's website: http://www.tigem.it/en/research/researchers/alberto-auricchio

The paper explained Problem Inherited retinal degenerations (IRDs) are a common cause of blindness worldwide. Gene therapy with AAV vectors is safe and effective in patients with a childhood form of IRD. One of the major limitations to extend this clinical success to other blinding conditions is that many are caused by mutations in genes with large coding sequences that exceed AAV DNA cargo capacity. Results We have generated dual AAV vectors each containing one half of a large transgene which is reconstituted upon target cell co-infection by either splicing (trans-splicing, TS), homologous recombination (overlapping, OV) or a combination of the two (hybrid). We show that the delivery of dual AAV TS and hybrid vectors to the retina results in efficient reconstitution of large transgenes in mouse and pig photoreceptors, the major cell target of IRDs. Although the levels of transgene expression achieved with dual AAV vectors are lower than those achieved with a single normal size AAV, they result in significant improvement of the retinal disease of two mouse models of common IRDs due to mutations in large genes, Stargardt's disease (STGD) and Usher syndrome type IB (USH1B). Impact Our results provide proof-of-concept of gene therapy for STGD and USH1B using dual AAV vectors. Importantly, dual AAV vectors expand AAV cargo capacity for gene therapy of IRDs requiring transfer of large genes.

## References

[b1] Allikmets R (1997). A photoreceptor cell-specific ATP-binding transporter gene (ABCR) is mutated in recessive Stargardt macular dystrophy. Nat Genet.

[b2] Allocca M, Doria M, Petrillo M, Colella P, Garcia-Hoyos M, Gibbs D, Kim SR, Maguire A, Rex TS, Di Vicino U (2008). Serotype-dependent packaging of large genes in adeno-associated viral vectors results in effective gene delivery in mice. J Clin Invest.

[b3] Allocca M, Mussolino C, Garcia-Hoyos M, Sanges D, Iodice C, Petrillo M, Vandenberghe LH, Wilson JM, Marigo V, Surace EM (2007). Novel adeno-associated virus serotypes efficiently transduce murine photoreceptors. J Virol.

[b4] Auricchio A (2011). Fighting blindness with adeno-associated virus serotype 8. Hum Gene Ther.

[b5] Auricchio A, Hildinger M, O'Connor E, Gao GP, Wilson JM (2001). Isolation of highly infectious and pure adeno-associated virus type 2 vectors with a single-step gravity-flow column. Hum Gene Ther.

[b6] Bainbridge JW, Smith AJ, Barker SS, Robbie S, Henderson R, Balaggan K, Viswanathan A, Holder GE, Stockman A, Tyler N (2008). Effect of gene therapy on visual function in Leber's congenital amaurosis. N Engl J Med.

[b7] Ben-Shabat S, Parish CA, Vollmer HR, Itagaki Y, Fishkin N, Nakanishi K, Sparrow JR (2002). Biosynthetic studies of A2E, a major fluorophore of retinal pigment epithelial lipofuscin. J Biol Chem.

[b8] Campbell RE, Tour O, Palmer AE, Steinbach PA, Baird GS, Zacharias DA, Tsien RY (2002). A monomeric red fluorescent protein. Proc Natl Acad Sci USA.

[b9] Cideciyan AV, Hauswirth WW, Aleman TS, Kaushal S, Schwartz SB, Boye SL, Windsor EA, Conlon TJ, Sumaroka A, Roman AJ (2009). Vision 1 year after gene therapy for Leber's congenital amaurosis. N Engl J Med.

[b10] Colella P, Cotugno G, Auricchio A (2009). Ocular gene therapy: current progress and future prospects. Trends Mol Med.

[b11] Conley SM, Cai X, Makkia R, Wu Y, Sparrow JR, Naash MI (2012). Increased cone sensitivity to ABCA4 deficiency provides insight into macular vision loss in Stargardt's dystrophy. Biochim Biophys Acta.

[b12] Dong B, Nakai H, Xiao W (2010a). Characterization of genome integrity for oversized recombinant AAV vector. Mol Ther.

[b13] Dong X, Tian W, Wang G, Dong Z, Shen W, Zheng G, Wu X, Xue J, Wang Y, Chen J (2010b). Establishment of an AAV reverse infection-based array. PLoS One.

[b101] Doria M, Ferrara A, Auricchio A (2013). AAV2/8 vectors purified from culture medium with a simple and rapid protocol transduce murine liver, muscle, and retina efficiently. Hum Gene Ther Methods.

[b14] Drittanti L, Rivet C, Manceau P, Danos O, Vega M (2000). High throughput production, screening and analysis of adeno-associated viral vectors. Gene Ther.

[b15] Dryja T, Scriver C, Beaudet A, Sly W, Valle D (2001). Retinitis pigmentosa and stationary night blindness. The Online Metabolic and Molecular Bases of Inherited Diseases.

[b16] Duan D, Sharma P, Yang J, Yue Y, Dudus L, Zhang Y, Fisher KJ, Engelhardt JF (1998). Circular intermediates of recombinant adeno-associated virus have defined structural characteristics responsible for long-term episomal persistence in muscle tissue. J Virol.

[b17] Duan D, Yue Y, Engelhardt JF (2001). Expanding AAV packaging capacity with trans-splicing or overlapping vectors: a quantitative comparison. Mol Ther.

[b18] Esumi N, Oshima Y, Li Y, Campochiaro PA, Zack DJ (2004). Analysis of the VMD2 promoter and implication of E-box binding factors in its regulation. J Biol Chem.

[b19] Fishel ML, Vasko MR, Kelley MR (2007). DNA repair in neurons: so if they don't divide what's to repair?. Mutat Res.

[b20] Flotte TR, Afione SA, Solow R, Drumm ML, Markakis D, Guggino WB, Zeitlin PL, Carter BJ (1993). Expression of the cystic fibrosis transmembrane conductance regulator from a novel adeno-associated virus promoter. J Biol Chem.

[b21] Gao G, Qu G, Burnham MS, Huang J, Chirmule N, Joshi B, Yu QC, Marsh JA, Conceicao CM, Wilson JM (2000). Purification of recombinant adeno-associated virus vectors by column chromatography and its performance in vivo. Hum Gene Ther.

[b22] Gargiulo A, Bonetti C, Montefusco S, Neglia S, Di Vicino U, Marrocco E, Corte MD, Domenici L, Auricchio A, Surace EM (2009). AAV-mediated tyrosinase gene transfer restores melanogenesis and retinal function in a model of oculo-cutaneous albinism type I (OCA1). Mol Ther.

[b23] Ghosh A, Yue Y, Duan D (2006). Viral serotype and the transgene sequence influence overlapping adeno-associated viral (AAV) vector-mediated gene transfer in skeletal muscle. J Gene Med.

[b24] Ghosh A, Yue Y, Duan D (2011). Efficient transgene reconstitution with hybrid dual AAV vectors carrying the minimized bridging sequences. Hum Gene Ther.

[b25] Ghosh A, Yue Y, Lai Y, Duan D (2008). A hybrid vector system expands adeno-associated viral vector packaging capacity in a transgene-independent manner. Mol Ther.

[b26] Gibbs D, Diemer T, Khanobdee K, Hu J, Bok D, Williams DS (2010). Function of MYO7A in the human RPE and the validity of shaker1 mice as a model for Usher syndrome 1B. Invest Ophthalmol Vis Sci.

[b27] Grieger JC, Samulski RJ (2005). Packaging capacity of adeno-associated virus serotypes: impact of larger genomes on infectivity and postentry steps. J Virol.

[b28] Grose WE, Clark KR, Griffin D, Malik V, Shontz KM, Montgomery CL, Lewis S, Brown RH, Janssen PM, Mendell JR (2012). Homologous recombination mediates functional recovery of dysferlin deficiency following AAV5 gene transfer. PLoS One.

[b29] Gutierrez DB, Blakeley L, Goletz PW, Schey KL, Hanneken A, Koutalos Y, Crouch RK, Ablonczy Z (2010). Mass spectrometry provides accurate and sensitive quantitation of A2E. Photochem Photobiol Sci.

[b30] Han Z, Conley SM, Makkia RS, Cooper MJ, Naash MI (2012). DNA nanoparticle-mediated ABCA4 delivery rescues Stargardt dystrophy in mice. J Clin Invest.

[b31] Hasson T, Heintzelman MB, Santos-Sacchi J, Corey DP, Mooseker MS (1995). Expression in cochlea and retina of myosin VIIa, the gene product defective in Usher syndrome type 1B. Proc Natl Acad Sci USA.

[b32] Hermonat PL, Quirk JG, Bishop BM, Han L (1997). The packaging capacity of adeno-associated virus (AAV) and the potential for wild-type-plus AAV gene therapy vectors. FEBS Lett.

[b33] Hirsch ML, Agbandje-McKenna M, Samulski RJ (2010). Little vector, big gene transduction: fragmented genome reassembly of adeno-associated virus. Mol Ther.

[b34] Hirsch ML, Li C, Bellon I, Yin C, Chavala S, Pryadkina M, Richard I, Samulski RJ (2013). Oversized AAV transduction is mediated via a DNA-PKcs Independent, Rad51C-dependent repair pathway. Mol Ther.

[b35] Hirsch ML, Storici F, Li C, Choi VW, Samulski RJ (2009). AAV recombineering with single strand oligonucleotides. PLoS One.

[b36] Illing M, Molday LL, Molday RS (1997). The 220-kDa rim protein of retinal rod outer segments is a member of the ABC transporter superfamily. J Biol Chem.

[b37] Jacobson SG, Acland GM, Aguirre GD, Aleman TS, Schwartz SB, Cideciyan AV, Zeiss CJ, Komaromy AM, Kaushal S, Roman AJ (2006). Safety of recombinant adeno-associated virus type 2-RPE65 vector delivered by ocular subretinal injection. Mol Ther.

[b38] Lai Y, Yue Y, Duan D (2010). Evidence for the failure of adeno-associated virus serotype 5 to package a viral genome ≥ 8.2 kb. Mol Ther.

[b39] Lai Y, Yue Y, Liu M, Ghosh A, Engelhardt JF, Chamberlain JS, Duan D (2005). Efficient in vivo gene expression by trans-splicing adeno-associated viral vectors. Nat Biotechnol.

[b40] Liang FQ, Anand V, Maguire AM, Bennett J, Rakoczy PE (2000). Intraocular delivery of recombinant virus. Methods in Molecular Medicine: Vision Research Protocols.

[b41] Lillo C, Kitamoto J, Liu X, Quint E, Steel KP, Williams DS (2003). Mouse models for Usher syndrome 1B. Adv Exp Med Biol.

[b42] Liu X, Ondek B, Williams DS (1998). Mutant myosin VIIa causes defective melanosome distribution in the RPE of shaker-1 mice. Nat Genet.

[b43] Liu X, Udovichenko IP, Brown SD, Steel KP, Williams DS (1999). Myosin VIIa participates in opsin transport through the photoreceptor cilium. J Neurosci.

[b44] Liu X, Vansant G, Udovichenko IP, Wolfrum U, Williams DS (1997). Myosin VIIa, the product of the Usher 1B syndrome gene, is concentrated in the connecting cilia of photoreceptor cells. Cell Motil Cytoskeleton.

[b45] Lopes VS, Boye SE, Louie CM, Boye S, Dyka F, Chiodo V, Fofo H, Hauswirth WW, Williams DS (2013). Retinal gene therapy with a large MYO7A cDNA using adeno-associated virus. Gene Ther.

[b46] Ma L, Kaufman Y, Zhang J, Washington I (2011). C20-D3-vitamin A slows lipofuscin accumulation and electrophysiological retinal degeneration in a mouse model of Stargardt disease. J Biol Chem.

[b47] Maguire AM, High KA, Auricchio A, Wright JF, Pierce EA, Testa F, Mingozzi F, Bennicelli JL, Ying GS, Rossi S (2009). Age-dependent effects of RPE65 gene therapy for Leber's congenital amaurosis: a phase 1 dose-escalation trial. Lancet.

[b48] Maguire AM, Simonelli F, Pierce EA, Pugh EN, Mingozzi F, Bennicelli J, Banfi S, Marshall KA, Testa F, Surace EM (2008). Safety and efficacy of gene transfer for Leber's congenital amaurosis. N Engl J Med.

[b49] Maiti P, Kong J, Kim SR, Sparrow JR, Allikmets R, Rando RR (2006). Small molecule RPE65 antagonists limit the visual cycle and prevent lipofuscin formation. Biochemistry (Mosc).

[b50] Mata NL, Tzekov RT, Liu X, Weng J, Birch DG, Travis GH (2001). Delayed dark-adaptation and lipofuscin accumulation in abcr+/-mice: implications for involvement of ABCR in age-related macular degeneration. Invest Ophthalmol Vis Sci.

[b51] Millan JM, Aller E, Jaijo T, Blanco-Kelly F, Gimenez-Pardo A, Ayuso C (2011). An update on the genetics of usher syndrome. J Ophthalmol.

[b52] Molday RS, Zhang K (2010). Defective lipid transport and biosynthesis in recessive and dominant Stargardt macular degeneration. Prog Lipid Res.

[b53] Monahan PE, Lothrop CD, Sun J, Hirsch ML, Kafri T, Kantor B, Sarkar R, Tillson DM, Elia JR, Samulski RJ (2010). Proteasome inhibitors enhance gene delivery by AAV virus vectors expressing large genomes in hemophilia mouse and dog models: a strategy for broad clinical application. Mol Ther.

[b55] Mussolino C, della Corte M, Rossi S, Viola F, Di Vicino U, Marrocco E, Neglia S, Doria M, Testa F, Giovannoni R (2011). AAV-mediated photoreceptor transduction of the pig cone-enriched retina. Gene Ther.

[b56] Natkunarajah M, Trittibach P, McIntosh J, Duran Y, Barker SE, Smith AJ, Nathwani AC, Ali RR (2008). Assessment of ocular transduction using single-stranded and self-complementary recombinant adeno-associated virus serotype 2/8. Gene Ther.

[b57] Palfi A, Chadderton N, McKee AG, Blanco Fernandez A, Humphries P, Kenna PF, Farrar GJ (2012). Efficacy of codelivery of dual AAV2/5 vectors in the murine retina and hippocampus. Hum Gene Ther.

[b58] Papaioannou VE, Fox JG (1993). Efficacy of tribromoethanol anesthesia in mice. Lab Anim Sci.

[b59] Parish CA, Hashimoto M, Nakanishi K, Dillon J, Sparrow J (1998). Isolation and one-step preparation of A2E and iso-A2E, fluorophores from human retinal pigment epithelium. Proc Natl Acad Sci USA.

[b60] Radu RA, Mata NL, Bagla A, Travis GH (2004). Light exposure stimulates formation of A2E oxiranes in a mouse model of Stargardt's macular degeneration. Proc Natl Acad Sci USA.

[b61] Radu RA, Yuan Q, Hu J, Peng JH, Lloyd M, Nusinowitz S, Bok D, Travis GH (2008). Accelerated accumulation of lipofuscin pigments in the RPE of a mouse model for ABCA4-mediated retinal dystrophies following Vitamin A supplementation. Invest Ophthalmol Vis Sci.

[b62] Reich SJ, Auricchio A, Hildinger M, Glover E, Maguire AM, Wilson JM, Bennett J (2003). Efficient trans-splicing in the retina expands the utility of adeno-associated virus as a vector for gene therapy. Hum Gene Ther.

[b63] Sambrook J, Russell DW (2001). Molecular Cloning: A Laboratory Manual.

[b64] Simonelli F, Maguire AM, Testa F, Pierce EA, Mingozzi F, Bennicelli JL, Rossi S, Marshall K, Banfi S, Surace EM (2010). Gene therapy for Leber's congenital amaurosis is safe and effective through 1.5 years after vector administration. Mol Ther.

[b65] Sohocki MM, Daiger SP, Bowne SJ, Rodriquez JA, Northrup H, Heckenlively JR, Birch DG, Mintz-Hittner H, Ruiz RS, Lewis RA (2001). Prevalence of mutations causing retinitis pigmentosa and other inherited retinopathies. Hum Mutat.

[b66] Sun H, Nathans J (1997). Stargardt's ABCR is localized to the disc membrane of retinal rod outer segments. Nat Genet.

[b67] Testa F, Maguire AM, Rossi S, Pierce EA, Melillo P, Marshall K, Banfi S, Surace EM, Sun J, Acerra C (2013). Three-year follow-up after unilateral subretinal delivery of adeno-associated virus in patients with Leber congenital Amaurosis type 2. Ophthalmology.

[b68] Vandenberghe LH, Auricchio A (2012). Novel adeno-associated viral vectors for retinal gene therapy. Gene Ther.

[b69] Vandenberghe LH, Bell P, Maguire AM, Cearley CN, Xiao R, Calcedo R, Wang L, Castle MJ, Maguire AC, Grant R (2011). Dosage thresholds for AAV2 and AAV8 photoreceptor gene therapy in monkey. Sci Transl Med.

[b70] Venables VN, Ripley BD, Chambers SJ, Eddy W, Hardle W, Sheater S, Tierney L (2002). Modern Applied Statistics with S.

[b71] Wang Y, Ling C, Song L, Wang L, Aslanidi GV, Tan M, Srivastava A (2012). Limitations of encapsidation of recombinant self-complementary adeno-associated viral genomes in different serotype capsids and their quantitation. Hum Gene Ther Methods.

[b72] Weng J, Mata NL, Azarian SM, Tzekov RT, Birch DG, Travis GH (1999). Insights into the function of Rim protein in photoreceptors and etiology of Stargardt's disease from the phenotype in abcr knockout mice. Cell.

[b73] Wu J, Zhao W, Zhong L, Han Z, Li B, Ma W, Weigel-Kelley KA, Warrington KH, Srivastava A (2007). Self-complementary recombinant adeno-associated viral vectors: packaging capacity and the role of rep proteins in vector purity. Hum Gene Ther.

[b74] Wu L, Nagasaki T, Sparrow JR (2010a). Photoreceptor cell degeneration in Abcr (−/−) mice. Adv Exp Med Biol.

[b75] Wu Z, Yang H, Colosi P (2010b). Effect of genome size on AAV vector packaging. Mol Ther.

[b76] Yan Z, Lei-Butters DC, Zhang Y, Zak R, Engelhardt JF (2007). Hybrid adeno-associated virus bearing nonhomologous inverted terminal repeats enhances dual-vector reconstruction of minigenes in vivo. Hum Gene Ther.

[b77] Yan Z, Zak R, Zhang Y, Engelhardt JF (2005). Inverted terminal repeat sequences are important for intermolecular recombination and circularization of adeno-associated virus genomes. J Virol.

[b78] Yan Z, Zhang Y, Duan D, Engelhardt JF (2000). Trans-splicing vectors expand the utility of adeno-associated virus for gene therapy. Proc Natl Acad Sci USA.

[b79] Zhang Y, Chirmule N, Gao G, Wilson J (2000). CD40 ligand-dependent activation of cytotoxic T lymphocytes by adeno-associated virus vectors in vivo: role of immature dendritic cells. J Virol.

