# Supplementary material for: Effective delivery of large genes to the retina by dual AAV vectors
Source: EMBO Mol Med. 2013 Dec 16;6(2):194–211. doi: 10.1002/emmm.201302948 (PMC3927955; doi:10.1002/emmm.201302948)
Supplement: Supplementary file 1 [file emmm0006-0194-sd1.pdf]

Effective delivery of large genes to the retina by dual AAV vectors

I. Trapani, P. Colella, A. Sommella, C. Iodice, G. Cesi, S. De Simone, E. Marrocco, S. Rossi, M. Giunti, A. Palfi, G. Jane Farrar, R. Polishchuk and A. Auricchio

Corresponding author: Alberto Auricchio, Telethon Institute of Genetics and Medicine TIGEM

Review timeline:

Submission date:	18 May 2013
Editorial Decision:	12 June 2013
Additional Author Correspondence:	17 June 2013
Additional Editorial Correspondence	20 June 2013
Additional Editorial Correspondence:	01 July 2013
Revision received:	16 September 2013
Editorial Decision:	04 October 2013
Revision received:	10 October 2013
Accepted:	11 October 2013

Transaction Report:

(Note: With the exception of the correction of typographical or spelling errors that could be a source of ambiguity, letters and reports are not edited. The original formatting of letters and referee reports may not be reflected in this compilation.)

Editor: Roberto Buccione

1st Editorial Decision 12 June 2013

Thank you for the submission of your manuscript to EMBO Molecular Medicine. We have now heard back from the three Reviewers whom we asked to evaluate your manuscript.

You will see that while the Reviewers are generally supportive of you work (with varying degrees), a number of important concerns are expressed, which prevent us from considering publication at this time. I will not dwell into much detail, as the evaluations are detailed and self-explanatory.

Reviewer 1 is supportive of your study but would like to know if there where production issues for the vectors.

Reviewer 2 is also globally supportive but raises significant issues that require your action. S/he is especially concerned about the lack of a sufficiently critical analysis of the expression properties of the vectors and the potential expression of aberrant proteins. This Reviewer lists many important items that should be improved or supported with additional experimentation, better discussed or amended.

Reviewer 3 is quite critical and provides a thorough analysis. S/he notes that although the overall novelty of the work presented is relatively low, the translational value and advance is potentially significant, provided the many technical issues are properly dealt with and resolved. I share this opinion and thus strongly encourage you to carefully address this Reviewer's concerns. I would like

to highlight a few main points. Reviewer 3 feels that although the issue of the reliable production of homogeneous vectors appears to be solved, that of reproducible and adequate gene expression is not. S/he also notes the inadequacy of controls in certain contexts and the potential for formation of aberrant protein products that has not been well addressed (as mentioned Reviewer 2 as well). This Reviewer, while recognising the excellent transduction of the photoreceptor layer, mentions the occurrence of an unidentified cell mass, which was also apparent in a previous paper; s/he requires characterisation of this mass to explore its potential functional consequences. A main item of concern expressed by Reviewer 3 is, to use his/her own words, that "no added value to use a dual AAV vector system is presented here unless a demonstration is performed to show that a photoreceptor function restoration can be obtained..".

Considered all the above, while publication of the paper cannot be considered at this stage, we would be prepared to consider a substantially revised submission, with the understanding that the Reviewers' concerns must be fully addressed, with additional experimental data where appropriate and that acceptance of the manuscript will entail a second round of review.

Please note that it is EMBO Molecular Medicine policy to allow a single round of revision only and that, therefore, acceptance or rejection of the manuscript will depend on the completeness of your responses included in the next, final version of the manuscript.

As you know, EMBO Molecular Medicine has a "scooping protection" policy, whereby similar findings that are published by others during review or revision are not a criterion for rejection. However, I do ask you to get in touch with us after three months if you have not completed your revision, to update us on the status. Please also contact us as soon as possible if similar work is published elsewhere.

***** Reviewer's comments *****

Referee #1 (Remarks):

This is an excellent study that compares and contrasts various platforms for delivery and expression of large genes from AAV vectors. The motivation for this work, and the importance to the field, is that AAV application for therapy is limited to small cDNAs, something that precludes use for numerous disorders. Here, they use established and novel platforms and compare their efficacy in mice and pigs, and in mice models of retinal disease. The impact of the findings go beyond retinal gene therapy and hold promise for any disease amenable to AAV transduction.

The paper is written clearly and the figures support the conclusions. My only minor criticism is if the authors could address if there were any production issues as the vectors had elements that could promote recombination or splicing.

Referee #2 (Comments on Novelty/Model System):

The study by Trapani, Colella and colleagues focus on the performance of the newly developed dual AAV vectors which allow the transfer of large genes into cells for the treatment of inherited retinal degenerations (IRD). They use models of IRD that can be cured by the expression of the defective gene. As some of the IRD are based on defects of rather large genes that cannot be transferred by conventional AAV vectors, this is an important development.
Dual AAV vector have been applied for the treatment of Duchenne muscular dystrophy and hemophilia VIII which both rely on the transfer of large genes. There are previous reports on the usage of dual AAV vectors for the transduction of the retina, including in vivo animal models. The study by Trapani, Colella et al. is exceeding these studies slightly as they can also present the phenotypical correction in the mouse model. The disease models tested are the Ushers syndrome (defect in MYO7A and Stargardt's disease (defect of ABCA4). The latter was shown to be difficult to cure by AAV gene therapy due to large size of the gene in a recent paper this year by Charbel Issa et al., Plos One, 2013 which highlights the need for a better strategy.

The treatment of Leber congenital amaurosis by AAV gene therapy has already been applied successfully in clinical trials. The use of dual AAV vector, therefore, is of translational relevance given that the efficacy and safety can be proven in relevant models.
The paper is mainly focused on the performance of the vectors in vitro and in vivo, but would benefit from a more critical analysis of the expression properties of the vector and potential expression of aberrant proteins.

Referee #2 (Remarks):

The study by Trapani, Colella and colleagues aims to develop a treatment for inherited retinal degenerations, e.g. Stargardt's disease and Ushers syndrome. They developed dual AAV vectors for the expression of ABCA4 and Myo7A based on different strategies, overlapping, trans-splicing, hybrid vectors. Due to the large size of the genes and the limitation of conventional AVV vector to package these large cDNAs, dual AAV vectors are an interesting alternative.
In general, AAV base gene therapy has demonstrated its potential for the treatment of LCA in studies by the group and others , including clinical trials. Therefore, the use of dual AAV vectors could also be applied given that the efficacy is high enough.
The study is overall well performed and shows convincing therapeutic efficacy in animal model, however some questions remain, as specified below:

1. The dual AAV vectors my also express truncated RNA which will translate to truncated proteins. The Western blot in Fig. 2 does not allow assessing this issue. Can any other proteins be detected? Is the anti flag antibody able to detect others than full length protein variants of ABCA4?
2. What is the efficacy of co-transduction? How many cells are transduced by only one of the vectors? Is the ratio of the two vectors important for protein expression?
3. There is no expression of ABCA4 from the RHO and RHOK promoter after delivery by the AAV OV vector. This may be due to the low transduction of the PR, but could also be caused by the inability of the promoter to express in PR. This is not easy to assess as there is not positive control that the promoter would be active after efficient transduction. And, vice versa, it is not shown that the PR cells are not transduced because the statement is solely based on the expression levels.
4. Please mark the transduced photoreceptors on the pictures (Suppl. Fig. 3 and Fig. 4), as it will help the non-expert to appreciate the pictures.
5. There is no direct comparison of the dual AAV vectors to the oversized AAV in the in vivo models. Therefore it cannot be estimated how well the dual AAV approach performs in comparison to a conventional AAV or oversized AAV, This would be important to understand if this is a relevant strategy.
6. In the Fig. 6B, only two eyes were evaluated for the TS-ABCA4 vector; therefore no statistical relevance of this experimental group can be made, because the population is too small. Please remove the p-value.
7. Please indicate the n of the groups in the Fig. 7, similar to the Fig. 6 and 8.
8. All in vivo experiments have an observation time of max three months. Can the authors comment on the expression after longer observation times?
9. Please discuss in what respect your study exceeds the ones of others using dual AAV vectors in retina targeted therapy (e.g. Lopes et al., Gene Therapy 2013; Palfi et al., Human Gene Therapy 2012)

Referee #3 (Remarks):

During these last years, gene therapy has gone through important development and seems promising to treat diverse diseases with gene function deficiencies. Ocular gene therapy has demonstrated the feasibility to safely treat patients with RPE65 deficiency and in fact to restore visual function in several of them. Even if in many cases the degenerating process is not stopped, the prolongation of any visual function is a marked improvement in the quality of life. However, so far this therapy can be applicable only to a very small portion of the patients affected by recessive retinal diseases. Indeed, the targeted cells in these gene therapy clinical trials are the retinal pigment cells and the AAV vector used can only contain small cDNA. Unfortunately, the most frequent diseases in this

© EMBO

family of rare diseases cannot yet benefit from such therapy since they affect mainly the photoreceptors and moreover necessitate the transfer of large wild type cDNA. This is the case for the Husher syndrome (which is a devastating disease leading to blindness and then to deafness) and for the Stargadt disease. The development of a vector able to transfer large transgenes into photoreceptor cells is in consequence an important advance for ocular gene therapy as well as for other organs necessitating large gene construct transfer.
The authors of this article already challenged the possibility to integrate a large transgene into an AAV vector (referred to as oversized vector in the present article, Allocca et al., 2008). Although they observed a physiological improvement in a mouse model of the Stargadt disease, the vector packaging is problematic. Indeed, it seems that different pieces of the ABCA4 cDNA are included in the vector particles and that random homologous recombinations in the targeted cells lead to the expression of the full mRNA and proteins. This work shows the feasibility to generate the expression of a large transgene from pieces of a large cDNA sequence; however from a point of view of regulatory affairs, the non-reliability to generate vectors with a standard yield of the quality product precludes translating such technology to therapy.
In this project as an alternative approach to generating vectors in a controlled protocol, the authors propose to use a dual vector strategy where each vector contains a complementary piece of the cDNA gene sequence. Different methods were tested to obtain full sequences in the targeted cells such as homologous recombination, trans-splicing and combination of these two methods. These approaches were already documented by other groups in in vitro and in vivo studies (references within the article) but the present work uses original sequences to promote homologous recombination. These vectors were compared to oversized vectors. After having validated the method in vitro, the authors tested the vectors in two animal models of retinal dystrophies necessitating the transfer of large wild-type cDNA to restore some retinal function: the Abca4-/- and the shaker 1 mice which are (imperfect) models of Stargadt disease and Husher 1B syndrome respectively. First, the efficacy to obtain in vivo GFP expression in photoreceptors from a dual vector was tested in a normal retina. For the trans-splicing and the AK-hybrid systems 19% and 15% respectively of the retina show strong transduction levels; 31% and 38% are classified in intermediate level, when in fact very few photoreceptors are positive for GFP. The trans-splicing and the hybrid constructs are also functional in the pig retina: the positive data show that a large percentage of photoreceptors are amenable to support trans-splicing and recombination of the sequences tested. In mice, a total of around 50% of the injected retinas present GFP expression (with a large variation) in the photoreceptors with the dual vectors, whereas 100% of the retinas are strongly or "intermediately" transduced with the standard vector. Interestingly and maybe not enough underlined in the article, 100% of RPE cells seem to be transduced by the trans-splicing and the AK-hybrid vectors. The trans-splicing approach is not new and was already tested in the retina by the last author of this article, but the present work shows a tremendous increase in efficacy in the most transduced retina (but the efficiently transduced retinas represent around 20% of the cases).
The efficacy of the therapeutic transgene was then tested in the two animal models of inherited retinal dystrophy. Although in human, mutations in the ABCA4 and the MYO-VIIA genes lead to a marked alteration of the retinal function and morphology, these mutations in mice have only a mild effect rendering difficult the analysis of neuroprotective agents or therapeutic vectors as tested here to evaluate their benefit. Nonetheless, several lines of data presented in this work suggest that the dual vector approach is effective. However, some controls need to be better designed to unambiguously eliminate the potential protective effect of the surgical procedure and vector delivery. Moreover, the challenge to cure these diseases is to efficiently transfer a large transgene into the photoreceptors. In consequence, documentation showing the expression as well as function recovery in the target cells needs to be provided to confirm the gene transfer efficacy. Although the presented results demonstrate ABCA4 expression in photoreceptors, the data concerning MYO-VIIA do not well validate its expression in the photoreceptors. Moreover, the restoration of photoreceptor functions needs deeper characterization to prove the action of the transgene. The data concerning the RPE cell function restoration also need to be completed to be convincing. Nonetheless, this important work shows the feasibility to obtain in the retina an expression from a large gene that was previously split into two vectors

Major:

Major #1: The authors overcome their first problem with the oversized vectors which contain different sized sequence fragments of the transgene and are in consequence heterogeneous vectors. This situation is incompatible with clinical application requiring a characterized vector produced in

standard and reliable conditions. The dual vector system proposed here thus becomes an important progress for one step (i.e. the reliable production of homogenous vectors), but still faces the problem of reproducible and adequate gene expression. Indeed, the studies with the GFP expression show that less than 20% of the injected eyes present a satisfactory transduction level, moreover with a low expression level. Indeed, the 3 highest expression results with the dual vectors are 8 to 20 times lower than the average level measured with the classical vector. This hurdle to achieve reliable transduction and expression level would certainly impair clinical application. To be clinically relevant, the procedure needs to obtain a standardized efficacy of the expression level (in a certain range). In consequence, a better characterization of the dual infection effect is necessary to identify which titer and ratio of each vector is the optimum to promote recombination. In this study, the percentage of photoreceptors containing the two types of vectors and the percentage of the double-infected cells undergoing recombination are not known. Concatemerization of the vectors can produce different structures with or without the good vector alignment in the concatemer. The dose of the vector used, the sequence of the construct, etc., will have an impact on the probability to have the 5' vector sequence linked to the head of the 3'vector sequence. In consequence, the fact that "only" around 20% of the animals treated with the dual vectors show strong transduction may be simply due to an imperfect dosage and ratio of the vectors used. The fact that a strong transduction can happen with the dual vector strategy suggests that the procedure may be optimized to obtain a greater percentage of retinas with such expression pattern. In vitro experiments may already give important information on the possibility or not to improve the homologous recombination and the trans-splicing strategies. Real-time PCR can give a view on the vector positions in the concatemers and may help to identify whether a specific vector dose and ratio can favor certain concatemer arrangements. An in vivo study can then confirm the optimal condition observed in vitro.
In addition, the study of double injections with two vectors, one coding for GFP, the other for dsRed would help to determine the percentage of photoreceptors transduced with 1 and 2 vectors. This will help to establish whether all the double-transduced cells undergo rearrangement allowing large transgene expression, or if only a sub-population is concerned. This will help to dissect the mechanism favoring large gene expression from dual vectors and to improve the method.
The proof of principle of large gene transfer efficacy with dual vectors was already reported for in vivo applications by targeting muscle cells (Lai et al. Nat Biotechnology, 2005). The authors here need to better characterize their original methods to propose a reliable vector for large transgene transfer and expression in the retina.

Major #2: Another important concern is the use of inadequate controls for certain experiments. An important heterogeneity can be found throughout the experiments concerning the controls used and the control vectors expressing GFP. For instance, some experiments compare the dual vector effect with either no control vector (ex: Fig 6B ERG data) or a classical GFP vector instead of a dual GFP vector system which may have other effects than a single vector (ex: Fig: 8C). Details will be given below.

Major #3: For the in vitro studies, the supplementary methods should contain the full gel picture to reveal whether the homologous recombination and trans-splicing approach give only the good transgene size or if other products are present. The specificity of the recombination is important to validate to exclude the formation of other protein products that may interfere with the normal pathway.

Major #4: The experiments with GFP dual vectors demonstrate the feasibility to undergo adequate homologous recombination and trans-splicing. However, the controls are not fully appropriate. First, the vector dose is identical for each vector: in consequence in order to compare the dual vector system with the AAV-NS (classical one), the AAV-NS should be injected with a double dose. Secondly, the oversized vector, which contains a large backbone (9951 bp) is compared with dual vectors which carry 3416 to 3629 bp in total (addition of both vectors). The final size should be similar to the oversized vector and the other vectors containing the flanking sequences of the ABCA4 and MYOSIN VIIA cDNA (around 9500 bp in total). Many parameters may intervene with the homologous recombination process (including the concatemerization) and the vector size may be one of these parameters. The total length of the vector construct should be similar between the different vectors tested. This is true for the experiments described in Figures 2, 4, 5, 6, 7, 8, supporting Figures 1, 2, 3. The present data are important and show the feasibility of the systems tested with the dual vectors, but it is crucial to document what happens when large sequences are tested and, just as important, to assess whether such system has the same efficacy in a diseased

retina (shaker and Abca4-/- mice).

Major #5: in the pig experiment in Figure 4B, a beautiful strong transduction of the photoreceptor layer is shown with the two dual vector systems tested. However, a large unidentified cell mass or exudation is present at the extremity of the outersegments (mainly apparent in the picture AK-EGFP). Or are they altered outersegments? In their previous paper (Reich et al., 2003, Figure 4C) a similar mass is present between the outersegments and the RPE mouse retina. This needs to be better characterized with DAPI staining and other markers. In this context, retina slices of mice treated with ABCA4 and MYOSIN VIIA vectors should also be provided. This mass may interfere with the RPE and photoreceptor metabolism or reduce the access of the RPE to photoreceptor debris with a possible consequence of less lipofuscin granules in the RPE (Figure 5C and D). This mass may also contain macrophages which can phagocyte debris.

Major #6: concerning the experiments in the Abca4-/- mice, only the protein expression data are strong and very convincing. All the others suffer from the same concerns emitted in "Major#4 and #5". Moreover, Figures 5 D and 6B show graphs with statistics and sem bars when n=2 for two groups which is not reliable. In Figure 6, the ERG experiment compared ABCA4 dual vector-treated mice with WT and uninjected and 5'KO mice. A control KO mouse group treated with GFP dual vectors (large transgene) should be included. In conclusion, no convincing data are presented on a possible function of the ABCA4 transgene.

Major #7: concerning the experiments in the shaker mice, the authors present the expression data of the transgene in the eyecup and the retina, but some groups (such as the WT) contain n= 2, or n= 1 and statistics are made, which is not reliable with a so small animal number. The apical melanosome quantification is the most convincing data revealing the action of the MYOSIN VIIA protein in the RPE but it is necessary to consider using better vector controls (see Major #4). A single picture of higher magnification of Figure 8A with arrows will help to understand which organelles the authors have counted. In view of Figure 8C, it is surprising to see that no groups are different from the shaker mice. A verification of the statistics would be appropriate. Nonetheless, this study should also include shaker mice treated with dual GFP vectors.

Synthesis of the major concerns

From all these concerns, it appears that no clear and unambiguous data are presented to show that the dual vector system restores one or more functions in the photoreceptor cells. Previous studies (see Hashimoto et al., 2007) have shown that the lentiviral vector can effectively transduce the RPE and restore melanosome migration in the RPE cells as well as prevent abnormal opsin accumulation in the cilium of the shaker mice. In the Trapani work, no added value to use a dual AAV vector system is presented here unless a demonstration is performed to show that a photoreceptor function restoration can be obtained in these cells. The data obtained with RPE cells are promising and a careful experiment design should reveal whether the photoreceptor cells may also benefit from the dual vector strategy.

Minor:

Abstract: indicate in the 3rd sentence what the safety concern is.

The authors cannot write that the dual vectors transduce efficiently the photoreceptors when less than 20% of the injected retinas show efficient transduction.

So far the title is innapropriate, because overstated.

Many "data not shown" are present in the paper. Some of them would reinforce the paper.

Additional Author Correspondence 17 June 2013

Thank you very much for your feedback on our manuscript. I was pleased to see that all reviewers are supportive of our work, including Reviewer 3 who states "this important work shows the

feasibility to obtain in the retina an expression from a large gene that was previously split into two vectors ". I also want to underline the originality of our findings: although dual AAV vectors have been previously described, here we report novel strategies and provide new evidence that allow to overcome previous limitations (Lopes et al 2013) and to rescue for the first time retinal diseases using dual AAVs.

I have found most of the reviewers comments appropriate and we are performing additional experiments to address the following issues raised by the reviewers:
-provide data on production/yields of dual AAV vectors (Reviewer 1)
-investigate the potential presence of truncated proteins deriving from dual vectors (Reviewers 2 and 3)
-testing of different dual AAV ratios that could result in improved transduction (Reviewers 2 and 3)
-assessment of cells transduced by one vs two AAV vectors. We plan to accomplish this by using a single GFP and RFP expressing vectors as suggested. (Reviewers 2 and 3)
-clarify by H&E and marker staining that here is no cell mass in our dual AAV transduced retinas (Reviewer 3). In fact, so far we could not detect any abnormalities in the retinal structure that can be ascribed to dual AAV vectors.
-increase the n of eyes where requested (Reviewers 2 and 3)

However, we did not agree on some of Reviewer 3 major concerns. Specifically, concern #2 states "Another important concern is the use of inadequate controls for certain experiments. An important heterogeneity can be found throughout the experiments concerning the controls used and the control vectors expressing GFP " . In our paper we initially identify the best AAV-based platform for both RPE and photoreceptor large gene transduction and then test its therapeutic potential in relevant animal models of inherited retinal diseases, therefore we believe that the "important heterogeneity" in controls that he feels as a limitation is actually required by different experiments which have different goals. While the reviewer proposes that "the total length of the vector construct should be similar between the different vectors tested. This is true for the experiments described in Figures 2, 4, 5, 6, 7, 8, supporting Figures 1, 2, 3" , we agree that for the experiments shown in Fig. 2 and Suppl. 2 and 3 the size of the dual AAV EGFP vectors can be increased to match the size of oversized AAV used (and we are planning to di so in the revised version). However, we believe that the dual AAV EGFP vectors of normal size we have used in Fig. 4 and suppl. 1 have the most appropriate size for comparison to a normal size single AAV-EGFP. Similarly, in the Abca 4 -/- and Shaker 1 murine rescue experiments described in Figure 5, 6, 7 and 8, the size of the genome used in contralateral eyes is quite irrelevant. In these experiments we should have a control over the injection procedure: so we have included uninjected as well as single AAV-EGFP injected eyes. To be even more scrupulous, in rescue experiments we have injected contralateral eyes with single half vectors of dual AAV ABCA4 and MYO7A combinations to exclude any interference/biological activity that can in theory arise from a single half. In light of this, I don't think we need to repeat these very long rescue experiments to include as controls "dual AAV EGFP vectors (large transgene)" as suggested, which would not add anything to the controls we have already included. Therefore, I consider well sustained by the appropriate controls and without needs for further experiments, the conclusions from our rescue experiments in both Abca 4-/- as well as Shaker 1 mice presented in Figure 5, 6 and 8 that dual AAV vectors express transgenes in photoreceptors resulting in photoreceptor functional rescue.

I would like to have your feedback and advice on the plan that I have just outlined to be sure to head in the right direction in the revision of our paper. Thus, I wanted to ask your availability for a phone call to discuss this in person.

Additional Editorial Correspondence 20 June 2013

You made your points perfectly clear. I am in contact with the Reviewer to discuss these issues. I will be in touch soon.

Additional Editorial Correspondence 01 July 2013

I have now heard from the Reviewer. I apologise also on his/her behalf but s/he was traveling and had to deal with various commitments.

The reviewer believes that, considering the expectations generated by studies such as yours, special attention has to be paid that any progress made in this field is really a decisive step towards a therapeutic application and this is why s/he is very cautious,.

The animal models used in your study recapitulate some features only very partially, and the A2E compound, used in your 2008 JCI paper attesting of the function loss due to the Abca4 gene deletion in the KO mouse, now appears not significantly different between the transgenic mice and the control group, revealing a great heterogeneity in the expression of the phenotypes in this animal model.

Thus, demonstration of vector efficacy requires stringent controls, by providing a clear statistical analysis of your work, and by describing at best the parameters evaluated in these models. Moreover, since another vector is able to transfer in vivo a large gene into the Myo7a-/- RPE cells (the lentiviral vector, work by the Williams group), the originality of your present work is to potentially transfer large transgenes into photoreceptor cells, thus requiring unambiguous data about the functionality of the transferred gene into these cells.

Specifically, concerning the issues I discussed with the Reviewer:

1) Concerning the heterogeneity of control vectors expressing GFP, for instance, the experiments presented in Figures 6C, 7B and 8C and D do not integrate the appropriate controls. The Abca4-/- mice were treated on one hand with the dual EGFP/5'/3' vectors to analyze the RPE, which sounds relevant (the question of the total recombined transgene length remaining), and on the other hand the experiments in the same KO mice for ERG recording used only one EGFP/5' vector (or none) and not the dual EGFP/5'/3' system. This discrepancy of control vectors used reflects the "heterogeneity" term used in the Reviewersí initial report.

2) The simple control vector administration may provoke some beneficial effects as reported by different groups (including yours). As stated in your previous paper, the environmental conditions can affect the phenotype (such as retinal detachment, or the presence of microglia and macrophages) by diminishing the amount of debris to phagocyte for instance (one parameter measured by you when analyzing lipofuscin inclusions in the RPE). It is more difficult to obtain a good vector titer for a virus containing a large transgene than a small one. In addition, unknown mechanisms can also occur in the cells having received a vector with a large transgene which undergoes recombination. In consequence, it is important to use the same experimental design in the control group and with the therapeutic vectors: if you test 1) two vectors, 2) a recombination strategy (that may partially affect the cell metabolism), and 3) the production of a large therapeutic transgene, you would also need to challenge these three parameters in the control group when studying the shaker and the Abca4-/- mice (not for the EM study 5B which is convincing to show the presence of the protein in the photoreceptor segment). The main originality of this work would be the demonstration of transferring and generating large transgenes in the photoreceptor cells but no convincing data are presented in the present version (except the EM study). This side-by-side comparison (dual control vector with long transgene versus dual therapeutic vector with long transgene) is needed for these animal models, which don't present a marked phenotype in the retina with a strong loss of function and/or retinal degeneration.

Concerning experiments to repeat, in Figure 5D and Figure 6B, in some groups n=2 and the authors already need to complete their results with supplementary experiments. The inclusion of relevant controls should not be an excessive task.

An alternative would be to show in one of the animal models (preferentially the Abca4-/- mouse) that the dual control vector with the long transgene has no different effects on lipofuscin accumulation for instance, in comparison to a dual control vector with a "small" transgene.

I hope this clarifies these issues further.

In my opinion, this Reviewer has well taken points that require your action.

I'm sure you will understand that the decision on how to proceed is entirely up to you at this point. But rest assured that if you need extra time, this would not be a problem.

1st Revision - authors' response 16 September 2013

Reviewer 1

1. This is an excellent study that compares and contrasts various platforms for delivery and expression of large genes from AAV vectors. The motivation for this work, and the importance to the field, is that AAV application for therapy is limited to small cDNAs, something that precludes use for numerous disorders. Here, they use established and novel platforms and compare their efficacy in mice and pigs, and in mice models of retinal disease. The impact of the findings go beyond retinal gene therapy and hold promise for any disease amenable to AAV transduction. The paper is written clearly and the figures support the conclusions. My only minor criticism is if the authors could address if there were any production issues as the vectors had elements that could promote recombination or splicing.

We thank the reviewer for his extremely positive and encouraging comments. To address his point on potential dual AAV production problems we have included the following: i. titers of normal size (NS) vectors compared to dual AAV, which are similar (Materials and Method section, page 26, lines 557-558 and Supp. Table 2); Southern blot analysis of some of these vectors which shows a similar pattern between the genomes independently of the presence of the splicing/recombinogenic elements in dual AAVs (Materials and Method section, pages 26-27, lines 559-590 and Supp. Fig. 15). However, we acknowledge this potential problem which can be sequence-specific and thus should be addressed on a vector-by-vector basis (see Materials and methods section, page 26, lines 562-564).

Reviewer 2

1. The dual AAV vectors my also express truncated RNA which will translate to truncated proteins. The Western blot in Fig. 2 does not allow assessing this issue. Can any other proteins be detected? Is the anti flag antibody able to detect others than full length protein variants of ABCA4?

To answer the reviewer questions we evaluated the production of truncated/aberrant proteins from dual AAV trans-splicing (TS) and hybrid AK vectors encoding for either ABCA4 or MYO7A. In vitro and in vivo Western blot analyses were performed with antibodies that specifically recognize the 5'- and 3'-halves of the proteins as well as their full-length products. To achieve this, we had to generate additional constructs that were tagged with 3xflag at the 5' end of the ABCA4 cDNA (See Supp. Table 1 and Materials and methods section, page 24, lines 521-523).

For ABCA4 we found in vitro but not in the retina two protein products (>100 KDa) smaller than the full length that presumably derive from the transcription and translation of either the single 5'- or 3'-half of both dual AAV TS and hybrid AK vectors (Results section, page 14, lines 300-305 and Supp. Fig.7-8).

For MYO7A we found in vitro but not in the retina two protein products (<130 KDa) which are less abundant than the full length product: one that originates from the 5'-half and the other from the 3'-half of both dual AAV TS and hybrid AK vectors (Results section, page 16, lines 356-360 and Supp. Fig. 12-13). Although we did not observe protein products other than full-length in the mouse retina (Discussion section, page 21, lines 462-465), we think that the production of truncated/aberrant protein is an important issue that needs to be examined more in detail i.e. by high-throughput proteomic studies. Ultimately, long-term in vivo tox studies will be required to define the potential

toxicity deriving from dual AAV TS and hybrid AK vectors encoding for either ABCA4 or MYO7A. We discussed this in the Discussion section (page 21, lines 472-474).

Importantly, the inclusion of miRNA target sequences or proteasome targeting elements which are excluded from the full-length product may help to avoid production of truncated proteins from 5'- or 3'-half vectors.

2a. What is the efficacy of co-transduction? How many cells are transduced by only one of the vectors.

To answer this point we injected subretinally a combination of both a single vector expressing EGFP and a single vector expressing either RFP or DsRed (for this purpose we have generated two AAV plasmids encoding either RFP or DsRed which are now included in Supp. Table 1 and Materials and Methods section, page 25, lines 535-540). Three weeks later we evaluated the number of cells co-transduced by both vectors either by flow citometry of whole dissociated retinas or by counting the number of co-trasduced PR on retinal cryosections (Materials and Methods section, pages 33-34, lines 750-765 and page 32, lines 716-726). These are 36 ± 6% and 24±2% of total, respectively (Results section, page 9, lines 178-199 and Supp. Fig. 2). These levels of co-transduction in addition to the mechanism of gene reconstitution may be responsible for the reduced efficiency of dual AAV vectors when compared to a single NS vector, as we observe (Fig. 4A).

2b-Is the ratio of the two vectors important for protein expression?

To answer this question we injected subretinally C57BL/6 mice (Results section, page 12, lines 248-258 and Supp. Fig. 6) with dual AAV TS- and hybrid AK-EGFP at various half vector ratios and doses. We found that none of the various ratios tested outperforms the 1:1 ratio of the high dose of vectors used so far in our experiments, which corresponds to 2.5×10^9 GC/ mouse eye.

Although we can not exclude that doses higher than 10^9 GC/mouse eye can result in higher expression levels by dual AAV vectors, the limited volume imposed by the subretinal space in combination with the titers of vector preps does not allow to test this. We have discussed this in the Results section (page 12, lines 251-255).

3. There is no expression of ABCA4 from the RHO and RHOK promoter after delivery by the AAV OV vector. This may be due to the low transduction of the PR, but could also be caused by the inability of the promoter to express in PR. This is not easy to assess as there is not positive control that the promoter would be active after efficient transduction. And, vice versa, it is not shown that the PR cells are not transduced because the statement is solely based on the expression levels.

We and others have previously reported the ability of both the RHO and RHOK promoters to express reporter and therapeutic genes in murine and pig photoreceptors (PR) (Allocca et al. J Virol. 2007 Oct;81(20):11372-80; Mussolino et al. Gene Ther. 2011 Jul;18(7):637-45; Testa et al. Invest. Ophthalmol. Vis Sci. 2011 Jul 29;52(8):5618-24; Mihelec et al. Hum Gene Ther. 2011 Oct;22(10):1179-90; Khani et al. Invest Ophthalmol Vis Sci. 2007 Sep;48(9):3954-61). To follow upon the reviewer's comment, we have added to this manuscript a figure showing that both the AAV2/8-RHO- and -RHOK-EGFP vectors drive murine PR transduction with the former being at least as efficient as a similar vector containing the CMV promoter (Results section, page 11, lines 223-227 and Supp. Fig. 3).

Even more importantly, in Figure 5A-B we show the expression of ABCA4 from the RHO promoter using dual AAV TS and hybrid AK vectors, which clearly shows that the same RHO promoter drives ABCA4 PR transduction in the context of dual AAV vectors based on a different reconstitution mechanism than dual AAV overlapping (OV). Thus, the inefficient expression by the dual AAV OV vectors with the PR-specific promoters appears due to low homologous recombination in PR rather than low transcriptional activity of the promoters.

4. Please mark the transduced photoreceptors on the pictures (Suppl. Fig. 3 and Fig. 4), as it will help the non-expert to appreciate the pictures.

We marked the PR layer in Figure 4B, Supporting Figures 2-3-4, 5, 9.

5. There is no direct comparison of the dual AAV vectors to the oversized AAV in the in vivo models. Therefore it cannot be estimated how well the dual AAV approach performs in comparison to a conventional AAV or oversized AAV, This would be important to understand if this is a relevant strategy.

The direct comparison of dual AAV vectors, AAV oversize vectors (OZ) and conventional (normal size, NS) AAV vectors can be done with the EGFP transgene that is the only one which can be accommodated in a conventional AAV. We have done this comparison in the retina of C57BL/6 mice. Supporting Figure 4, that we have modified accordingly to Reviewer 3 major comment #4b (see below), clearly shows that the AAV OZ transduce PR less efficiently than the two most efficient dual AAV platforms (TS and hybrid AK). Based on these results we objectively quantified trangene expression in PRs after subretinal delivery of the dual AAV TS and hybrid AK in comparison to NS (conventional) AAV vectors (Fig. 4A) and found that PR transduction mediated by dual AAV vectors is significantly lower that that mediated by NS AAV vectors.

6. *In the Fig. 6B, only two eyes were evaluated for the TS-ABCA4 vector; therefore no statistical relevance of this experimental group can be made, because the population is too small. Please remove the p-value.*

We have increased to three the number of eyes treated with the TS-ABCA4 vectors in both Figure 6B and 5D and repeated the statistical analysis accordingly (Materials and Methods section, pages 37-38, lines 851 and 854-857).

7. *Please indicate the n of the groups in the Fig. 7, similar to the Fig. 6 and 8.*

In Figure 7A, the number of eyes analyzed was originally depicted. In Figure 7B, we increased the number of eyes analyzed which is now indicated.

8. All in vivo experiments have an observation time of max three months. Can the authors comment on the expression after longer observation times?

So far, we evaluated and reported ABCA4 expression up to 3 months after treatment (Fig. 5B) and MYO7A up to 9 months after treatment (Results section, page 16, lines 351-354). We added a comment about long-term transgene expression by dual AAV vectors in the Discussion section (pages 20-21, lines 451-455).

9. Please discuss in what respect your study exceeds the ones of others using dual AAV vectors in retina targeted therapy (e.g. Lopes et al., Gene Therapy 2013; Palfi et al., Human Gene Therapy 2012)

We discussed this in Discussion section (page 18, lines 393-398 and page 19, lines 408-410).

Up to now, little was known about the efficacy of dual AAV vector systems in the retina: Reich et al. reported expression of the b-galactosidase reporter gene (<4.7Kb) albeit at low levels in RPE and PR cells after subretinal delivery in mice of dual AAV TS vectors (Reich et al. Hum. Gene Ther. 2003 Jan 1;14(1):37-44). Palfi et al have indeed shown that the subretinal space favours co-trasdution of the same PR cell by two independent conventional AAV vectors carrying different reporter genes (Palfi et al. Hum. Gene Ther. 2012 Aug;23(8):847-58), a pre-requisite for transduction mediated by dual AAV OV, TS or hybrid vectors. However, when testing dual AAV OV vectors expressing MYO7A Lopes et al. failed to observe rescue of the retinal defects in *sh1-/-* mice (Gene Ther. 2013 Aug;20(8):824-33). Based on our data (Fig. 3 and 5A), we now know that this is due to the inefficient retinal homologus recombination required by the OV but not by the TS or hybrid approaches. Thus, our work that comprehensively compares for the first time the efficiency of AAV NS, AAV OZ, dual AAV TS and hybrid vectors in the retina has allowed us to select the most efficient strategy which we have then used to rescue two animal models of IRD.

Reviewer 3

Major #1a: The authors overcome their first problem with the oversized vectors which contain different sized sequence fragments of the transgene and are in consequence heterogeneous vectors. This situation is incompatible with clinical application requiring a characterized vector produced in standard and reliable conditions. The dual vector system proposed here thus becomes an important progress for one step (i.e. the reliable production of homogenous vectors), but still faces the problem of reproducible and adequate gene expression. Indeed, the studies with the GFP expression show that less than 20% of the injected eyes present a satisfactory transduction level, moreover with a low expression level. Indeed, the 3 highest expression results with the dual vectors are 8 to 20 times lower than the average level measured with the classical vector. This hurdle to achieve reliable transduction and expression level would certainly impair clinical application. To be clinically relevant, the procedure needs to obtain a standardized efficacy of the expression level (in a certain range).In consequence, a better characterization of the dual infection effect is necessary to identify which titer and ratio of each vector is the optimum to promote recombination.

In this study, the percentage of photoreceptors containing the two types of vectors and the percentage of the double-infected cells undergoing recombination are not known. Concatemerization of the vectors can produce different structures with or without the good vector alignment in the concatemer. The dose of the vector used, the sequence of the construct, etc., will have an impact on the probability to have the 5' vector sequence linked to the head of the 3'vector sequence. In consequence, the fact that "only" around 20% of the animals treated with the dual vectors show strong transduction may be simply due to an imperfect dosage and ratio of the vectors used. The fact that a strong transduction can happen ì with the dual vector strategy suggests that the procedure may be optimized to obtain a greater percentage of retinas with such expression pattern. In vitro experiments may already give important information on the possibility or not to improve the homologous recombination and the trans-splicing strategies. Real-time PCR can give a view on the vector positions in the concatemers and may help to identify whether a specific vector dose and ratio can favor certain concatemer arrangements. An in vivo study can then confirm the optimal condition observed in vitro.

To address the Reviewer concern about the possibility that the variable dual AAV-mediated expression might be due to the imperfect dosage and ratio of the vectors used, we tested in the murine retina (Results section, page 12, lines 248-258 and Supp. Fig. 6) different dosages and ratios and found that none of them outperforms the 1:1 ratio of the high dose of vectors used in our experiments, which corresponds to 2.5×10^9 GC/ mouse eye. Although we can not exclude that doses higher than 10^9 GC/mouse eye can result in higher expression levels by dual AAV vectors, the limited volume imposed by the subretinal space in combination with the titers of vector preps does not allow to test this. We have discussed this in the Results section (pages 12, lines 251-255).

There are strategies different than varying vector doses or ratios that can be used to implement the productive head-to-tail genome concatemerization required by dual AAV-mediated transduction. These include the use of heterologous ITRs (Yan et al. Hum. Gene Ther. 2007 Jan;18(1):81-7) as well as the addition of oligos which help direct the productive concatemerization of dual AAV genomes (Hirsch et al. PLoS One. 2009 Nov 2;4(11):e7705). We have added this to our Discussion section (page 20, lines 447-450). In addition, it is still possible that the inherent variability of the subretinal injection in the small murine eye contributes to the variable levels of transduction we have observed (Supp. Fig. 4). and that this may be at least partly reduced in larger eyes including human. While testing each of these hypothesis goes beyond the scope of this manuscript, we believe that the variable dual AAV vectors transduction in mice can be reduced and should not discourage future consideration of these strategies for clinical applications. Indeed, the effect of dual AAV vectors subretinal administration on the phenotypic rescue in animal models appears less variable than transgene levels, suggesting that a wide range of transgene expression levels can similarly contribute to therapeutic efficacy.

Major #1b: In addition, the study of double injections with two vectors, one coding for GFP, the other for dsRed would help to determine the percentage of photoreceptors transduced with 1 and 2 vectors. This will help to establish whether all the double-transduced cells undergo rearrangement

allowing large transgene expression, or if only a sub-population is concerned. This will help to dissect the mechanism favoring large gene expression from dual vectors and to improve the method. The proof of principle of large gene transfer efficacy with dual vectors was already reported for in vivo applications by targeting muscle cells (Lai et al. Nat Biotechnology, 2005). The authors here need to better characterize their original methods to propose a reliable vector for large transgene transfer and expression in the retina.

To answer this point we injected subretinally a combination of both a single vector expressing EGFP and a single vector expressing either RFP or DsRed (for this purpose we have generated two AAV plasmids encoding either RFP or DsRed which are now included in Supp. Table 1 and Materials and Methods section, page 25, lines 535-540). Three weeks later we evaluated the number of cells co-transduced by both vectors either by flow citometry of whole dissociated retinas or by counting the number of co-trasduced PR on retinal cryosections (Materials and Methods section, pages 33-34, lines 750-765 and page 32, lines 716-726). These are 36 ± 6% and 24±2% of total, respectively (Results section, page 9, lines 178-199 and Supp. Fig. 2). These levels of co-transduction in addition to the mechanism of gene reconstitution may be responsible for the reduced efficiency of dual AAV vectors when compared to a single NS vector, as we observe (Fig. 4A).

Major #2a: Another important concern is the use of inadequate controls for certain experiments. An important heterogeneity can be found throughout the experiments concerning the controls used and the control vectors expressing GFP. For instance, some experiments compare the dual vector effect with either no control vector (ex: Fig 6B ERG data) or a classical GFP vector instead of a dual GFP vector system which may have other effects than a single vector (ex: Fig: 8C). Details will be given below.

The controls to evaluate rescue in *Abca4-/-* and *sh1-/-* mice after subretinal delivery of dual AAV vectors included: i. uninjected as well as single AAV-EGFP injected eyes which mimick any potential trophic effect and/or damage caused by subretinal injections; ii. eyes injected with single half vectors of dual AAV ABCA4 and MYO7A combinations to prove that the ameliorations we observe are indeed due to the production of full-length ABCA4 and MYO7A proteins rather than to any potential truncated protein which derives from either the 5'-half or the 3'-half vector. However, we have included additional controls to our rescue experiments as per suggestion below:

In consequence, it is important to use the same experimental design in the control group and with the therapeutic vectors: if you test 1) two vectors, 2) a recombination strategy (that may partially affect the cell metabolism), and 3) the production of a large therapeutic transgene, you would also need to challenge these three parameters in the control group when studying the shaker and the Abca4-/- mice (not for the EM study 5B which is convincing to show the presence of the protein in the photoreceptor segment). The main originality of this work would be the demonstration of transferring and generating large transgenes in the photoreceptor cells but no convincing data are presented in the present version (except the EM study). This side-by-side comparison (dual control vector with long transgene versus dual therapeutic vector with long transgene) is needed for these animal models, which don't present a marked phenotype in the retina with a strong loss of function and/or retinal degeneration. Concerning experiments to repeat, in Figure 5D and Figure 6B, in some groups n=2 and the authors already need to complete their results with supplementary experiments. The inclusion of relevant controls should not be an excessive task.

An alternative would be to show in one of the animal models (preferentially the Abca4-/- mouse) that the dual control vector with the long transgene has no different effects on lipofuscin accumulation for instance, in comparison to a dual control vector with a "small" transgene.

According to the reviewer suggestion we have compared as control both dual vectors carrying short (dual AAV small with a combined length of 2.7-2.9 Kb) and long genomes (dual AAV large with a combined genome length of 8.9Kb) in both *Abca4 -/-* and *sh1-/-* animal models and evaluated: i.lipofuscin granules in *Abca4-/-* mice (Results section, page 15, lines 320-321 and Supp. Fig. 10); ii. recovery from light desensitization in *Abca4-/-* mice (Results section, page 15, lines 329-331) iii. rhodopsin accumulation at PR connecting cilium in *sh1-/-* mice (Results section, page 17, line 374 and Supp. Fig. 14, performed in albino *shaker 1* mice which were the only available at the time of the revision). The results show that none of the control vectors ameliorates the retinal defects of either *Abca4-/-* mice or *sh1-/-* mice independently of the control vector genome size. We thus

conclude that the therapeutic effect we observe in mice are specifically due to the expression of either ABCA4 or MYO7A in photoreceptors (PR).

Major #2b. The main originality of this work would be the demonstration of transferring and generating large transgenes in the photoreceptor cells but no convincing data are presented in the present version (except the EM study).

Since the Rhodopsin (RHO) promoter is active specifically in PR (Supp. Fig. 3; Allocca et al. J Virol. 2007 Oct;81(20):11372-80; Mussolino et al. Gene Ther. 2011 Jul;18(7):637-45), we clearly and unambiguously prove that both the ABCA4 (Fig. 5A-B) and MYO7A proteins (Results section, page 16, lines 354-356 and Supp. Fig. 11) are specifically expressed in PR after subretinal delivery of dual AAV trans-splicing (TS) and hybrid AK vectors.

Major #3: For the in vitro studies, the supplementary methods should contain the full gel picture to reveal whether the homologous recombination and trans-splicing approach give only the good transgene size or if other products are present. The specificity of the recombination is important to validate to exclude the formation of other protein products that may interfere with the normal pathway.

To answer this as well as a similar question from Reviewer 2, we evaluated the production of truncated/aberrant proteins from dual AAV TS and hybrid AK vectors encoding for either ABCA4 or MYO7A. In vitro and in vivo Western blot analyses were performed with antibodies that specifically recognize the 5'- and 3'-halves of the proteins as well as their full-length products. To achieve this, we had to generate additional constructs that were tagged with 3xflag at the 5' end of the ABCA4 cDNA (See Supp. Table 1 and Materials and methods section, page 24, lines 521-523).

For ABCA4 we found in vitro but not in the retina two protein products (>100 KDa) smaller than the full length that presumably derive from the transcription and translation of either the single 5'- or 3'-half of both dual AAV TS and hybrid AK vectors (Results section, page 14, lines 300-305 and Supp. Fig. 7-8).

For MYO7A we found in vitro but not in the retina two protein products (<130 KDa) which are less abundant than the full length product: one that originates from the 5'-half and the other from the 3'-half of both dual AAV TS and hybrid AK vectors (Results section, page 16, lines 356-360 and Supp. Fig. 12-13).

Although we did not observe protein products other than full-length in the mouse retina (Discussion section, page 21, lines 462-465), we think that the production of truncated/aberrant protein is an important issue that needs to be examined more in detail i.e. by high-throughput proteomic studies. Ultimately, long-term in vivo tox studies will be required to define the potential toxicity deriving from dual AAV TS and hybrid AK vectors encoding for either ABCA4 or MYO7A. We discussed this in the Discussion section (page 21, lines 472-474).

Importantly, the inclusion of miRNA target sequences or proteasome targeting elements which are excluded from the full-length product may help to avoid production of truncated proteins from 5'- or 3'-half vectors.

Major #4a: The experiments with GFP dual vectors demonstrate the feasibility to undergo adequate homologous recombination and trans-splicing. However, the controls are not fully appropriate. First, the vector dose is identical for each vector: in consequence in order to compare the dual vector system with the AAV-NS (classical one), the AAV-NS should be injected with a double dose.

If we inject the AAV normal size (NS) at double the dose of each of the dual AAV vectors, even if all 5' and 3' dual AAV vectors undergo productive recombination, the number of dual AAV concatemer genomes will be half of the AAV NS. In other words, we used the same dose of each vector because we considered that one genome copy (GC) of the 5'-vector plus one GC of the 3'-vector of dual AAVs are required to re-constitute one full-size functional genome as that contained in one particle of AAV NS. However by doing this we acknowledge that we have administered double the dose of dual AAV viral capsids compared to AAV NS. To exclude competition between dual AAV capsids at the entry step which may lead us to over-estimate the efficiency of AAV NS, we evaluated EGFP expression after subretinal delivery of either $1.7x10^9$ GC of AAV2/8 NS-EGFP

or $1.7x10^9$ GC of AAV2/8-NS-EGFP + $1.7x10^9$ GC of an AAV2/8 vector carrying an unrelated transgene (AAV-unrelated, Supp. Fig. 16). Notably, we found no significant differences in the levels of EGFP expression whether the unrelated AAV was added or not (Materials and Methods, pages 29-30, lines 647-659 and Supp. Fig. 16), proving that the double dose of dual AAV capsids administered when compared to AAV NS does not affect dual AAV-mediated transduction.

Major #4b: Secondly, the oversized vector, which contains a large backbone (9951 bp) is compared with dual vectors which carry 3416 to 3629 bp in total (addition of both vectors). The final size should be similar to the oversized vector and the other vectors containing the flanking sequences of the ABCA4 and MYOSIN VIIA cDNA (around 9500 bp in total). Many parameters may intervene with the homologous recombination process (including the concatemerization) and the vector size may be one of these parameters. The total length of the vector construct should be similar between the different vectors tested. This is true for the experiments described in Figures 2, 4, 5, 6, 7, 8, supporting Figures 1, 2, 3. The present data are important and show the feasibility of the systems tested with the dual vectors, but it is crucial to document what happens when large sequences are tested and, just as important, to assess whether such system has the same efficacy in a diseased retina (shaker and Abca4-/- mice).

The total length of the vector construct is similar between: i. AAV-CMV-NS-EGFP=3006 bp and -TS-EGFP=3416 bp or -AK-EGFP=3629 bp in Supporting Figure 1 (and corresponding Results section, page 9, lines 170-172); ii. AAV-RHO-NS-EGFP=2900 bp and -TS-EGFP=3312 bp or -AK-EGFP=3525 bp in Figure 4.

For those experiments in which we have included AAV oversize (OZ) vectors, whose genome is 9954 bp long, following the reviewer suggestion we have generated new dual AAV-TS- and AK-EGFP-L vectors whose genome size is 8567 bp and 8767 bp, respectively (Supp. Table 1; Results section, page 8, lines 162-164 and Materials and Methods section, page 23, lines 504-506). We did not generate dual AAV-AP-EGFP-L (and thus decided to omit the dual AAV-EGFP data previously presented with AP vectors with smaller genomes in Figure 2 and Supporting Figure 4) as our experiments with ABCA4 and MYO7A in Figure 2A-B clearly show that the AP approach results in weaker transgene expression than dual AAV TS and hybrid AK. We have thus used the new dual AAV-TS- and AK-EGFP-L vectors in Figure 2C, 2F and Supporting Figure 4. Finally, we show expression of the large ABCA4 and MYO7A genes by dual AAV vectors in both wild-type (Fig. 5A and 7A) and diseased *Abca4-/-* and *sh1-/-* retina, respectively (Fig. 5B, 7B and Supp. Fig. 11, 13).

Major #5a: in the pig experiment in Figure 4B, a beautiful strong transduction of the photoreceptor layer is shown with the two dual vector systems tested. However, a large unidentified cell mass or exudation is present at the extremity of the outersegments (mainly apparent in the picture AK-EGFP). Or are they altered outersegments? In their previous paper (Reich et al., 2003, Figure 4C) a similar mass is present between the outersegments and the RPE mouse retina. This needs to be better characterized with DAPI staining and other markers. This mass may interfere with the RPE and photoreceptor metabolism or reduce the access of the RPE to photoreceptor debris with a possible consequence of less lipofuscin granules in the RPE (Figure 5C and D). This mass may also contain macrophages which can phagocyte debris.

To address the reviewer's point we included in Figure 4B the DAPI staining of pig retinal sections (please see new Figure 4B) which shows no cell mass at the extremity of the outer segments. To further prove that the "cell mass/exudation" indicated by the reviewer corresponds to normal outer segments, we immuno-labelled the pig retinal cryo-section with antibodies directed to known outer segments markers (rhodopsin and cone-arrestin), and analyzed the retinal sections by confocal microscopy (see picture below). The analysis shows that outer segments of pigs treated with dual AAV TS and hybrid AK are labeled by both anti-rhodopsin and anti-cone-arrestin antibodies. In addition, since the same puffed appearance of pig outer segments is found in both treated as well as untreated pig eyes we conclude that this is the consequence of cryo-sectioning rather than dual AAV vector-related "exudation" or damage.

Major #5b: In this context, retina slices of mice treated with ABCA4 and MYOSIN VIIA vectors should also be provided.

We have now provided slices of murine retinas treated up to 3-8 months before with either dual AAV- MYO7A or -ABCA4 vectors, respectively, which show no histological abnormalities (Results section, page 14, lines 305-307 and page 17 lines 374-377; Supp. Fig. 9; Discussion section, page 21, lines 471-472).

Major #6: concerning the experiments in the Abca4-/- mice, only the protein expression data are strong and very convincing. All the others suffer from the same concerns emitted in "Major#4 and #5". Moreover, Figures 5D and 6B show graphs with statistics and sem bars when n=2 for two groups which is not reliable. In Figure 6, the ERG experiment compared ABCA4 dual vector-treated mice with WT and uninjected and 5'KO mice. A control KO mouse group treated with GFP dual vectors (large transgene) should be included. In conclusion, no convincing data are presented on a possible function of the ABCA4 transgene.

Please see answers to Major #2, #4 and #5 for a detailed description of the experimental controls we have included to support that the rescue we are observing in the diseased *Abca4 -/-* retina (both lipofuscin reduction and improved recovery from light desensitization) is indeed specific of dual AAV-mediated expression of the large ABCA4 transgene. These controls include: i. control dual AAV-TS- and AK vectors carrying transgene constructs of large size, similar to the therapeutic transgene (Major concerns #2 and 4); ii. histological evidence that the *Abca4-/-* retinas treated with dual AAV vectors do not present abnormalities which may be responsible for lipofuscin reduction (Major concern #5).

In addition, we have increased the number of eyes in both Figure 5D and 6B and repeated the statistical analysis accordingly (Materials and Methods section, pages 37-38, line 851 and 854-857).

Major #7a: concerning the experiments in the shaker mice, the authors present the expression data of the transgene in the eyecup and the retina, but some groups (such as the WT) contain n= 2, or n= 1 and statistics are made, which is not reliable with a so small animal number.

We have increased the number of eyes in Figure 7B and repeated the statistical analysis accordingly (Materials and methods section, page 37, lines 851-852 and Results section, page 16, lines 349-351).

Major #7b: The apical melanosome quantification is the most convincing data revealing the action of the MYOSIN VIIA protein in the RPE but it is necessary to consider using better vector controls (see Major #4). A single picture of higher magnification of Figure 8A with arrows will help to understand which organelles the authors have counted.

We have used better vector controls in *sh1-/-* mice to address the Reviewer concern in PR (Results section, page 17, line 374 and Supp. Fig. 14), which appeared more important than in RPE. Regarding RPE melanosomes, we have modified Figure 8A as suggested by the reviewer.

Major #7c: In view of Figure 8C, it is surprising to see that no groups are different from the shaker mice. A verification of the statistics would be appropriate

We increased the number of eyes in Figure 8C: while the ANOVA p value remained not significant (ANOVA p value=0.11), the Student's t-test evidenced significant differences between *sh1-/-* treated with MYO7A-TS and *sh1-/-* treated with control vectors (p value=0.039) but not *sh1 +/+* mice (p value=0.950). This is now indicated in Figure 8C and in the corresponding Results section, page 17, lines 372-373.

Major #7d: Nonetheless, this study should also include shaker mice treated with dual GFP vectors.

Please see the answer to major concerns #2, 4 and 5. As for the *Abca4 -/-* mice, to convincingly show that the rescue we are observing in the *sh1 -/-* PRs is specifically due to dual AAV-mediated large MYO7A transgene expression we provide the following controls: i. control dual AAV-TS and AK vectors carrying transgene constructs of large size, similar to the therapeutic transgene (Major concerns #2 and 4); ii. histological evidence that the *sh1-/-* retinas treated with dual AAV vectors do

© EMBO

not present abnormalities which may be responsible for the corrected rhodopsin localization (Major concern #5).

8. Synthesis of the major concerns. From all these concerns, it appears that no clear and unambiguous data are presented to show that the dual vector system restores one or more functions in the photoreceptor cells. Previous studies (see Hashimoto et al., 2007) have shown that the lentiviral vector can effectively transduce the RPE and restore melanosome migration in the RPE cells as well as prevent abnormal opsin accumulation in the cilium of the shaker mice. In the Trapani work, no added value to use a dual AAV vector system is presented here unless a demonstration is performed to show that a photoreceptor function restoration can be obtained in these cells. The data obtained with RPE cells are promising and a careful experiment design should reveal whether the photoreceptor cells may also benefit from the dual vector strategy.

We believe we have proven the added value of dual AAV vectors compared to lentiviral vectors for PR gene therapy in light of the new experiments performed which show: i. dual AAV-mediated ABCA4 and MYO7A expression using the PR-specific Rhodopsin promoters (which has not been shown so far with lentiviral vectors in the adult retina); ii. that the *Abca4-/-* and *sh1-/-* PR rescue observed is indeed specific of dual AAV-mediated ABCA4 and MYO7A expression since it is not observed in eyes treated with control vectors of the appropriate size and is not associated with retinal histological abnormalities induced by dual AAV vector administration.

Minor

9. Abstract: indicate in the 3rd sentence what the safety concern is.

We indicated the concerns (Abstract, page 2, lines 27-28)

10. The authors cannot write that the dual vectors transduce efficiently the photoreceptors when less than 20% of the injected retinas show efficient transduction. So far the title is inappropriate, because overstated.

Our title refers to the transduction obtained with dual AAV vectors as effective (not efficient) since it is associated with therapeutic efficacy in animal models. In addition, the title refers to the retina (which includes the RPE) rather than PR alone. We believe that the current manuscript proves that we are obtaining effective gene transfer in both RPE and PR and so we propose to leave the word effective in the title. However, if the reviewer still feels strongly about it we can delete" effective".

11. Many "data not shown" are present in the paper. Some of them would reinforce the paper.

We have reduced the number of "data not shown" in the paper. However please note that the current version of our manuscript already includes 8 main (the maximum allowed by the Journal) and 16 Supporting Figures.

2nd Editorial Decision 04 October 2013

Thank you for the submission of your revised manuscript to EMBO Molecular Medicine. We have now received the enclosed reports from the Reviewers that were asked to re-assess it. As you will see the reviewers are now globally supportive and I am pleased to inform you that we will be able to accept your manuscript pending the following final amendments:

1) Reviewer 1 notes that in response to a Reviewer, the type of statistical test employed was changed in an effort to determine significance. S/he does not agree with the change nor that significance is proven and thus suggests that this be amended.
2) Reviewer 3 would like you to better describe the controls in each experiment and notes that error bars needs to be calculated for Fig. 2 and supplementary Fig. 1.
3) As per our Author Guidelines, the description of all reported data that includes statistical testing

must state the name of the statistical test used to generate error bars and P values, the number (n) of independent experiments underlying each data point (not replicate measures of one sample), and the actual P value for each test (not merely 'significant' or 'P < 0.05').

I am prepared to make an editorial decision on your manuscript provided the above is carefully and completely dealt with.

Please submit your revised manuscript within two weeks. I look forward to seeing a revised form of your manuscript as soon as possible.

***** Reviewer's comments *****

Referee #1 (Remarks):

This revision has met the concerns raised in my prior review. The only new comment is that in response to one criticism from another reviewer, a different statistical test was used to determine significance. The change from an ANOVA to a t-test is not correct. The fact that there is a trend towards significant differences is all one can say. That doesn't mean the data is not useful. Most of the other data are significant.

Referee #2 (Remarks):

No further comments

Referee #3 (Comments on Novelty/Model System):

concerning the novelty, I would prefer to put medium/high because the strategy is not really new, but it is the first time that this approach shows efficacy for retinal diseases (using small modifications of the original approaches) and may have a very important impact if the future preclinical tests will show safety and efficacy in large animals (toxicology tests etc.).

Referee #3 (Remarks):

In the previous evaluations, many concerns were emitted to propose experiments to ascertain the dual vector strategy used in this study. The main concern was the use of inappropriate controls. In the present version, the authors made a major effort to respond to all points and the results reveal unambiguously the efficacy of the dual vectors tested. In consequence, this work will have not only a great interest for ophthalmology, but also for the entire field of gene therapy.

Minor:
The manuscript will gain in clarity if the controls are better described in each experiment with the n number of experiments for each vector tested and the titer used (as already presented for the therapeutic vectors).
Fig 2D, E and F: an error bar has to be calculated for the TS and TS-L group. These groups represent the 100% (reference value) and the internal variation has to be shown. Idem for Supporting Figure 1

2nd Revision - authors' response 10 October 2013

Thank you for reviewing our manuscript "Effective delivery of large genes to the retina by dual AAV vectors". We found the reviewers comments very positive and consider their further suggestions helpful in revising the manuscript. The enclosed version and this letter takes into account all the reviewer's comments.

In particular:

1) Accordingly to Reviewer 1, we have removed the asterisk from Fig.8C which represented significant differences obtained using the Student's t-test and evaluated the statical significance of the data using the ANOVA analysis only (as in the original version of the manuscript).
2) Accordingly to Reviewer 3, we have added more details to describe the control vectors used in our experiments.
3) We already acknowledged and discussed the lack of error bars for reference values depicted in Fig. 2 and Supp. Fig. 1 (Materials and Methods section, pages 31, lines 705-708) which is due to the use of one internal reference sample for each independent experiment. However in response to Reviewer 3 comments we have estimated the variability of our reference samples relatively to another sample from the same experiment and have reported the results in the Materials and Methods section (pages 31-32, lines 708-711).
4) We better described the statistical analysis accordingly to the author guidelines including the name of the statistical test used, the number of independent experiments and the actual p value. The details on the statistical analysis are now reported in the Statistical analysis paragraph of the Materials and Methods section and in Figure Legends.

We hope that this revised manuscript is now suitable for publication in *EMBO Molecular Medicine.*

Point-by-point answer to the Reviewers

Referee #1 (Remarks):

This revision has met the concerns raised in my prior review. The only new comment is that in response to one criticism from another reviewer, a different statistical test was used to determine significance. The change from an ANOVA to a t-test is not correct. The fact that there is a trend towards significant differences is all one can say. That doesn't mean the data is not useful. Most of the other data are significant.

We thank the Reviewer for his/her suggestion. We removed the asterisk from Fig. 8C and modified accordingly the corresponding Results (page 17, lines 374-376) and Materials and Methods (page 39, lines 886-887) sections. Therefore the assessment of the statical significance of these data is now based only on the ANOVA (as in the original version of the manuscript).

Referee #3 (Remarks):

Minor:

1. *The manuscript will gain in clarity if the controls are better described in each experiment with the n number of experiments for each vector tested and the titer used (as already presented for the therapeutic vectors).*

We added more details to the controls used in the following: recovery from light desensitization (Results section, page 15, lines 328-332); Legend to Fig. 4B (page 59, lines 1332-1334); Legend to Fig. 5 (page 60, lines 1365-1369); Legend to Fig. 6 (page 61, lines 1379-1381 and lines 1387-1390); Legend to Fig. 8 (page 63, lines 1429-1432 and lines 1436-1438); Legend to Supp. Fig. 4; Legend to Supp. Fig. 8-10; Legend to Supp. Fig. 13-14.

2. *Fig 2D, E and F: an error bar has to be calculated for the TS and TS-L group. These groups represent the 100% (reference value) and the internal variation has to be shown. Idem for Supporting Figure 1*

We already acknowledged this in the Materials and Methods section (page 31, lines 708-708). The TS and TS-L groups in Fig. 2 and the NS group in Supp. Fig. 1 do not have error bars as we performed several (n=3-4 independent experiments) AAV infections of HEK293 and Western blot analysis and analyzed them at different times. Briefly, for each independent experiment we performed SDS-PAGEs including one sample of each type so that we could have a side-by-side comparison of the various approaches on one gel. Thus, each experiment was independently performed and analyzed. Since different Western blots have different exposure times, which may dramatically influence the intensity of the bands, we decided to use a single sample (different in each experiment) loaded on each SDS-PAGE as internal reference for the gel. We used as internal reference the TS and TS-L samples for data depicted in Fig. 2 and the NS sample for data depicted in Supp. Fig. 1. We set these to 100, and the other samples from the same gel were calculated as % relative to TS (Fig 2D-E), TS-L (Fig. 2F) or NS (Supp. Fig. 1B). We can not average the TS, TS-L or NS values from the different gels which have different exposure times, therefore there is no average value (and in consequence no corresponding error bar) for TS, TS-L and NS values.

However to show the internal variation of TS, TS-L and NS samples we calculated their % values ±s.e.m relative to the AK sample (set to 100%) and reported these values in the Materials and Methods section (pages 31, lines 708-711).
