# Supplementary material for: Effective delivery of large genes to the retina by dual AAV vectors
Source: EMBO Mol Med. 2013 Dec 16;6(2):194–211. doi: 10.1002/emmm.201302948 (PMC3927955; doi:10.1002/emmm.201302948)
Supplement: Supplementary file 2 [file emmm0006-0194-sd2.pdf]

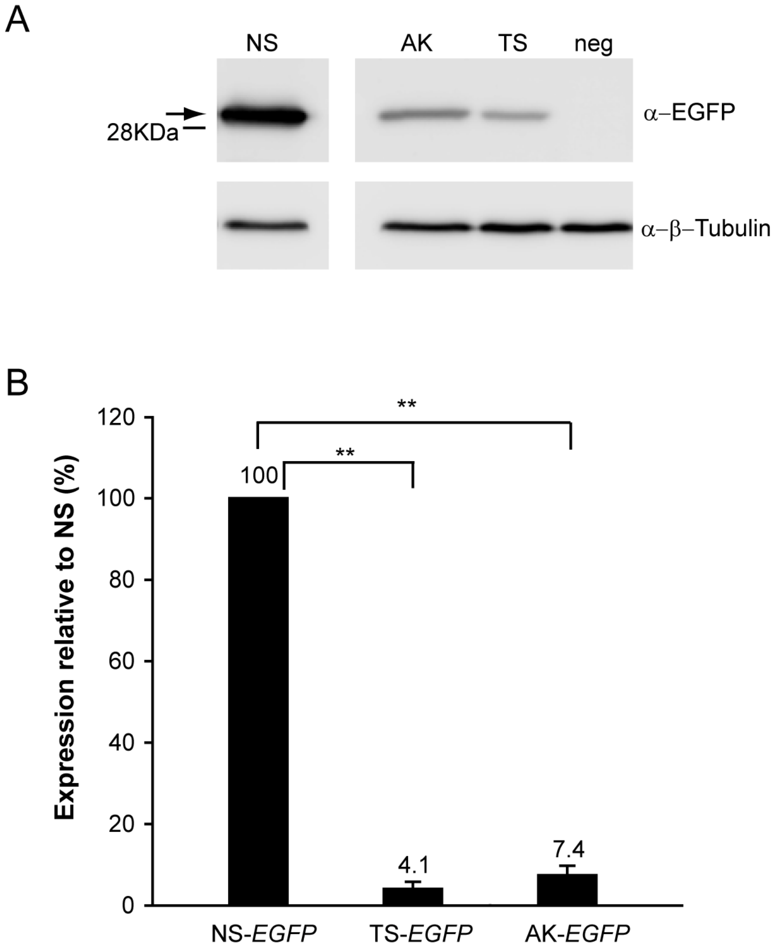

Supporting Figure 1. In vitro transduction efficiency of dual AAV trans-splicing and hybrid AK vectors compared to single normal size AAV vector.

(A) Representative Western blot analysis of HEK293 cells infected with AAV2/2 vectors encoding for EGFP under the control of the ubiquitous cytomegalovirus (CMV) promoter. The arrow indicates full-length protein, 50 micrograms of proteins were loaded in each lane, the molecular weight ladder is depicted on the left. (B) Quantification of EGFP protein bands. The intensity of the EGFP bands was divided by the intensity of the Tubulin bands. The histograms show the expression of proteins as percentage relative to single normal size AAV (NS) vector, the mean value is depicted above the corresponding bar. Values are represented as mean ± s.e.m. (standard error of the mean). The Western blot images are representative of and the quantifications are from n=3 independent experiments. NS: cells infected with normal size AAV vector; TS: cells infected with dual AAV trans-splicing vectors; AK: cells infected with dual AAV hybrid AK vectors; neg: cells infected with the 5'-half of either the dual AAV TS or hybrid AK vectors, as negative controls; α-EGFP: Western blot with anti-EGFP antibody; α-β-Tubulin: Western blot with anti-β-Tubulin antibody, used as loading control. **p ANOVA<0.001. More details on the NS variability as well as on the statistical analysis including specific statistical values can be found in the Western blot and Statistical analysis paragraphs of the Materials and Methods section, respectively.
