# Supplementary material for: Effective delivery of large genes to the retina by dual AAV vectors
Source: EMBO Mol Med. 2013 Dec 16;6(2):194–211. doi: 10.1002/emmm.201302948 (PMC3927955; doi:10.1002/emmm.201302948)
Supplement: Supplementary file 3 [file emmm0006-0194-sd3.pdf]

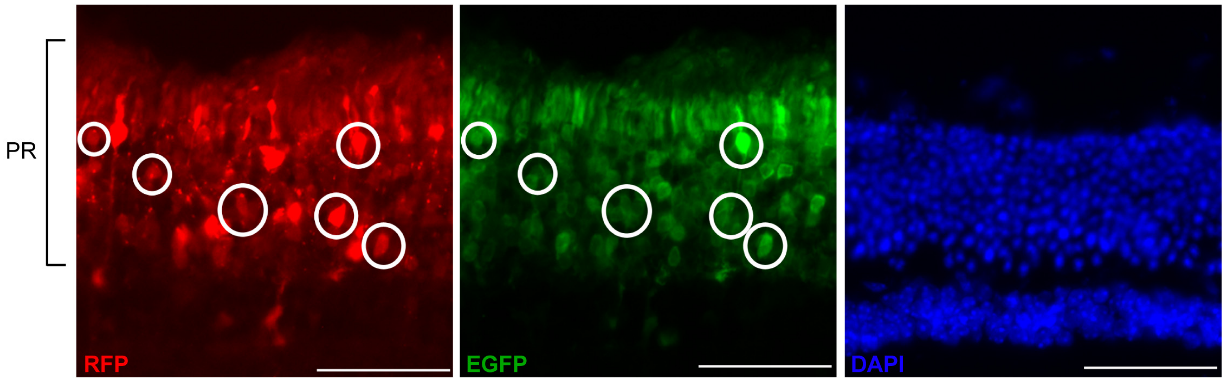

Supporting Figure 2. Photoreceptor co-transduction following subretinal combined delivery of single AAV-EGFP and -RFP vectors.

Fluorescence analysis of a representative retinal cryosection from C57BL/6 mice 3 weeks following subretinal co-injection of single AAV2/8-CMV-EGFP and-RFP vectors (dose of each vector/eye: $1.4x10^9$ GC, n=6 eyes) demonstrating RFP+ cells (left panel) and EGFP+ cells (central panel) is presented. The scale bar (50 µm) is depicted in the figure. Circles highlight photoreceptors transduced by both vectors. PR: photoreceptors; RFP: native RFP fluorescence; EGFP: native EGFP fluorescence; DAPI: 4',6'-diamidino-2-phénylindole staining.
