# Supplementary material for: Effective delivery of large genes to the retina by dual AAV vectors
Source: EMBO Mol Med. 2013 Dec 16;6(2):194–211. doi: 10.1002/emmm.201302948 (PMC3927955; doi:10.1002/emmm.201302948)
Supplement: Supplementary file 4 [file emmm0006-0194-sd4.pdf]

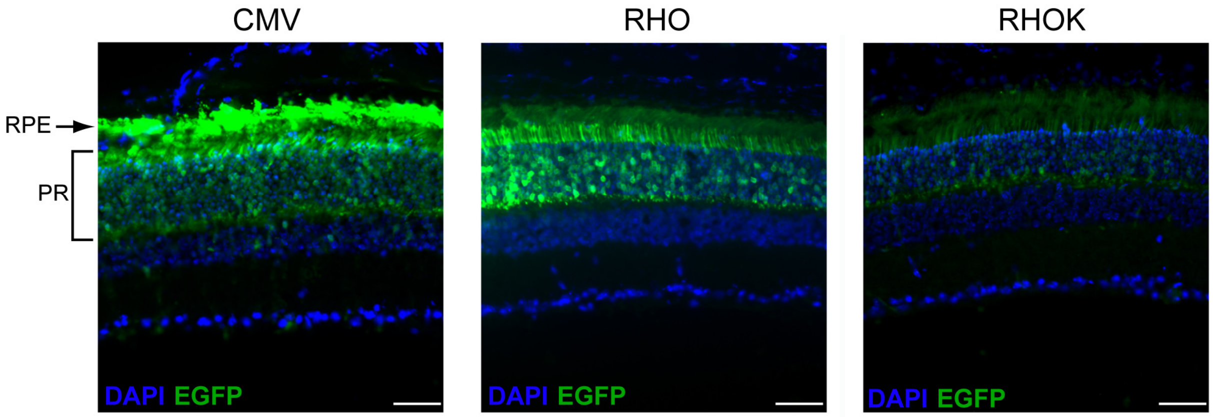

Supporting Figure 3. CMV, RHO and RHOK promoters drive transgene expression in murine photoreceptors.

Fluorescence analysis of representative retinal cryosections from C57BL/6 mice one month following subretinal injection of single AAV2/8 vectors encoding for EGFP under the control of the ubiquitous cytomegalovirus (CMV) promoter or the PR-specific Rhodopsin (RHO) and Rhodopsin kinase (RHOK) promoters. Arrow points at transduced RPE. The scale bar (50 µm) is depicted in the figure. RPE: retinal pigmented epithelium; PR: photoreceptors; DAPI: 4',6'-diamidino-2-phenylindole staining; EGFP: native EGFP fluorescence.
