# Supplementary material for: Effective delivery of large genes to the retina by dual AAV vectors
Source: EMBO Mol Med. 2013 Dec 16;6(2):194–211. doi: 10.1002/emmm.201302948 (PMC3927955; doi:10.1002/emmm.201302948)
Supplement: Supplementary file 5 [file emmm0006-0194-sd5.pdf]

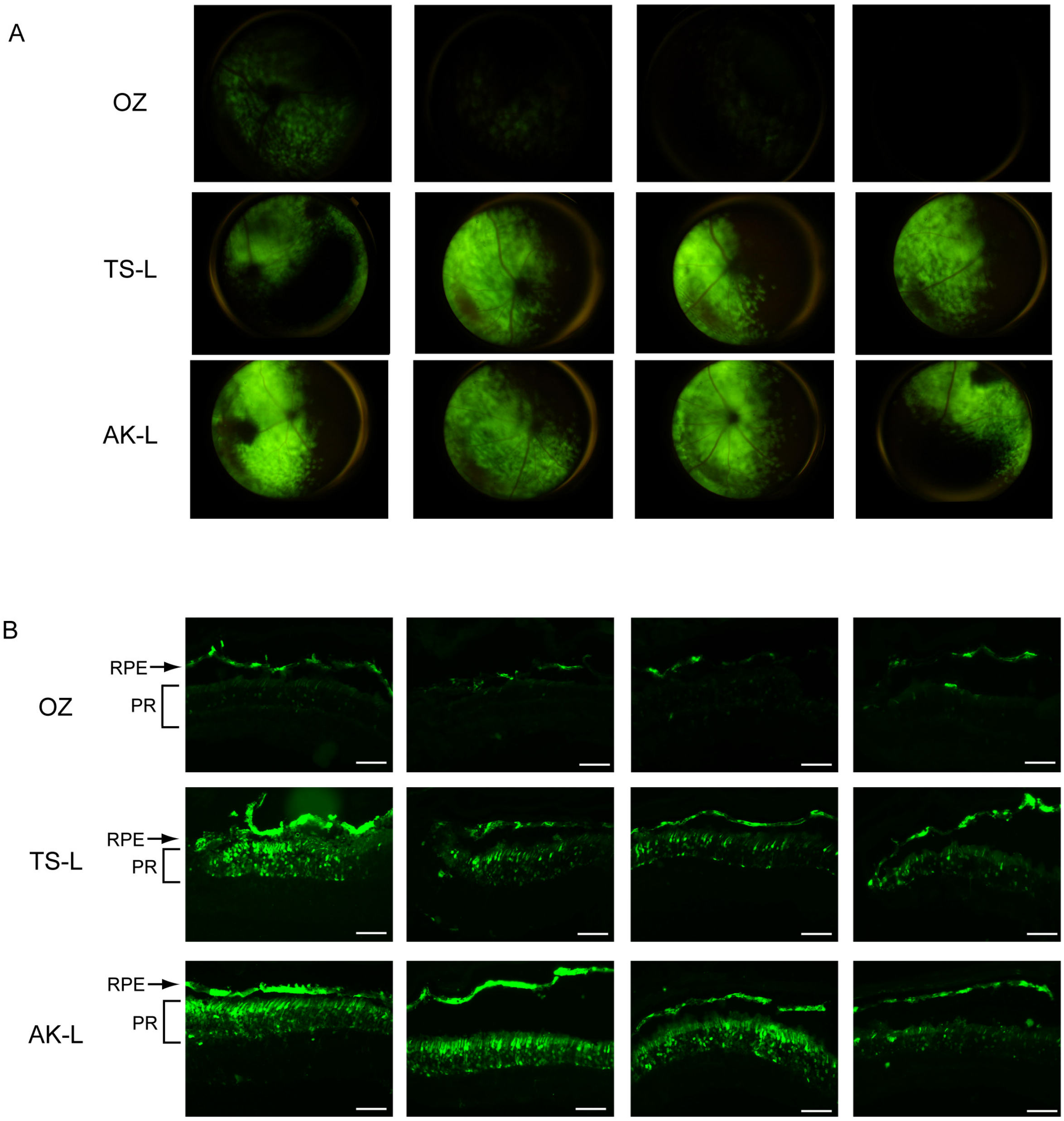

Supporting Figure 4. Dual AAV trans-splicing and hybrid AK vectors provide the most robust transduction of RPE and photoreceptors cell layers following subretinal delivery in mice. Live-imaging fundus fluorescence (A) and fluorescence analysis of retinal cryosections (B) from C57BL/6 mice one month following subretinal injection of AAV2/8 vectors encoding for EGFP under the control of the ubiquitous cytomegalovirus (CMV) promoter. (B) Arrows point at transduced RPE. The scale bar (200 µm) is depicted in the figure. OZ: AAV oversize (n=4); TS-L: dual AAV trans-splicing EGFP with a combined genome size similar to OZ-EGFP (n=4); AK-L: dual AAV hybrid AK EGFP with a combined genome size similar to OZ-EGFP (n=4); RPE: retinal pigmented epithelium; PR: photoreceptors.
