# Supplementary material for: Effective delivery of large genes to the retina by dual AAV vectors
Source: EMBO Mol Med. 2013 Dec 16;6(2):194–211. doi: 10.1002/emmm.201302948 (PMC3927955; doi:10.1002/emmm.201302948)
Supplement: Supplementary file 6 [file emmm0006-0194-sd6.pdf]

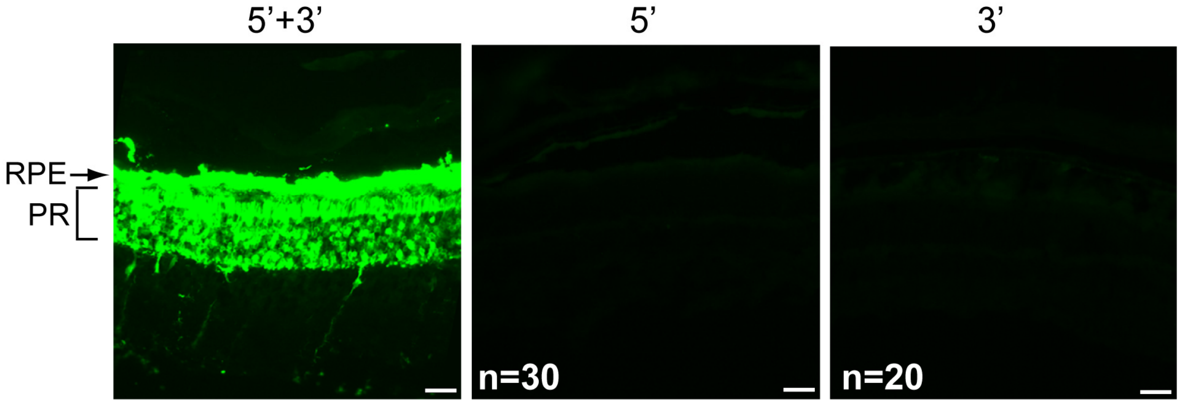

Supporting Figure 5. No detectable EGFP fluorescence in retinas injected with either the 5’- or 3’-half of dual AAV vectors.

Fluorescence analysis of representative retinal cryosections from C57BL/6 mice one month following subretinal injection of either the combination of dual AAV vectors (5’+3’) encoding for EGFP or each of the single 5’- and 3’-half vectors. The eyes injected with the 5’-half include: 9 injected with CMV 5’AK, 6 injected with CMV 5’TS, 8 injected with RHO 5’AK, 7 injected with RHO 5’TS (n=30). The eyes injected with the 3’-half include: 13 injected with 3’AK and 7 injected with 3’TS (n=20). The arrow points at transduced RPE. The scale bar (20 μm) is depicted in the figure. 5’+3’: retinas co-injected with 5’- and 3’-half vectors; 5’: retinas injected with the 5’-half vector; 3’: retinas injected with the 3’-half vector; RPE: retinal pigmented epithelium; PR: photoreceptors.
