# Supplementary material for: Effective delivery of large genes to the retina by dual AAV vectors
Source: EMBO Mol Med. 2013 Dec 16;6(2):194–211. doi: 10.1002/emmm.201302948 (PMC3927955; doi:10.1002/emmm.201302948)
Supplement: Supplementary file 7 [file emmm0006-0194-sd7.pdf]

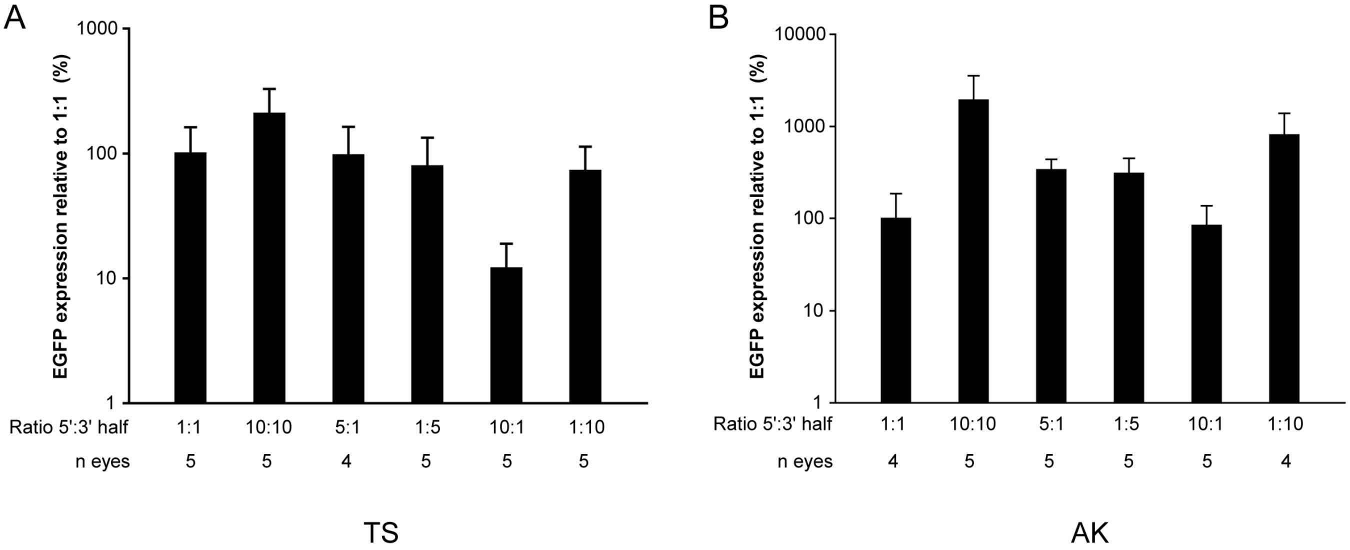

Supporting Figure 6. Murine retinal transduction with various doses and ratios of dual AAV vectors.

EGFP protein quantification by ELISA of eyecups from C57BL/6 mice one month following subretinal injection of dual AAV2/8 trans-splicing (A) and hybrid AK (B) vectors encoding for EGFP under the control of the ubiquitous cytomegalovirus (CMV) promoter. The various ratios of 5'- and 3'-half vectors used for the injections are depicted below each bar. The doses of 5'-and 3'-half vectors are the following: 1:1 [$2.5x10^8$:$2.5x10^8$ genome copies (GC)/eye]; 10:10 ($2.5x10^9$:$2.5x10^9$ GC/eye); 5:1 ($1.25x10^9$:$2.5x10^8$ GC/eye); 1:5 ($2.5x10^8$:$1.25x10^9$ GC/eye); 10:1 ($2.5x10^9$:$2.5x10^8$ GC/eye); 1:10 ($2.5x10^8$:$2.5x10^9$ GC/eye). The number (n) of eyes analyzed is depicted below each bar. The histograms show EGFP expression as percentage relative to the 1:1 ratio. Values are represented as mean ± s.e.m. (standard error of the mean). TS: dual AAV trans-splicing; AK: dual AAV hybrid AK. No statistically significant differences were found using ANOVA. More details on the statistical analysis including specific statistical values can be found in the Statistical analysis paragraph of the Materials and Methods section.
