# Supplementary material for: Effective delivery of large genes to the retina by dual AAV vectors
Source: EMBO Mol Med. 2013 Dec 16;6(2):194–211. doi: 10.1002/emmm.201302948 (PMC3927955; doi:10.1002/emmm.201302948)
Supplement: Supplementary file 8 [file emmm0006-0194-sd8.pdf]

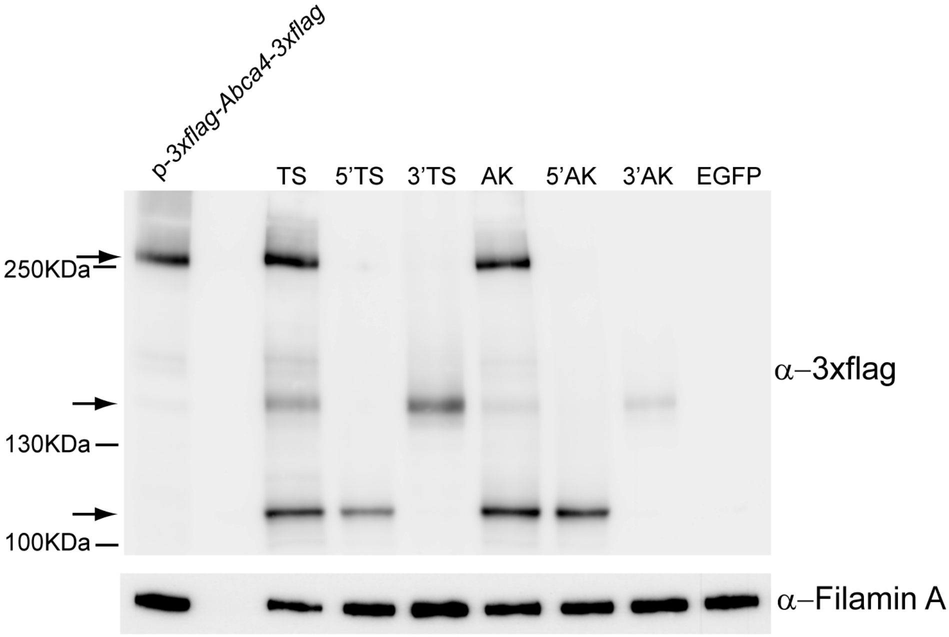

Supporting Figure 7. ABCA4 proteins smaller than expected are produced in vitro by dual AAV trans-splicing and hybrid AK vectors as well as by their corresponding single 5'- and 3'-half vectors.

Representative Western blot analysis of HEK293 cells infected with dual AAV2/2 trans-splicing (TS) and hybrid AK (AK) vectors encoding for ABCA4 under the control of the cytomegalovirus (CMV) promoter. Anti-3xflag antibodies recognize the 3xflag tag located at both the N- and C-termini of the ABCA4 protein. The upper arrow indicates the full-length ABCA4 protein; the lower arrows indicate the shorter products (>100 KDa) which derive from either single 5'- or 3'-half vectors. Thirty micrograms of proteins from transfected and infected cells were loaded; the molecular weight ladder is depicted on the left. The Western blot images are representative of n=3 independent experiments. p*3xflag-ABCA4-3xflag*: cells transfected with a plasmid encoding for full-length ABCA4 tagged with 3xflag at both N- and C-termini, as positive control; TS: cells infected with both 5'- and 3'-halves of dual AAV trans-splicing vectors; AK: cells infected with both 5'- and 3'-halves of dual AAV hybrid AK vectors; 5': cells infected with the 5'-half vector of either dual AAV TS (5'TS) or hybrid AK (5'AK) approaches; 3': cells infected with the 3'-half vector of either dual AAV TS (3'TS) or hybrid AK (3'AK) approaches; EGFP: cells infected with AAV vectors expressing EGFP, as negative control; α-3xflag: Western blot with anti-3xflag antibody; α-Filamin A: Western blot with anti-Filamin A antibody, used as loading control.
