# Supplementary material for: Effective delivery of large genes to the retina by dual AAV vectors
Source: EMBO Mol Med. 2013 Dec 16;6(2):194–211. doi: 10.1002/emmm.201302948 (PMC3927955; doi:10.1002/emmm.201302948)
Supplement: Supplementary file 9 [file emmm0006-0194-sd9.pdf]

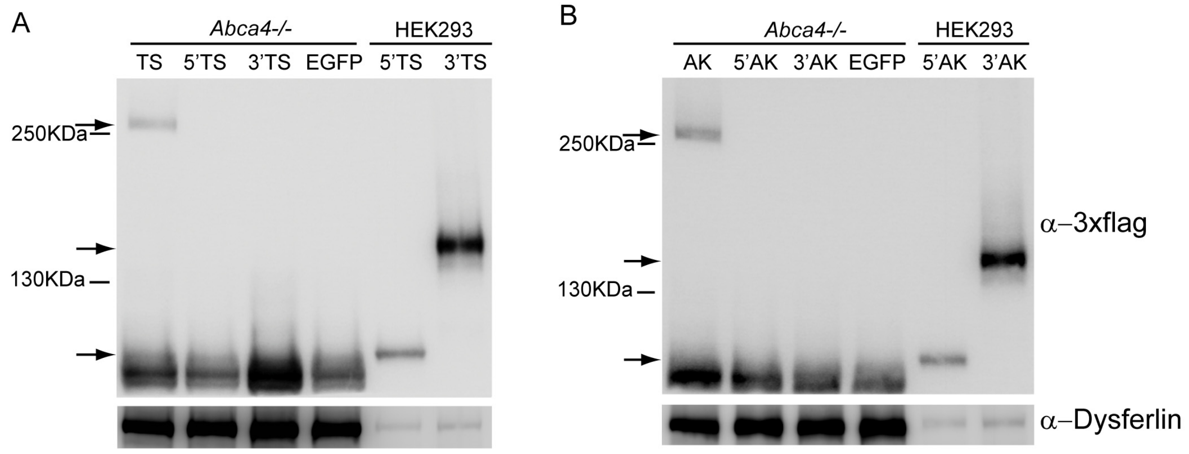

Supporting Figure 8. ABCA4 products of the expected size are detected in the eyes of C57BL/6 mice following subretinal delivery of dual AAV trans-splicing and hybrid AK vectors.

Representative Western blot analysis of C57BL/6 eyecups one month after subretinal delivery of dual AAV2/8 trans-splicing (TS; A) and hybrid AK (AK; B) vectors encoding for ABCA4 under the control of the ubiquitous cytomegalovirus (CMV) promoter. Anti-3xflag antibodies recognize the tag located at both the N- and C-termini of the ABCA4 protein. The lysates from HEK293 cells infected with single 5'- and 3'-half vectors of dual AAV2/2 TS and hybrid AK vectors were loaded as positive controls of the smaller than expected ABCA4 proteins observed in vitro (see Supp. Fig. 7). The upper arrow indicates the full-length ABCA4-3xflag; the lower arrows indicate the smaller products (>100KDa) which derive from either single 5'- or 3'-half vectors. Thirty or 150 micrograms of proteins from infected cells and injected eyecups were loaded, respectively; the molecular weight ladder is depicted on the left. The picture is representative of the following number of eyecups: n=10 treated with TS; n=10 treated with AK; n=5 treated with 5'TS, n=5 treated with 5'AK, n=5 treated with 3'TS, n=5 treated with 3'AK, n=7 treated with EGFP. TS: eyes injected with both 5'- and 3'-halves of dual AAV TS vectors; AK: eyes injected with both 5'- and 3'-halves of dual AAV hybrid AK vectors; 5': eyes injected or cells infected with the 5'-half of either dual AAV TS (5'TS) or hybrid AK (5'AK) vectors; 3': eyes injected or cells infected with the 3'-half of either dual AAV TS (3'TS) or hybrid AK (3'AK) vectors; EGFP: eyes injected with AAV vectors expressing EGFP, as negative control; HEK293: lysates from HEK293 cells infected with AAV; α-3xflag: Western blot with anti-3xflag antibody; α-Dysferlin: Western blot with anti-Dysferlin antibody, used as loading control.
