# Supplementary material for: Effective delivery of large genes to the retina by dual AAV vectors
Source: EMBO Mol Med. 2013 Dec 16;6(2):194–211. doi: 10.1002/emmm.201302948 (PMC3927955; doi:10.1002/emmm.201302948)
Supplement: Supplementary file 10 [file emmm0006-0194-sd10.pdf]

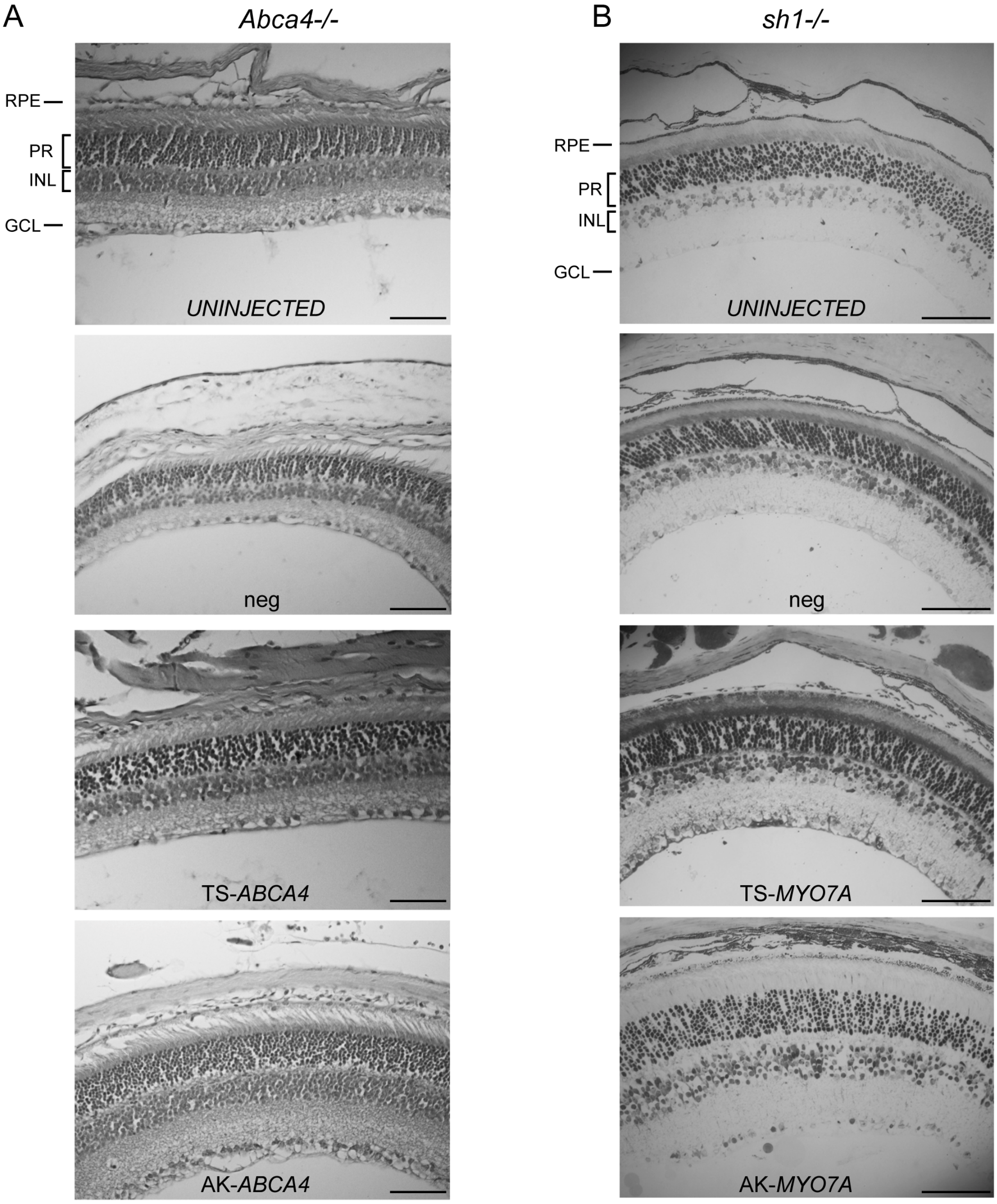

Supporting Figure 9. Normal retinal histology in Abca4-/- and sh1-/- mice following subretinal delivery of dual AAV trans-splicing and hybrid AK vectors.

(A) Representative paraffin-embedded sections of *Abca4-/-* retinas either uninjected (n=2) or treated at one month of age with dual AAV2/8-RHO-ABCA4 trans-splicing (TS-*ABCA4,* n=3), dual AAV hybrid AK (AK-*ABCA4*, n=4) or single 5'-half (neg) of either dual AAV TS (n=1) or hybrid AK (n=2; neg total n=3). Eyes were harvested 8 months after treatment and retinal sections were stained with hematoxylin and eosin. (B) Representative semi-thin sections of *sh1-/-* retinas either uninjected (n=2) or treated at one month of age with dual AAV2/8-CBA-MYO7A trans-splicing (TS-*MYO7A*, n=6), dual AAV hybrid AK (AK-*MYO7A*, n=6) or single 5'-half (neg) of either dual AAV TS (n=1) or hybrid AK (n=2; neg total n=3). Eyes were harvested 2-3 months after treatment and retinal sections were stained with toluidine blue. (A-B) the scale bar (50 μm) is depicted. RPE: retinal pigmented epithelium; PR: photoreceptors; INL: inner nuclear layer; GCL: ganglion cell layer.
