# Supplementary material for: Effective delivery of large genes to the retina by dual AAV vectors
Source: EMBO Mol Med. 2013 Dec 16;6(2):194–211. doi: 10.1002/emmm.201302948 (PMC3927955; doi:10.1002/emmm.201302948)
Supplement: Supplementary file 11 [file emmm0006-0194-sd11.pdf]

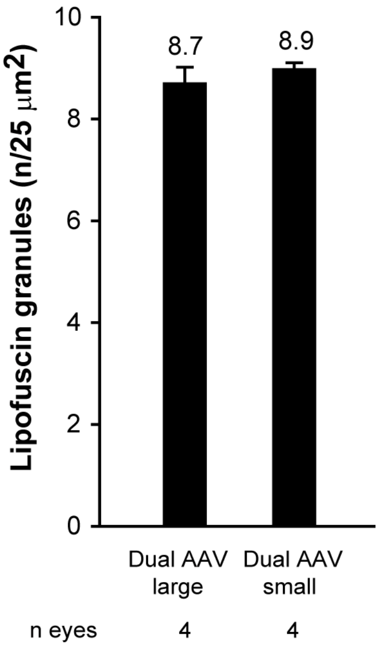

Supporting Figure 10. Similar lipofuscin granules accumulation in the retina of Abca4-/- mice independently of the AAV control vector genome size.

Quantification of lipofuscin granules in *Abca4-/-* mice injected subretinally with dual AAV trans-splicing and hybrid AK control vectors with a combined (5'-half+3'-half) large (8.9 Kb, dual AAV large) or small (2.7-2.9 Kb, dual AAV small) genome [dose of each vector/eye: $1.2x10^9$ genome copies (GC)]. Eyes were harvested 2 months post-injection and lipofuscin granules were counted in at least 30 fields (25 μm^2) for each sample. The number (n) of eyes analyzed is depicted below each bar. Dual AAV small includes *Abca4-/-* eyes injected with either trans-splicing small (n=2) or hybrid AK small (n=2; total n=4); dual AAV large includes *Abca4-/-* eyes injected with either trans-splicing large (n=2) or hybrid AK large (n=2; total n=4). Values are represented as mean ± s.e.m. (standard error of the mean). No statistically significant differences were found using the Student's t-test (p=0.54).
