# Supplementary material for: Effective delivery of large genes to the retina by dual AAV vectors
Source: EMBO Mol Med. 2013 Dec 16;6(2):194–211. doi: 10.1002/emmm.201302948 (PMC3927955; doi:10.1002/emmm.201302948)
Supplement: Supplementary file 12 [file emmm0006-0194-sd12.pdf]

RHO PROMOTER

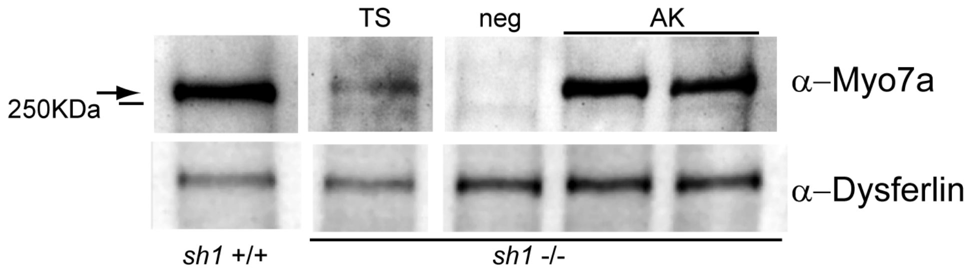

Supporting Figure 11. Subretinal administration of dual AAV trans-splicing and hybrid AK vectors results in MYO7A expression in photoreceptors.

Representative Western blot analysis of *sh1-/-* retinas 2 months following subretinal delivery of dual AAV trans-splicing (TS) and hybrid AK (AK) vectors encoding for MYO7A-HA under the control of the photoreceptor-specific Rhodopsin promoter (RHO PROMOTER). The arrow indicates full-length murine endogenous Myo7a in *sh1+/+* retinas and recombinant human MYO7A expressed from dual AAV vectors in *sh1-/-* retinas; the molecular weight ladder is depicted on the left; whole retina lysates were loaded in each lane. The picture is representative of: n=4 *sh1-/-* retinas treated with TS; n=5 *sh1-/-* retinas treated with dual hybrid AK. TS: retinas injected with dual AAV TS vectors; AK: retinas injected with dual hybrid AK vectors; neg: retinas injected with the 5'-half of either the dual AAV TS or hybrid AK vectors, as negative controls; α-Myo7a: Western blot with anti-Myosin7a antibody; α-Dysferlin: Western blot with anti-Dysferlin antibody, used as loading control.
