# Supplementary material for: Effective delivery of large genes to the retina by dual AAV vectors
Source: EMBO Mol Med. 2013 Dec 16;6(2):194–211. doi: 10.1002/emmm.201302948 (PMC3927955; doi:10.1002/emmm.201302948)
Supplement: Supplementary file 13 [file emmm0006-0194-sd13.pdf]

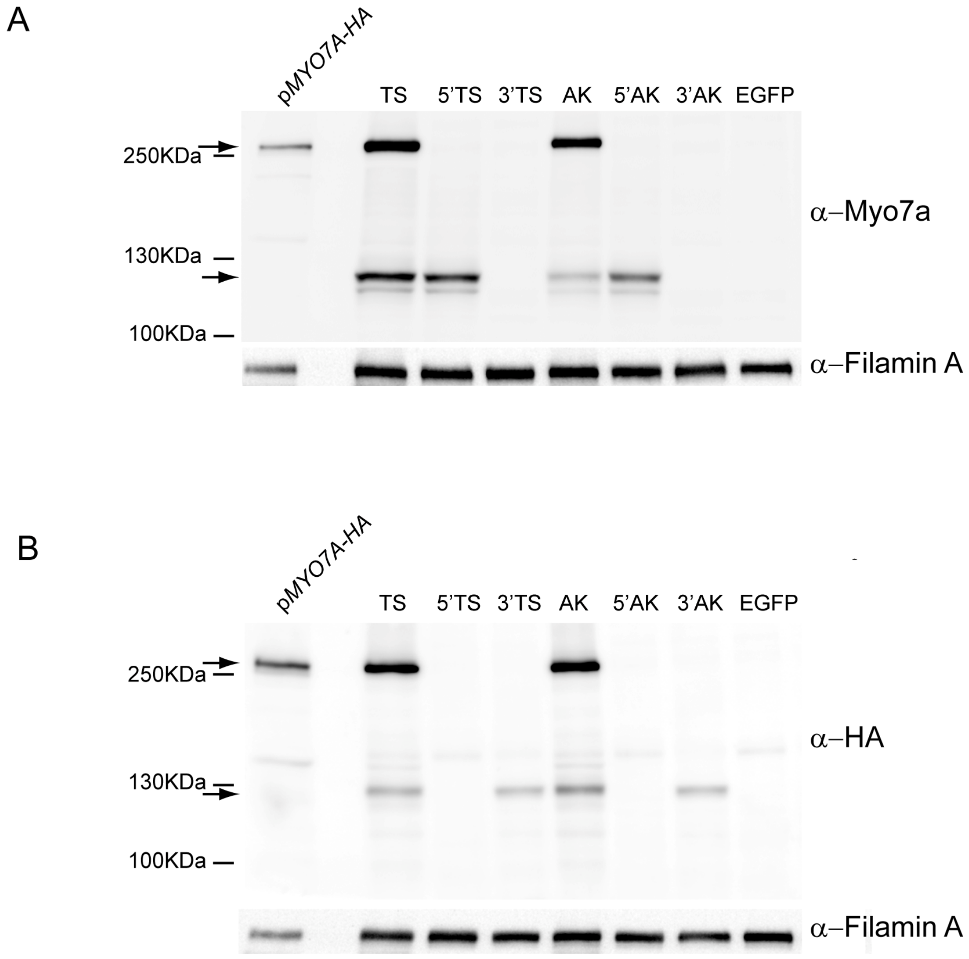

Supporting Figure 12. MYO7A proteins smaller than expected are produced in vitro by dual AAV trans-splicing and hybrid AK vectors as well as by their corresponding single 5'- and 3'-half vectors.

Representative Western blot analysis of HEK293 cells infected with dual AAV2/2 trans-splicing (TS) and hybrid AK (AK) vectors encoding for MYO7A-HA under the control of the chicken beta actin (CBA) promoter. Anti-Myo7a antibodies recognize an epitope contained in the 5'-half of the MYO7A coding sequence (A); anti-HA antibodies recognize the HA tag located at the MYO7A C-terminus and therefore contained in the 3'-half vector (B). The upper arrow indicates the full-length MYO7A-HA; the lower arrow indicates the smaller products (<130 KDa) which derive from either single 5'- (A) or 3'-halves (B); Fifteen or 40 micrograms of proteins from transfected and infected cells were loaded, respectively; the molecular weight ladder is depicted on the left. The Western blot pictures are representative of n=4 independent experiments. p*MYO7A-HA*: cells transfected with a plasmid encoding for full-length MYO7A-HA, as positive control; TS: cells infected with both 5'- and 3'-halves of dual AAV TS vectors; AK: cells infected with both 5'- and 3'-halves of dual AAV hybrid AK; 5': cells infected with the 5'-half of either dual AAV TS (5'TS) or hybrid AK (5'AK) vectors; 3': cells infected with the 3'-half of either dual AAV TS (3'TS) or hybrid AK (3'AK) vectors; EGFP: cells infected with AAV vectors expressing EGFP, as negative control; α-Myo7a: Western blot with anti-Myo7a antibody; α-HA: Western blot with anti-hemagglutinin (HA) antibody; α-Filamin A: Western blot with anti-Filamin A antibody, used as loading control.
