# Supplementary material for: Effective delivery of large genes to the retina by dual AAV vectors
Source: EMBO Mol Med. 2013 Dec 16;6(2):194–211. doi: 10.1002/emmm.201302948 (PMC3927955; doi:10.1002/emmm.201302948)
Supplement: Supplementary file 14 [file emmm0006-0194-sd14.pdf]

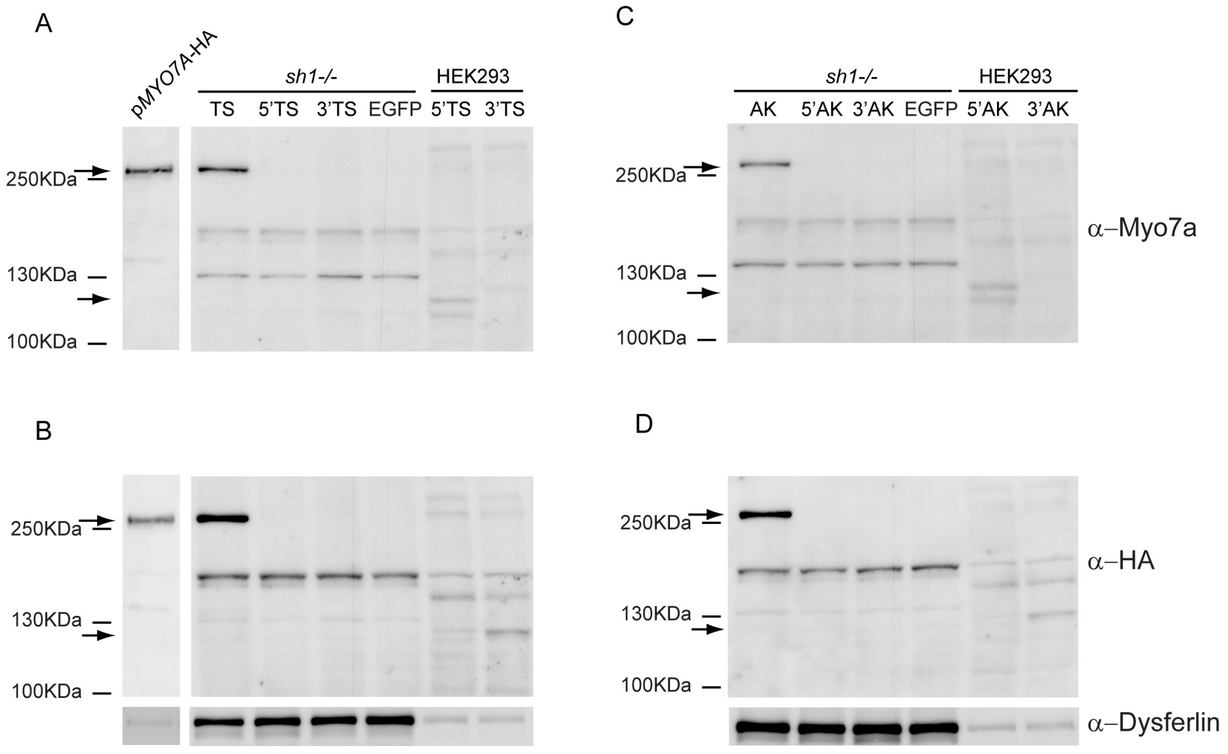

Supporting Figure 13. MYO7A products of the expected size are detected in the eyes of sh1-/- mice following subretinal delivery of dual AAV trans-splicing and hybrid AK vectors.
Representative Western blot analysis of albino *sh1-/-* eyecups 1.5 months after subretinal delivery of dual AAV2/8 trans-splicing (TS; A-B) and hybrid AK (AK; C-D) vectors encoding for MYO7A-HA under the control of the ubiquitous chicken beta-actin (CBA) promoter. Anti-Myo7a antibodies recognize an epitope contained in the 5'-half of the MYO7A coding sequence (A, C); anti-HA antibodies recognize the HA tag located at the MYO7A C-terminus and therefore contained in the 3'-half vector (B, D). The lysates from HEK293 cells infected with single 5'- and 3'-half vectors of dual AAV2/2-CBA-*MYO7A*-TS and hybrid AK vectors were loaded as positive controls of the smaller than expected MYO7A proteins observed in vitro (see Supp. Fig. 12). The upper arrow indicates the full-length MYO7A-HA; the lower arrow indicates the smaller products (<130 KDa) which derive from either single 5'- (A, C) or 3'-half (B, D) vectors. Sixty or 120 micrograms of proteins from infected cells and injected eyecups were loaded, respectively; the molecular weight ladder is depicted on the left. The picture is representative of the following number of eyecups: n=6 treated with TS; n=6 treated with AK; n=3 treated with 5'TS, n=2 treated with 5'AK, n=2 treated with 3'TS, n=2 treated with 3'AK, n=2 treated with EGFP. p*MYO7A-HA*: cells transfected with a plasmid encoding for full-length MYO7A-HA (15 micrograms of proteins were loaded as positive control). TS: eyes injected with both 5'- and 3'- halves of dual AAV TS vectors; AK: eyes injected with both 5'- and 3'- halves of dual AAV hybrid AK vectors; 5': eyes injected or cells infected with the 5'-half of either dual AAV TS (5'TS) or hybrid AK (5'AK) vectors; 3': eyes injected or cells infected with the 3'-half of either dual AAV TS (3'TS) or hybrid AK (3'AK) vectors; EGFP: eyes injected with AAV vectors expressing EGFP, as negative control; HEK293: lysates from HEK293 cells infected with AAV vectors; α-Myo7a: Western blot with anti-Myo7a antibody; α-HA: Western blot with anti-hemagglutinin (HA) antibody; α-Dysferlin: Western blot with anti-Dysferlin antibody, used as loading control.
