# Supplementary material for: Effective delivery of large genes to the retina by dual AAV vectors
Source: EMBO Mol Med. 2013 Dec 16;6(2):194–211. doi: 10.1002/emmm.201302948 (PMC3927955; doi:10.1002/emmm.201302948)
Supplement: Supplementary file 15 [file emmm0006-0194-sd15.pdf]

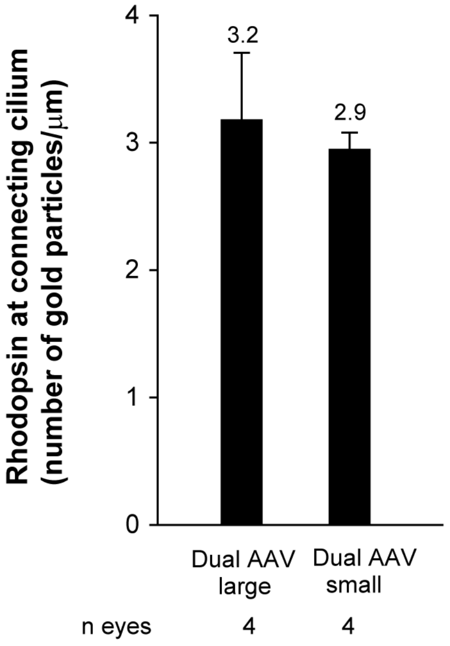

Supporting Figure 14. Similar rhodopsin accumulation at the connecting cilium of sh1-/- mice independently of the AAV control vector genome size.

Quantification of the number of rhodopsin gold particles at the PR connecting cilium of albino *sh1-/-* mice injected subretinally with dual AAV trans-splicing and hybrid AK control vectors with a combined (5'-half+3'-half) large (8.9 Kb, dual AAV large) or small (2.7-2.9 Kb, dual AAV small) genome [dose of each vector/eye: $1.7x10^9$ genome copies (GC)]. Eyes were harvested 2 months post-injection. The number (n) of eyes analyzed is depicted below each bar. Dual AAV small includes *sh1-/-* eyes injected with either trans-splicing small (n=2) or hybrid AK small (n=2; total n=4); dual AAV large includes *sh1-/-* eyes injected with either trans-splicing large (n=2) or hybrid AK large (n=2; total n=4). Values are represented as mean ± s.e.m. (standard error of the mean). No statistically significant differences were found using the Student's t-test (p=0.7). Control *sh1+/-* albino mice have 0.69 rhodopsin gold particles/μm at the connecting cilium. In agreement with Colella et al. (PLoS One. 2013 Aug 26;8(8)), the number of rhodopsin gold particles at the connecting cilium is higher in albino than in pigmented *sh1*-/-mice (Fig. 8C).
