# Supplementary material for: Effective delivery of large genes to the retina by dual AAV vectors
Source: EMBO Mol Med. 2013 Dec 16;6(2):194–211. doi: 10.1002/emmm.201302948 (PMC3927955; doi:10.1002/emmm.201302948)
Supplement: Supplementary file 16 [file emmm0006-0194-sd16.pdf]

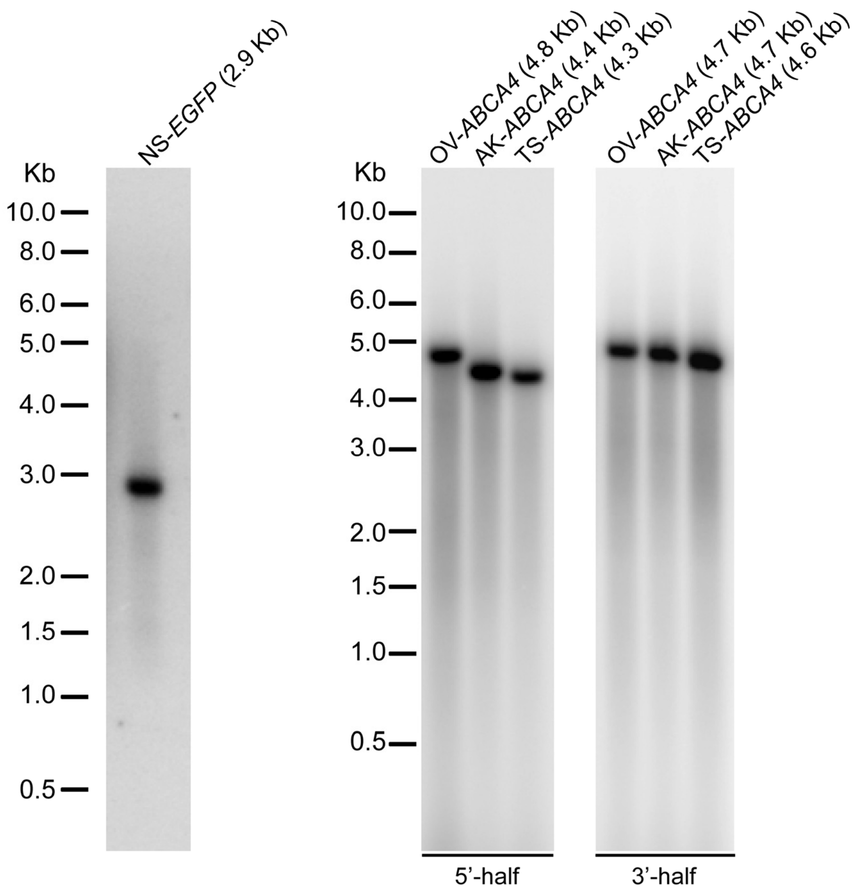

Supporting Figure 15. The genome of dual AAV RHO-ABCA4 vectors is correctly packaged in AAV capsids.
Alkaline Southern blot analysis of DNA extracted from $3x10^{10}$ genome copies of either single AAV of normal size (NS-EGFP) or dual AAV2/8-overlapping (OV-*ABCA4*), hybrid AK (AK-*ABCA4*) and trans-splicing (TS-*ABCA4*) vectors containing the RHO-*ABCA4-3xflag* expression cassette. The expected size of each genome is depicted in brackets above each corresponding lane. The molecular weight marker (Kb) is shown on the left. The probes used for the hybridization are described in the Materials and Methods section.
