# Supplementary material for: Effective delivery of large genes to the retina by dual AAV vectors
Source: EMBO Mol Med. 2013 Dec 16;6(2):194–211. doi: 10.1002/emmm.201302948 (PMC3927955; doi:10.1002/emmm.201302948)
Supplement: Supplementary file 17 [file emmm0006-0194-sd17.pdf]

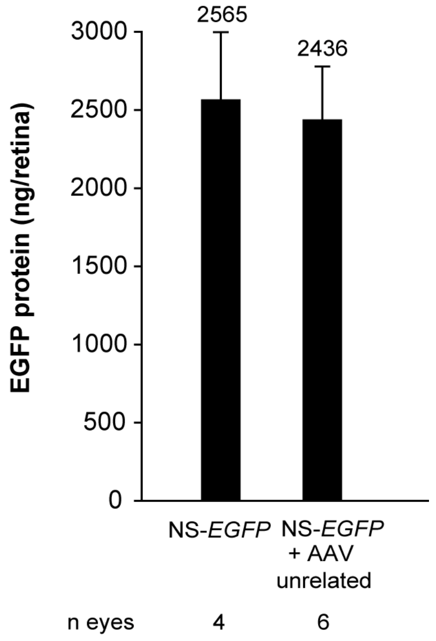

Supporting Figure 16. Similar EGFP levels following subretinal delivery of single AAV2/8-EGFP alone or in combination with the same dose of an unrelated AAV2/8 vector.

ELISA quantification of EGFP protein in eyecups from C57BL/6 mice one month following subretinal injection of single AAV2/8-CMV-EGFP vector of normal size (NS-*EGFP*) in combination or not with the same dose of a single AAV2/8 vector of normal size carrying an unrelated transgene expression cassette (unrelated AAV2/8). The number (n) of eyes analyzed is depicted below each bar. Values are represented as: mean ± s.e.m. (standard error of the mean). No statistically significant differences were found using the Student's t-test (p=0.8).
