# Supplementary material for: Effective delivery of large genes to the retina by dual AAV vectors
Source: EMBO Mol Med. 2013 Dec 16;6(2):194–211. doi: 10.1002/emmm.201302948 (PMC3927955; doi:10.1002/emmm.201302948)
Supplement: Supplementary file 18 [file emmm0006-0194-sd18.pdf]

Supporting Table 1. Plasmids for AAV vector production.

	Plasmid	Size ITR- ITR (bp)	AAV serotype	
			**2/2**	**2/8**
**Normal Size (NS)**	pZac2.1-CMV-*EGFP*-SV40	3006	**X**	**X**
	pZac2.1-CMV-*RFP*-SV40	2653		**X**
	pAAV-CMV-*DsRed*-HGH	2828		**X**
	pZac2.1-RHO-*EGFP*-SV40	2900		**X**
**Oversize (OZ)**	pAAV2.1-CMV-*EGFP*-9.9-SV40	9954	**X**	**X**
	pZac2.1-CMV-*ABCA4*_3xflag-SV40	8619	**X**	
	pAAV2.1-CBA-*MYO7A*_HA-BGH	8220	**X**	
**Overlapping (OV)**	pZac2.1-CMV-*ABCA4*_5'	4900	**X**	**X**
	pZac2.1-RHO-*ABCA4*_5'	4805		**X**
	pZac2.1-RHOK-*ABCA4*_5'	4169		**X**
	pZac2.1-VMD2-*ABCA4*_5'	4658		**X**
	pAAV2.1-CBA-*MYO7A*_5'	4708	**X**	**X**
	pAAV2.1-RHO-*MYO7A*_5'	4699		**X**
	pZac2.1-*ABCA4*_3'_3xflag_SV40	4740	**X**	**X**
	pAAV2.1-*MYO7A*_3'_HA_BGH	4655	**X**	**X**
**Trans-splicing (TS)**	pZac2.1-CMV-*ABCA4* _5'TS	4431	**X**	
	pZac2.1-CMV-3xflag-*ABCA4* _5'TS	4497	**X**	**X**
	pZac2.1-RHO-*ABCA4*_ 5'TS	4321		**X**
	pZac2.1-*ABCA4* _3'TS_3xflag_SV40	4587	**X**	**X**
	pAAV2.1-CBA-*MYO7A* _5'TS	4468	**X**	**X**
	pAAV2.1-RHO-*MYO7A* _5'TS	4459		**X**
	pAAV2.1-*MYO7A*_3'TS_HA_BGH	4298	**X**	**X**
	pZac2.1-CMV-*EGFP*_5'TS	1906	**X**	**X**
	pZac2.1-CMV-*EGFP*_5'TS-L	4016	**X**	**X**
	pZac2.1-RHO-*EGFP*_5'TS	1802		**X**
	pZac2.1-*EGFP*_3'TS_SV40-	1510	**X**	**X**
	pZac2.1-*EGFP*_3'TS_SV40-L	4551	**X**	**X**

	Plasmid	Size ITR- ITR (bp)	AAV serotype	
			2/2	2/8
Hybrid AP (AP)	pZac2.1-CMV-*ABCA4*_5'AP	4708	X	
	pZac2.1-*ABCA4* _3'AP_3xflag_SV40	4871	X	
	pAAV2.1-CBA-*MYO7A*_5'AP	4746	X	
	pAAV2.1-*MYO7A*_3'AP_HA_BGH	4576	X	
Hybrid AK (AK)	pZac2.1-CMV-*ABCA4* _5'AK	4540	X	
	pZac2.1-CMV-3xflag-*ABCA4* _5'AK	4606	X	X
	pZac2.1-RHO-*ABCA4*_ 5'AK	4436		X
	pZac2.1-*ABCA4* _3'AK_3xflag_SV40	4702	X	X
	pZac2.1-*ABCA4*_ 3'AK_HA_SV40	4663		X
	pAAV2.1-CBA-*MYO7A*_5'AK	4577	X	X
	pAAV2.1-RHO-*MYO7A* _5'AK	4568		X
	pAAV2.1-*MYO7A*_3'AK_HA_BGH	4421	X	X
	pZac2.1-CMV-*EGFP*_5'AK	2015	X	X
	pZac2.1-CMV-*EGFP*_5'AK-L	4125	X	X
	pZac2.1-RHO-*EGFP*_5'AK	1911		X
	pZac2.1-*EGFP*_3'AK_SV40	1614	X	X
	pZac2.1-*EGFP*_3'AK_SV40-L	4660	X	X

N.B. CMV: cytomegalovirus promoter; RHO: human Rhodopsin promoter; RHOK: human Rhodopsin kinase promoter; VMD2: vitelliform macular dystrophy 2 promoter; EGFP: enhanced green fluorescent protein; RFP: red fluorescent protein; DsRed: Discosoma sp. red fluorescent protein; ABCA4: human ATP-binding cassette, sub-family A, member 4; MYO7A: human MYOSIN VIIA; SV40: simian virus 40 poly-adenilation signal; HGH: human growth hormone poly-adenylation signal; BGH: bovine growth hormone poly-adenylation signal; 3xflag: 3xflag tag; HA: hemagglutinin tag; AP: alkaline phosphatase recombinogenic region; AK: F1 phage recombinogenic region; L: dual AAV vectors containing a combined (5'+3') large (>5 Kb in length) genome.
