# Supplementary material for: Effective delivery of large genes to the retina by dual AAV vectors
Source: EMBO Mol Med. 2013 Dec 16;6(2):194–211. doi: 10.1002/emmm.201302948 (PMC3927955; doi:10.1002/emmm.201302948)
Supplement: Supplementary file 19 [file emmm0006-0194-sd19.pdf]

Supporting Table 2. The titers of dual AAV vectors are similar to those of regular AAV vectors of normal size.

	AAV2/2			AAV2/8		
	**NS[a] (GC[b]/mL)**	**5'-half (GC/mL)**	**3'-half (GC/mL)**	**NS (GC/mL)**	**5-half (GC/mL)**	**3'-half (GC/mL)**
**Average**	$3.8x10^{12}$	$8.3x10^{12}$	$4.2x10^{12}$	$9.8x10^{12}$	$1.1x10^{13}$	$6.7x10^{12}$
**s.e.m.[c]**	$6.8x10^{11}$	$1.6x10^{12}$	$9.6x10^{11}$	$2.3x10^{12}$	$1.3x10^{12}$	$1.4x10^{12}$
**n[d] preps**	20	31	19	20	47	27

N.B. [a]NS: normal size; [b]GC: genome copies; [c]s.e.m.: standard error of the mean; [d]n: number of AAV preparations. The 5'-half sample includes single 5'-halves of dual AAV overlapping, trans-splicing and hybrid vectors; the 3'-half sample includes single 3'-halves of dual AAV overlapping, trans-splicing and hybrid vectors. No statistically significant differences using ANOVA were found in the titers of NS AAV2/2 or AAV2/8 compared to those of dual AAV vectors. More details on the statistical analysis including specific statistical values can be found in the Statistical analysis paragraph of the Materials and Methods section.
