# Supplementary material for: Effective delivery of large genes to the retina by dual AAV vectors
Source: EMBO Mol Med. 2013 Dec 16;6(2):194–211. doi: 10.1002/emmm.201302948 (PMC3927955; doi:10.1002/emmm.201302948)
Supplement: Supplementary file 20 [file emmm0006-0194-sd20.pdf]

SUPPORTING INFORMATION LIST

Supporting Table 1. Plasmids for AAV vector production.

Supporting Table 2. The titers of dual AAV vectors are similar to those of regular AAV vectors of normal size.

Supporting Figure 1. In vitro transduction efficiency of dual AAV trans-splicing and hybrid AK vectors compared to single normal size AAV vector.

Supporting Figure 2. Photoreceptor co-transduction following subretinal combined delivery of single AAV-EGFP and -RFP vectors.

Supporting Figure 3. CMV, RHO and RHOK promoters drive transgene expression in murine photoreceptors.

Supporting Figure 4. Dual AAV trans-splicing and hybrid AK vectors provide the most robust transduction of RPE and photoreceptors cell layers following subretinal delivery in mice.

Supporting Figure 5. No detectable EGFP fluorescence in retinas injected with either the 5'- or 3'-half of dual AAV vectors.

Supporting Figure 6. Murine retinal transduction with various doses and ratios of dual AAV vectors.

Supporting Figure 7. ABCA4 proteins smaller than expected are produced in vitro by dual AAV trans-splicing and hybrid AK vectors as well as by their corresponding single 5'- and 3'-half vectors.

Supporting Figure 8. ABCA4 products of the expected size are detected in the eyes of C57BL/6 mice following subretinal delivery of dual AAV trans-splicing and hybrid AK vectors.

Supporting Figure 9. Normal retinal histology in Abca4-/- and sh1-/- mice following subretinal delivery of dual AAV trans-splicing and hybrid AK vectors.

Supporting Figure 10. Similar lipofuscin granules accumulation in the retina of Abca4-/- mice independently of the AAV control vector genome size.

Supporting Figure 11. Subretinal administration of dual AAV trans-splicing and hybrid AK vectors results in MYO7A expression in photoreceptors.

Supporting Figure 12. MYO7A proteins smaller than expected are produced in vitro by dual AAV trans-splicing and hybrid AK vectors as well as by their corresponding single 5'- and 3'-half vectors.

Supporting Figure 13. MYO7A products of the expected size are detected in the eyes of sh1-/- mice following subretinal delivery of dual AAV trans-splicing and hybrid AK vectors.

Supporting Figure 14. Similar rhodopsin accumulation at the connecting cilium of sh1-/- mice independently of the AAV control vector genome size.

Supporting Figure 15. The genome of dual AAV RHO-ABCA4 vectors is correctly packaged in AAV capsids.

Supporting Figure 16. Similar EGFP levels following subretinal delivery of single AAV2/8-EGFP alone or in combination with the same dose of an unrelated AAV2/8 vector.
